# Mitochondrial diversity and inter-specific phylogeny among dolphins of the genus *Stenella* in the Southwest Atlantic Ocean

**DOI:** 10.1371/journal.pone.0270690

**Published:** 2022-07-14

**Authors:** Drienne Messa Faria, Debbie Steel, C. Scott Baker, José Martins da Silva, Ana Carolina Oliveira de Meirelles, Luciano Raimundo Alardo Souto, Salvatore Siciliano, Lupércio Araujo Barbosa, Eduardo Secchi, Juliana Couto Di Tullio, Larissa Rosa de Oliveira, Paulo Henrique Ott, Ana Paula Cazerta Farro

**Affiliations:** 1 Laboratório de Genética e Conservação Animal, Departamento de Ciências Agrárias e Biológicas, Universidade Federal do Espírito Santo (UFES), São Mateus, ES, Brazil; 2 Marine Mammal Institute, Hatfield Marine Science Center, Oregon State University, Newport, OR, United States of America; 3 Instituto Chico Mendes de Conservação da Biodiversidade (ICMBio), Fernando de Noronha, PE, Brazil; 4 Associação de Pesquisa e Preservação de Ecossistemas Aquáticos, AQUASIS, Caucaia, CE, Brazil; 5 Viver, Informar e Valorizar o Ambiente, (V.I.V.A), Itaigara, Salvador, BA, Brazil; 6 Departamento de Ciências Biológicas, Escola Nacional de Saúde Pública/Fiocruz, Rio de Janeiro, RJ, Brazil; 7 Organização Consciência Ambiental (ORCA/ES), Guarapari, ES, Brazil; 8 Laboratório de Ecologia e Conservação da Megafauna Marinha, Universidade Federal do Rio Grande (FURG), Rio Grande, RS, Brazil; 9 Grupo de Estudos de Mamíferos Aquáticos do Rio Grande do Sul (GEMARS), Torres, RS, Brazil; 10 Universidade Estadual do Rio Grande do Sul (Uergs), Osório, RS, Brazil; Natural History Museum of London, UNITED KINGDOM

## Abstract

The genus *Stenella* is comprised of five species occurring in all oceans. Despite its wide distribution, genetic diversity information on these species is still scarce especially in the Southwest Atlantic Ocean. Some features of this genus can enhance opportunities for potential introgressive hybridization, e.g. sympatric distibution along the Brazilian coast, mixed known associations among species, karyotype uniformity and genome permeability. In this study we analyzed three genes of the mitochondrial genome to investigate the genetic diversity and occurrence of genetic mixture among eighty specimens of *Stenella*. All species exhibited moderate to high levels of genetic diversity (h = 0.833 to h = 1.000 and *π* = 0.006 to *π* = 0.015). Specimens of S. *longirostris*, *S*. *attenuata and S*. *frontalis* were clustered into differentiated haplogroups, in contrast, haplotypes of *S*. *coeruleoalba* and *S*. *clymene* were clustered together. We detected phylogenetic structure of mixed clades for *S*. *clymene* and *S*. *coeruleoalba* specimens, in the Southwest Atlantic Ocean, and also between *S*. *frontalis* and *S*. *attenuata* in the Northeast Atlantic Ocean, and between *S*. *frontalis* and *S*. *longirostris* in the Northwest Atlantic Ocean. These specimes were morphologically identified as one species but exhibited the maternal lineage of another species, by mitochondrial DNA. Our results demonstrate that ongoing gene flow is occurring among species of the genus *Stenella* reinforcing that this process could be one of the reasons for the confusing taxonomy and difficulties in elucidating phylogenetic relationships within this group.

## Introduction

*Stenella* is one of the most abundant and widely distributed genus of the Delphinidae family and is comprised of five species: pantropical spotted dolphin (*Stenella attenuata* [Gray, 1846]), striped dolphin (*Stenella coeruleoalba* [Mayen, 1833]), spinner dolphin (*Stenella longirostris* [Gray, 1828]), clymene dolphin (*Stenella clymene* [Gray, 1850]) and atlantic spotted dolphin (*Stenella frontalis* [G. Cuvier, 1829]). While the first three species exhibit a pantropical distribution, occurring in all the world’s oceans, the last two are restricted to the waters of the Atlantic Ocean.

Previous genetic studies of *Stenella* species in the Southwest Atlantic Ocean (SWA) have desmonstrated low to high levels of mitochondrial DNA (mtDNA) genetic diversity depending on the species and region studied. Low levels of diversity were described in the population of *S*. *longisrotris* in the Fernando de Noronha archipelago of Brazil (h = 0.374; π = 0.044) [1] while high levels were found for *S*. *clymene* (h = 1.00; π = 0.02) [2], and for *S*. *frontalis* (h = 1.00; π = 0.027) [3].

Several studies have demonstrated the difficulty in resolving the phylogenetic relationships of *Stenella* species using molecular methods (mitochondrial and/ or nuclear DNA) [4–10]. Delphinid species are thought to have arisen through rapid radiation around the mid to late Miocene (11–15 mya) [8]. The subfamily Delphininae arose more recently in a rapid radiation event during the Pliocene [6,8]. Moreover, there is consensus that the genus *Stenella* is paraphyletic [4,5]. Ongoing hybridization and incomplete lineage sorting are both thought to be reasons for difficulties in reconstructing phylogenetic relationships, inferred by genetic data, among dolphin species of the family Delphinidae [8]. Despite being considered as an “evolutionary accident” by traditional zoologists, introgression between species seems to be a regular process in nature [11]. Hybridization can provide greater adaptability to environmental changes allowing hybrids to exploite new niches, although hybrid speciaion is necessarily rare in nature [12,13].

Cetaceans (whales and dolphins) exhibit characteristics that may allow for the production of viable wild hybrids, such as prominent karyotype uniformity and genome permeability [14]. Additionally, all five species of *Stenella* are found off the Brazilian coast in the Southwest Atlantic Ocean (SWA) allowing for the possibility of hybridization between these species in this region [15]. Hybridization has been documented between several species of cetaceans both in captivity and in the wild with the use of morphological [16–19], genetic evidence [14,20–27] or both [28]. However, there can be difficulties in some taxa, with the occurrence of cryptic hybrids that may have exactly the same morphotype as one of the parental species [17]. In these cases, confident identification is only possible with the use of molecular tools [11]. The recognition of hybrids between some cetacean species can be even more challenging due to an overlap in the range of intra- and interspecific variation of some morphological traits [17].

Molecular and morphometric data of *Stenella* specimens from the North Atlantic, Pacific and Indian oceans supported the hypothesis that *S*. *clymene* is the result of historical hybridization between *S*. *coeruleoalba* and *S*. *longirostris* [26]. Molecular data also demonstrate natural hybridization between *S*. *coeruleoalba* and *Delphinus delphis* in the Greek Seas [24]. Onboard observations and underwater photographs of groups of dolphins in the coastal waters of the Fernando de Noronha archipelago (545 km off the Brazilian coast) have indicated the occurrence of two possible hybrid individuals in this region: one presenting morphological features of *S*. *longirostris* and *S*. *attenuata*; and another presenting morphological features of *S*. *longirostris* and *S*. *clymene* [29].

The Mitochondrial DNA (mtDNA) is an effective molecular marker for the quantification of genetic diversity, and together with nuclear markers to detect reciprocal hybrids [1,24]. Mitochondrial DNA is maternally inherited, has high mutational rates and it is easy to isolate and characterize [30,31]. Due to the oceanic distribution of species within the genus *Stenella*, the majority of samples have been opportunistically collected from stranded dead dolphins and as such, the DNA derived from these samples can be of low-quality [32,33]. Therefore, previous studies of species of the genus *Stenella* have primarily used mtDNA for their analyses [1,3].

Molecular identification using mtDNA has also been used to identify many cetacean species. Databases, such as the Barcode of Life Data System (Bold) [34], GenBank [35] and DNA Surveillance [36], contain sequences from most known cetacean species and can be used to help the molecular identification at the species level, however, it is understood that mtDNA, alone, can fail in the identification of some cetacean’s species and cannot confirm the hybrid origin of an individual, especially when species share lineages due to incomplete lineage sorting [37–40]. Here we used mtDNA sequences and morphological identification, where available, to investigate the genetic diversity and possible genetic mixture among the species of *Stenella* that occur off the Brazilian coast. Understanding the gentic diversity and investigating genetic mixture within this genus is important to elucidate taxonomic uncertainties of species that have recently diverged and to assist in delineating conservation strategies of populations.

## Materials and methods

### Sampling

**Fig 1:**
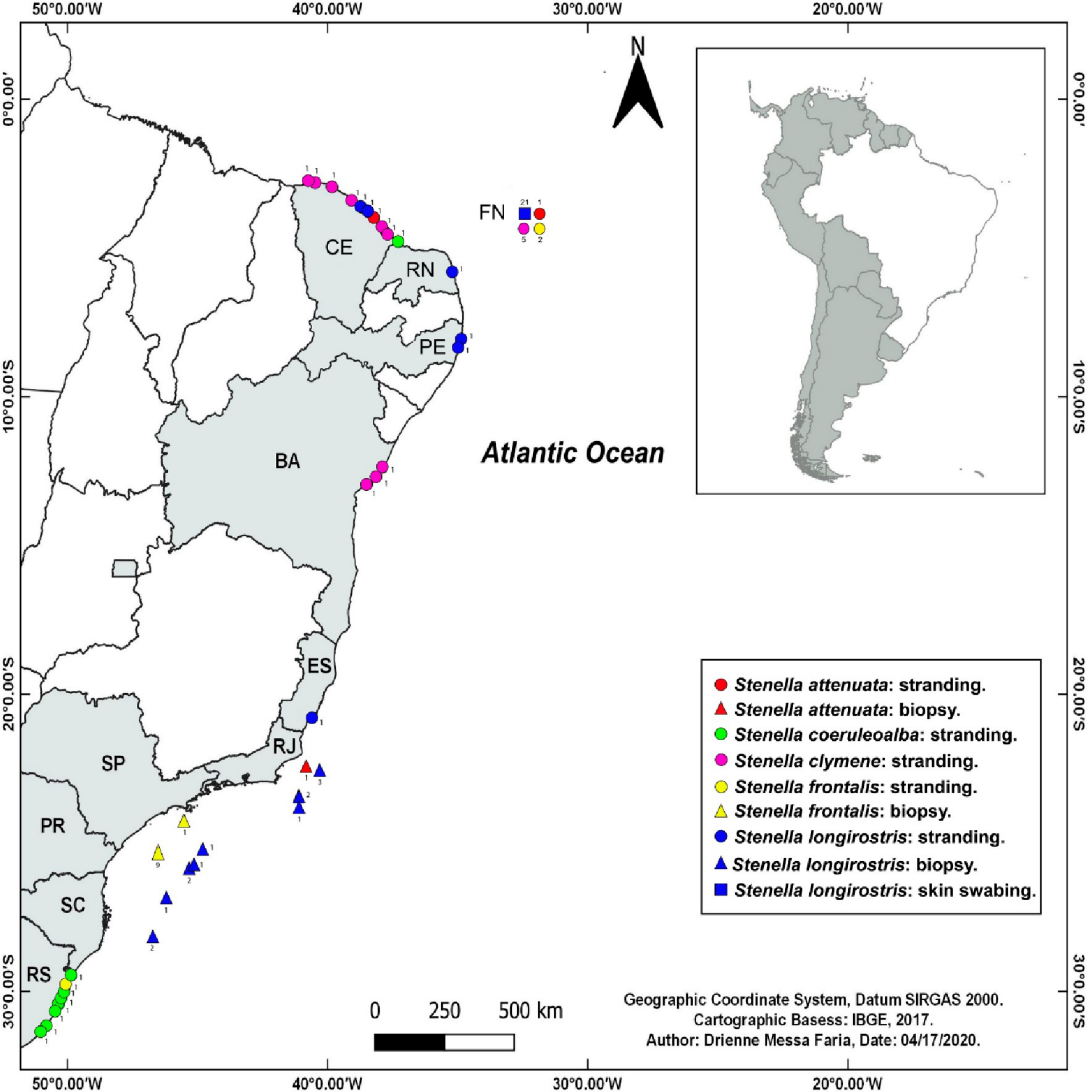
Locations where *Stenella* samples were obtained on the Brazilian waters. In gray, sampled states: CE: Ceará; FN: Fernando de Noronha; RN: Rio Grande do Norte; PE: Pernamuco; BA: Bahia; ES: Espírito Santo; RJ: Rio de Janeiro; SP: São Paulo; PR: Paraná; SC: Santa Catarina; RS: Rio Grande do Sul. This map was made using the QGIS software (QGIS.org, 2021; QGIS Geographic Information System; QGIS Association; http://www.qgis.org).

Eighty tissue samples (skin or muscle) were collected from the five species of *Stenella* found along the Brazilian coast and offshore: *S*. *attenuata* (N = 4), *S*. *coeruleoalba* (N = 8), *S*. *clymene* (N = 14), *S*. *frontalis* (N = 14), *S*. *longirostris* (N = 40). Samples were collected from dolphins at sea, through skin swabbing with a biopsy dart, as well as from stranded animals (Fig 1 and S1 Table).

The skin samples of *Stenella longirostris* from the Fernando de Noronha archipelago were collected through skin swabbing and the samples from the coast of Brazil were collected through biopsies [41–43] These two techniques are minimally invasive, result in little apparent disturbance and are commonly used for acquisition of biological material from cetaceans. The samples were sent to and stored in the Laboratório de Genética e Conservação Animal, Universidade Federal do Espírito Santo. None of these species are considered endangered or protected by the World Conservation Union (IUCN, Red List of Threatened Species 2017). Licenses to collect, transport and manipulate biological material were provided by the “Sistema de Autorização e Informação em Biodiversidade (SISBIO)/ Instituto Chico Mendes de Biodiversidade (ICMBio)” under SIBIO license number 16586–2 and all procedures performed involving animals were in accordance with the ethical standards of the institution. The person in S7 Fig of this manuscript has given written informed consent (as outlined in PLOS consent form) to publish these case details.

All these specimens were identified by experienced or trained field correspondents following the standard procedures suggested by the American Society of Mammalogists published in 1987 in the protocol *Acceptable Field Methods in Mammalogy*: *Preliminary Guidelines Approved by the American Society of Mammalogists* (ad hoc Committee on Acceptable Field Methods in Mammalogy 1987, http://mammalogy.org/uploads/committee_files/ACUC1987.pdf) and by Geraci and Lounsbury (2005) [44].

### DNA extraction, amplification and mtDNA sequencing

Genomic DNA was extracted from muscle samples following a salt buffer protocol [45] and from skin samples using Chelex resin (SIGMA) according to manufacturer’s instructions. Three mitochondrial DNA (mtDNA) genes were amplified: the control region (Dloop), the coding genes of cytochrome b (Cytb) and cytochrome oxidase subunit I (CoxI). Dloop was amplified using KRAdLp 1.5 t-pro [46] and dlp5 [47] primers following the Polymerase Chain Reaction (PCR) conditions reported by Andrews et al., (2010) [48]; Cytb was amplified using L14724 [49] and H15387 [50] primers following the PCR conditions reported by Viricel et al., (2012) [51]; Cox1 was amplified using COXIF and COXIR primers following the PCR conditions reported by Amaral et al., (2007) [38]. Amplified fragments were sequenced in both directions, with an ABI 310 automated sequencer. To confirm the results for all possible species mixture identified, extractions, amplifications and sequencing were repeated three times.

### Analyses

Sequences of the three genes were edited manually and aligned separately using the algorithm Muscle within the program MEGA 6.06 [52]. Sequences were compared to BOLD, GenBank and DNA Surveillance databases to confirm species identity. All the sequences generated were uploaded to GenBank database (S2 Table).

The diversity indices for each species were estimated using Arlequin 3.5.2.2 [53]. Genealogical relationships among the haplotypes were inferred through Median-Joining analysis as implemented in the program Network v 4.6.1.0 [54]. Genetic distances among species were calculated using the Tamura–Nei distance model and 1000 bootstrap replications, including calculation of standard errors within the program MEGA 6.06 [52,55].

Phylogenetic analyses were conducted using sequence alignments for each mtDNA locus separately (Dloop, CoxI, Cytb). Only for the Brazilian coast and offshore sequences a concatenated matrix which combined all three genes were also used. Phylogenetic analyses were conducted using the program Beast v1.7.4 [56] under the following parameters: 100 million MCMC generations, sampling every 10.000 generations, Yule speciation model. The complete mitochondrial genome sequences of *Steno bredanensis* (JF339982), *Globicephala melas* (HM060334) and *Phocoena phocoena* (AJ554063) were used as outgroups in all sequence alignments for each mtDNA locus separately (Dloop, CoxI, Cytb).

Tracer v1.6 [57] was used to assess convergence and effective sample sizes (ESS) for all parameters: average standard deviation of split frequencies between chains below 0.01; potential scale reduction factor of all the estimated parameters with values of ∼1; plot of the generation versus the log probability of the data without noise (the log likelihood values); the minimum value of minimum Estimated Sample Sizes larger than 100 (values below 100 indicate that the parameter is under-sampled). The program TreeAnnotator v1.7.4 [56] was used to summarize the trees obtained into a single tree that best represents the posterior distribution, with a maximum clade credibility and a burn-in value of 1000 and posterior probability limit of 0.5. The program FigTree v1.4.2 [58] was used to produce and edit the phylogenetic tree figures.

For a worldwide phylogenetic comparison of mtDNA among ocean basins (Atlantic, Pacific and Indian), for each gene, we used all available sequences of *Stenella* in GenBank that were supported by information on geographic location (www.ncbi.nlm.nhi.gov/Genbank). This included 708 sequences of Dloop, 90 sequences of Cytb and 31 sequences of CoxI (S2 Table). For the sequences downloaded from GenBank we assumed the morphological species identity as described in the published paper or GenBank record. The names of the haplotypes used here in the cladograms follow the morphological species identity, as reported in the GenBank records. The molecular identity (Dloop, CoxI or Cytb identity) was determined by comparing the sequences with BOLD, GenBank and DNA Surveillance databases.

## Results

### Stenella dolphins from Brazilian waters

All species exhibited moderate to high levels of genetic variability when looking at all gene regions concatenated (Table 1). Haplotype diversity was highest in *S*. *coeruleolba* and in *S*. *clymene* (both h = 1) and lowest in *S*. *attenuata* (h = 0.833). Nucleotide diversity was highest in *S*. *clymene* (*π* = 0.015) and lowest in *S*. *frontalis* (*π* = 0.006) (Table 1). The rank of species for both indices varied depending on the gene being analyzed (S3 Table).

**Table 1:** Genetic diversity values of the five *Stenella* species from Brazilian waters for 1516 base pairs (bp) of mtDNA (Dloop + Cytb + CoxI). Sample size (N), number of haplotypes (Nh), polymorphic sites (PS), haplotype diversity (h), and nucleotide diversity (π).

Dloop + Cytb + CoxI (1516 bp)					
	N	Nh	Ps	h	*π*
*S*. *attenuata*	4	3	43	0.833	0.014
*S*. *clymene*	14	13	82	1.00	0.015
*S*. *coeruleoalba*	8	9	69	1.00	0.014
*S*. *frontalis*	14	9	30	0.912	0.006
*S*. *longirostris*	40	29	54	0.962	0.007

Genetic distances revealed values above 2% for almost all comparisons between species. Values below 2% were found between: *S*. *clymene* and *S*. *coeruleoalba* (Table 2) and also for genes analyzed individually (S4 Table).

**Table 2:** Genetic distances between *Stenella* species from Brazilian waters for 1516 bp of mtDNA (Dloop + Cytb + CoxI). Genetic distance values (%) are bellow diagonal, and, standard errors (SEs) are upper diagonal.

Dloop + Cytb + CoxI (1516 bp)					
	*S*. *attenuata*	*S*. *clymene*	*S*. *coeruleoalba*	*S*. *frontalis*	*S*. *longirostris*
*S*. *attenuata*		0.005	0.005	0.005	0.005
*S*. *clymene*	4.69%		0.002	0.003	0.005
*S*. *coeruleoalba*	4.44%	1.99		0.003	0.005
*S*. *frontalis*	4.32%	2.44	2.14		0.004
*S*. *longirostris*	4.49%	4.07	3.85	3.44	

**Fig 2:**
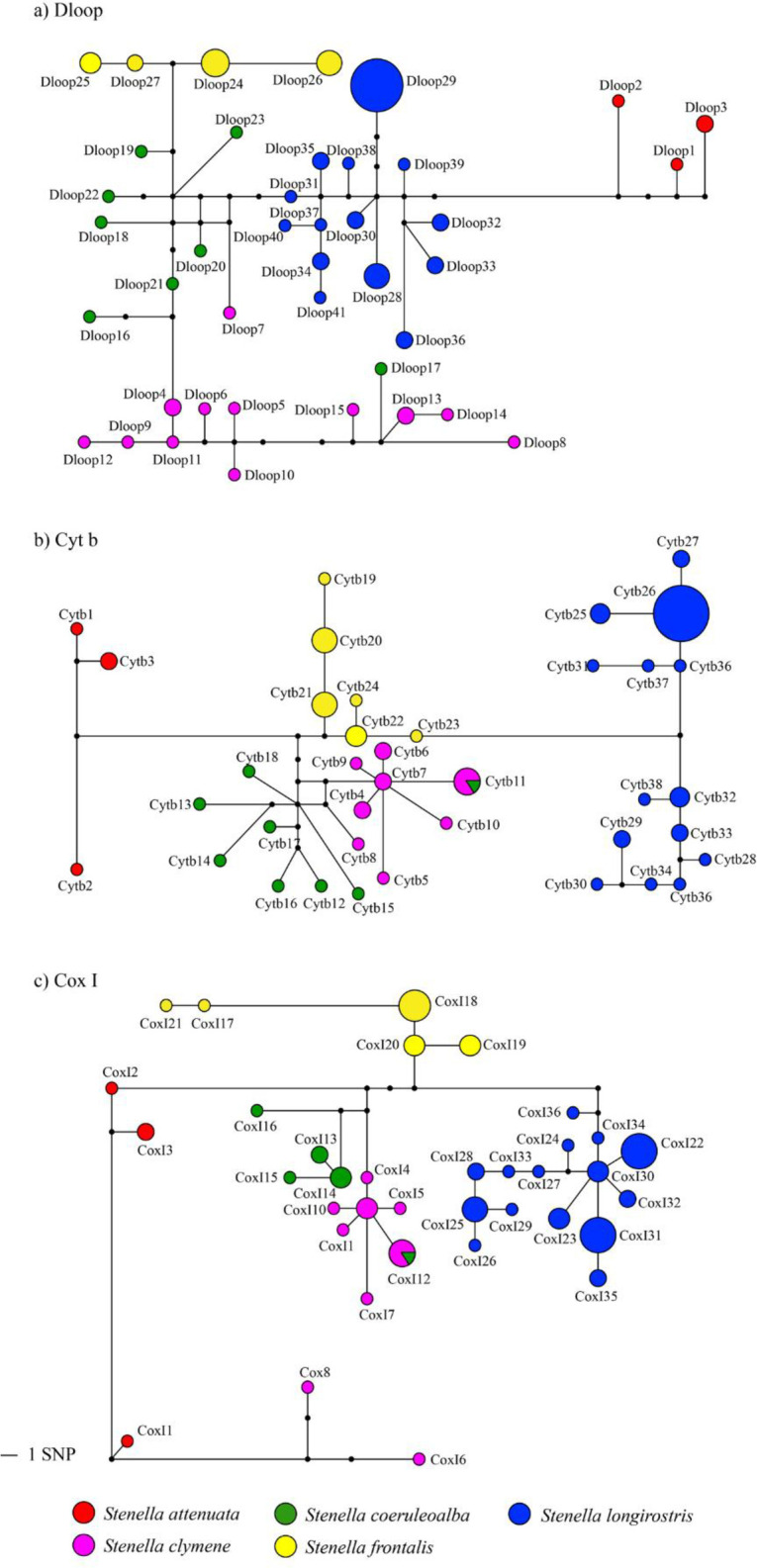
Median-Joining network of *Stenella* species from Brazilian waters for mtDNA control region (Dloop), cytochrome b (Cytb), and cytochrome oxidase subunit I (CoxI). Each circle corresponds to a haplotype, and its size is proportional to its frequency. Black circles indicate missing or intermediate haplotypes. Lengths of lines connecting haplotypes are proportional to the number of substitutions between haplotypes.

Haplotype networks for all three mtDNA genes showed clear separation of *S*. *attenuata*, *S*. *frontalis* and *S*. *longirostris* into different haplogroups with at least five mutational steps distinguishing them (Fig 2). In contrast, haplotypes of *S*. *coeruleoalba* and *S*. *clymene* were clustered together. Within the Dloop haplotype network one *S*. *clymene* haplotype (Dloop7) present in one specimen (Scl10) was nested within the *S*. *coeruleoalba* group, and one *S*. *coeruleoalba* haplotype (Dloop17) present in one specimen (Sco03) was nested within the *S*. *clymene* group. One Cytb haplotype (Cyt11) and one CoxI haplotype (CoxI12) were shared between *S*. *coeruleoalba* and *S*. *clymene*. The CoxI haplotype network also showed that two *S*. *clymene* haplotypes (CoxI8, present in specimen Scl10, and CoxI6 present in specimen Scl08) were very distant from the majority of haplotypes of this species (Fig 2).

**Fig 3:**
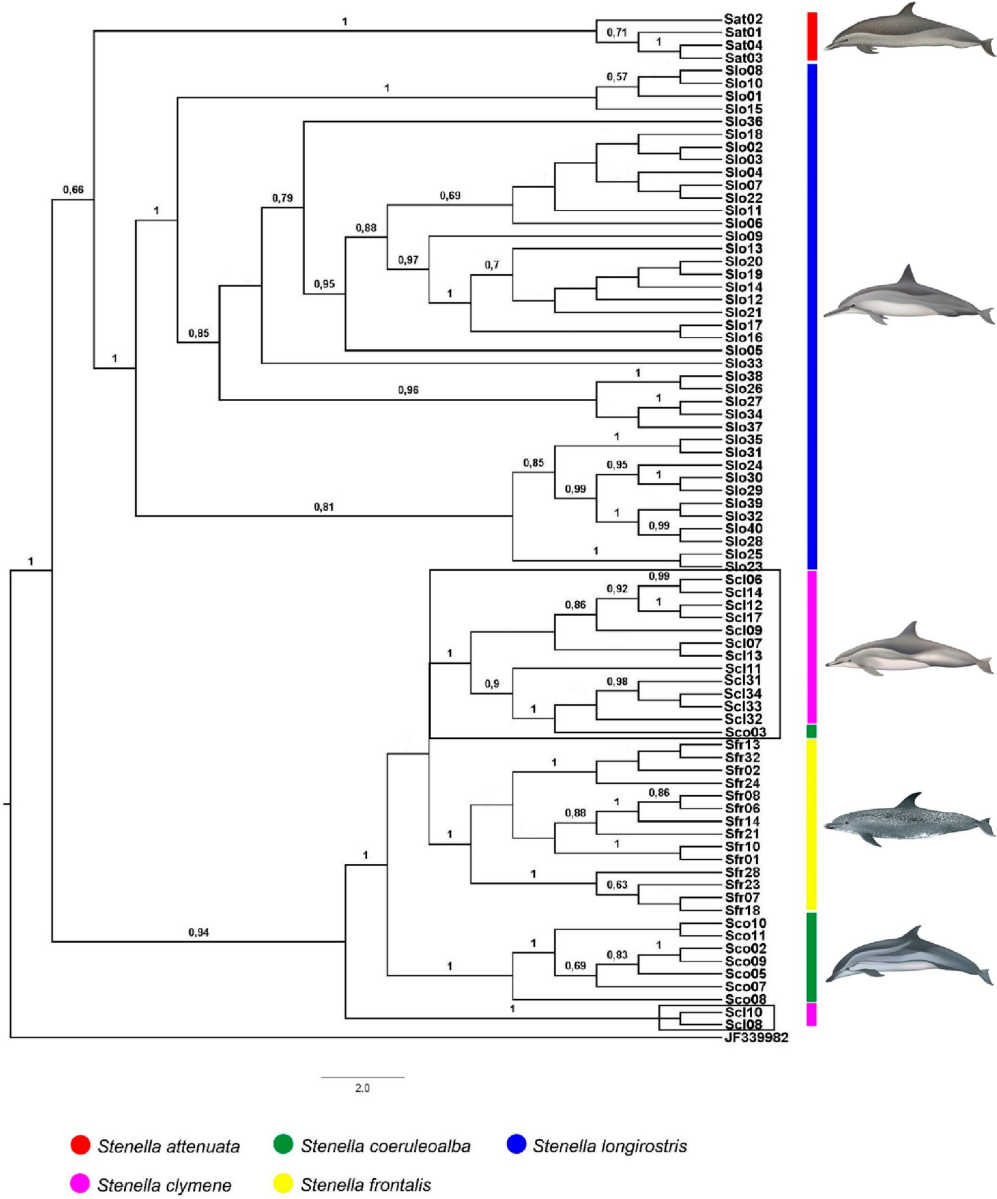
Bayesian cladogram generated by Beast of *Stenella* specimens from Brazilian waters for 1503 bp of mtDNA (Dloop + Cytb + CoxI) (see Tables 1 and 2). Posterior probability values greater than 0,5 are presented above nodes. Black boxes indicate misplaced specimens. Dolphins images have been extracted from the website http://cis.whoi.edu/science/B/whalesounds/index.cf.

The Bayesian phylogenetic cladogram combining all the three genes displayed the same resolution of the trees generated for the three genes separatlly so we decided to show the three genes cladograms separately. The best evolutionary model indicated by the Akaike Information Criteria (AIC) test implemented in the program jModeltest v2.1.6 was GTR+I+G [59] for all three genes. The cladograms of the three genes separately showed strong support for clades representing *S*. *attenuata*, *S*. *longirostris* and *S*. *frontalis* (posterior probability = 1) (Fig 3). As with the haplotype networks, however, three specimens were positioned in clades of species other than their morphological identification, i.e. individuals whose morphological identification did not match the presumed species of their respective mtDNA clade. One specimen of *S*. *coeruleoalba* (Sco03) was always placed in the *S*. *clymene* clade and two *S*. *clymene* specimens (Scl08 and Scl10) were always positioned outside the clades of other *S*. *clymene* and *S*. *coeruleoalba*. Cladograms of Dloop and Cytb analyzed individually showed the same pattern; the cladogram of CoxI also showed one *S*. *attenuata* specimen (Sat01) placed together with *S*. *clymene* specimens (S1–S3 Figs).

The three specimens with haplotypes positioned in clades of species different than their morphological identification correspond to stranded dolphins morphologically identified by experienced researchers or trained field correspondents. Unfortunately, voucher material is not available for all of them. The incongruity of the morphological identification and the mtDNA identity of these specimens was supported by searches of GenBank and the DNA Surveillance databases (S5 Table).

### Ocean basins comparisons

**Fig 4:**
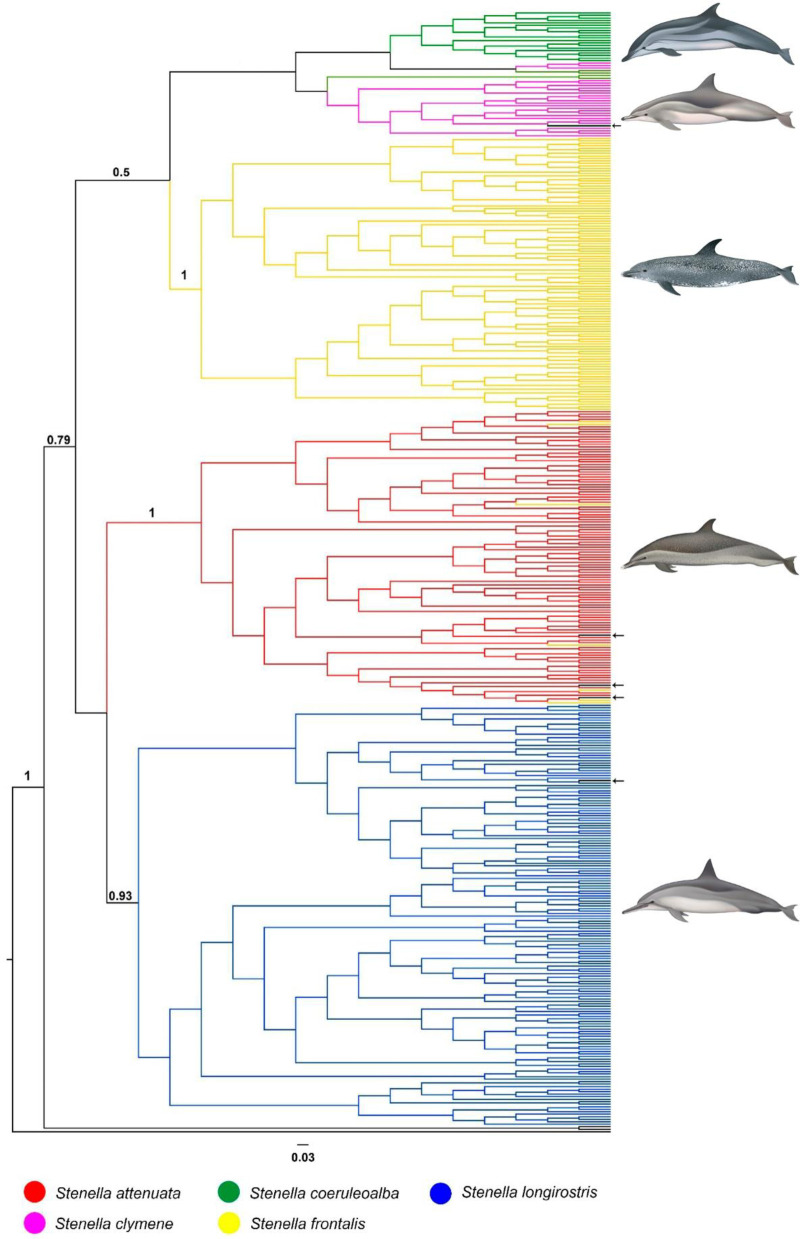
Bayesian cladogram generated by Beast of Dloop sequences (331bp) for *Stenella* for ocean basins comparisons. Posterior probability values greater than 0.5 are presented above nodes. Black arrows indicate misplaced haplotypes. Dolphins images have been extracted from the website http://cis.whoi.edu/science/B/whalesounds/index.cf.

**Fig 5:**
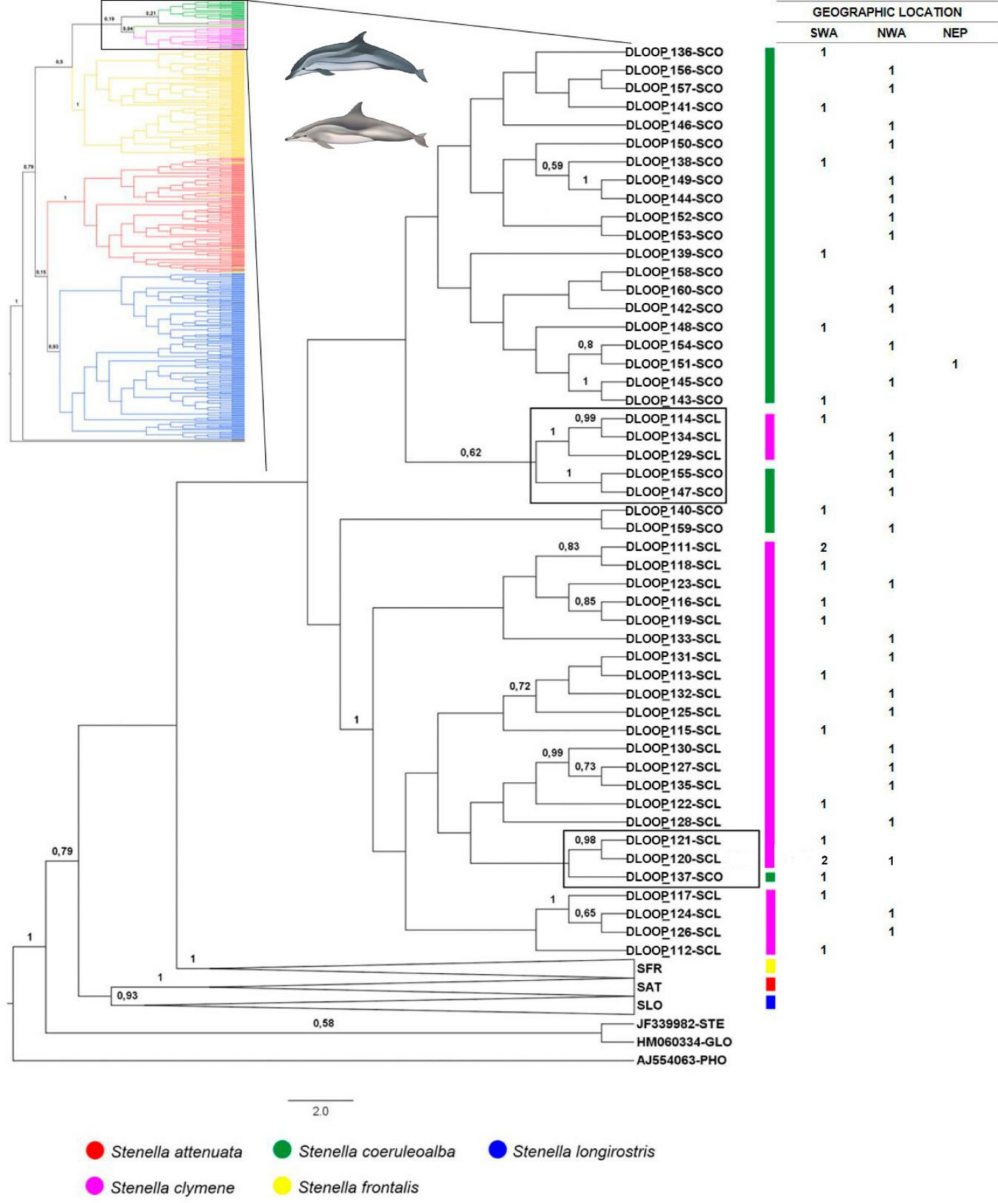
Bayesian cladogram generated by Beast from the Dloop sequences (331bp) highlighting the clade of *S*. *clymene* and *S*. *coeruleoalba* for ocean basin comparisons. Posterior probability values greater than 0.5 are presented above nodes. Black boxes indicate misplaced haplotypes. Asterisk indicate haplotypes presents in different species. Table at right displays the ocean basin location of each haplotype and the number of specimens: SWA (Southwest Atlantic Ocean), NWA (Northwest Atlantic Ocean), NEP (Northeast Pacific Ocean). The numbers in the columns represent the number of specimens for each haplotype. Dolphins images have been extracted from the website http://cis.whoi.edu/science/B/whalesounds/index.cf.

**Fig 6:**
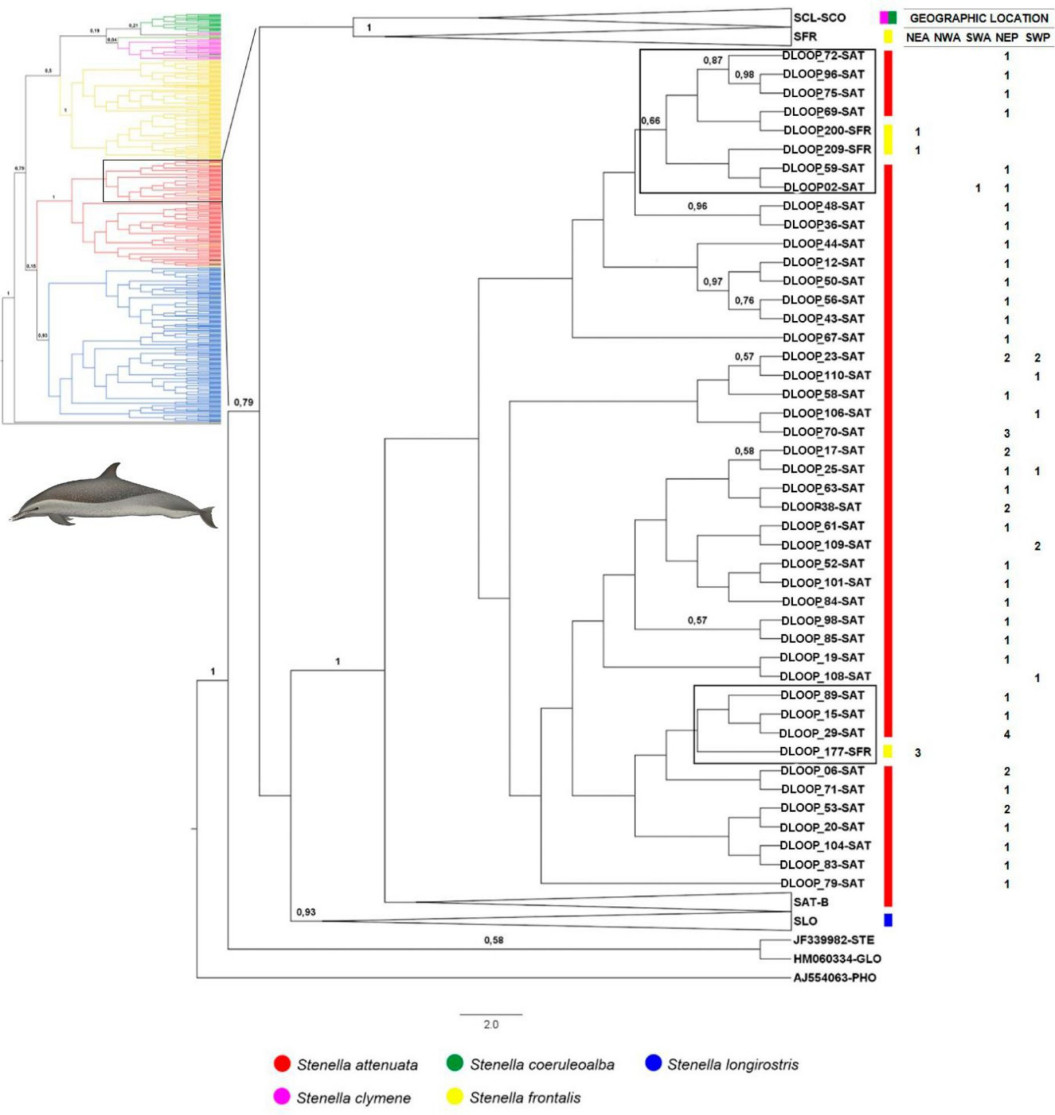
Bayesian cladogram generated by Beast from the Dloop sequences (331bp) highlighting the first clade of *Stenella attenuata* for ocean basin comparisons. Posterior probability values greater than 0.5 are presented above nodes. Black boxes indicate misplaced haplotypes. Table at right displays the ocean basin location of each haplotype and the number of specimens: NEA (Northeast Atlantic Ocean), NWA (Northwest Atlantic Ocean), SWA (Southwest Atlantic Ocean), NEP (Northeast Pacific Ocean), SWP (Southwest Pacific Ocean). The numbers in the columns represent the number of specimens for each haplotype. Dolphins images have been extracted from the website http://cis.whoi.edu/science/B/whalesounds/index.cf.

**Fig 7:**
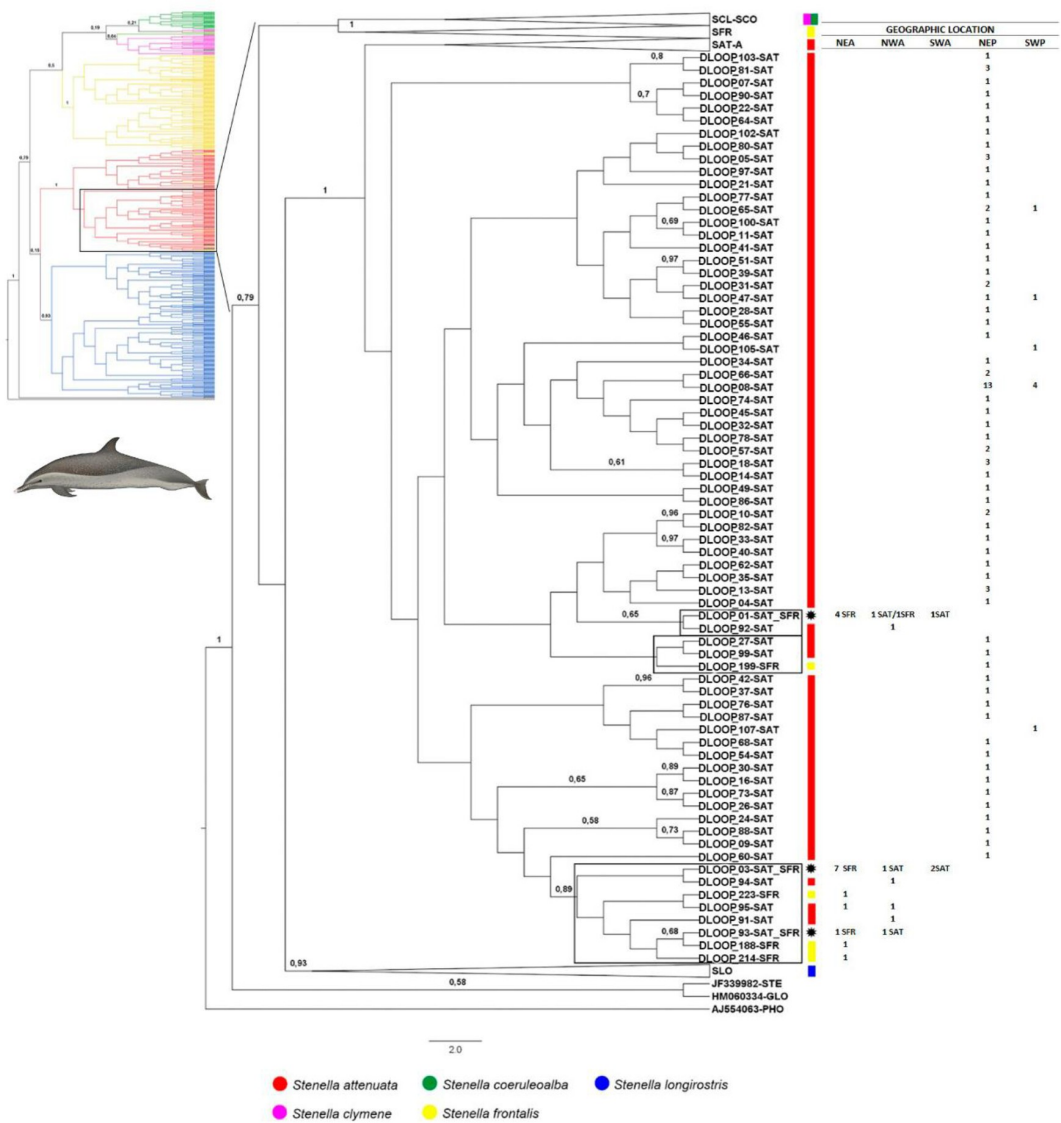
Bayesian cladogram generated in Beast from the Dloop sequences (331bp) highlighting the second clade of *Stenella attenuata* for ocean basin comparisons. Posterior probability values greater than 0.5 are presented above nodes. Black boxes indicate misplaced haplotypes. Table at right displays the ocean basin location of each haplotype and the number of specimens: NEA (Northeast Atlantic Ocean), NWA (Northwest Atlantic Ocean), SWA (Southwest Atlantic Ocean), NEP (Northeast Pacific Ocean), SWP (Southwest Pacific Ocean). The numbers in the columns represent the number of specimens for each haplotype. Dolphins images have been extracted from the website http://cis.whoi.edu/science/B/whalesounds/index.cf.

The Dloop analyses comprise the largest sample size providing 788 sequences, resolving 444 haplotypes. The best evolutionary model indicated by J-Modeltest was GTR+I+G. Well supported clades (posterior probability greater than 90) were identified for *S*. *attenuata*, *S*. *frontalis* and *S*. *longirostris* but not for *S*. *clymene* and *S*. *coeruleoalba* (Fig 4). No phylogeographic signal was detected among individuals of all species and from different ocean basin (Atlantic, Indian and Pacific) (Figs 5–7).

**Fig 8:**
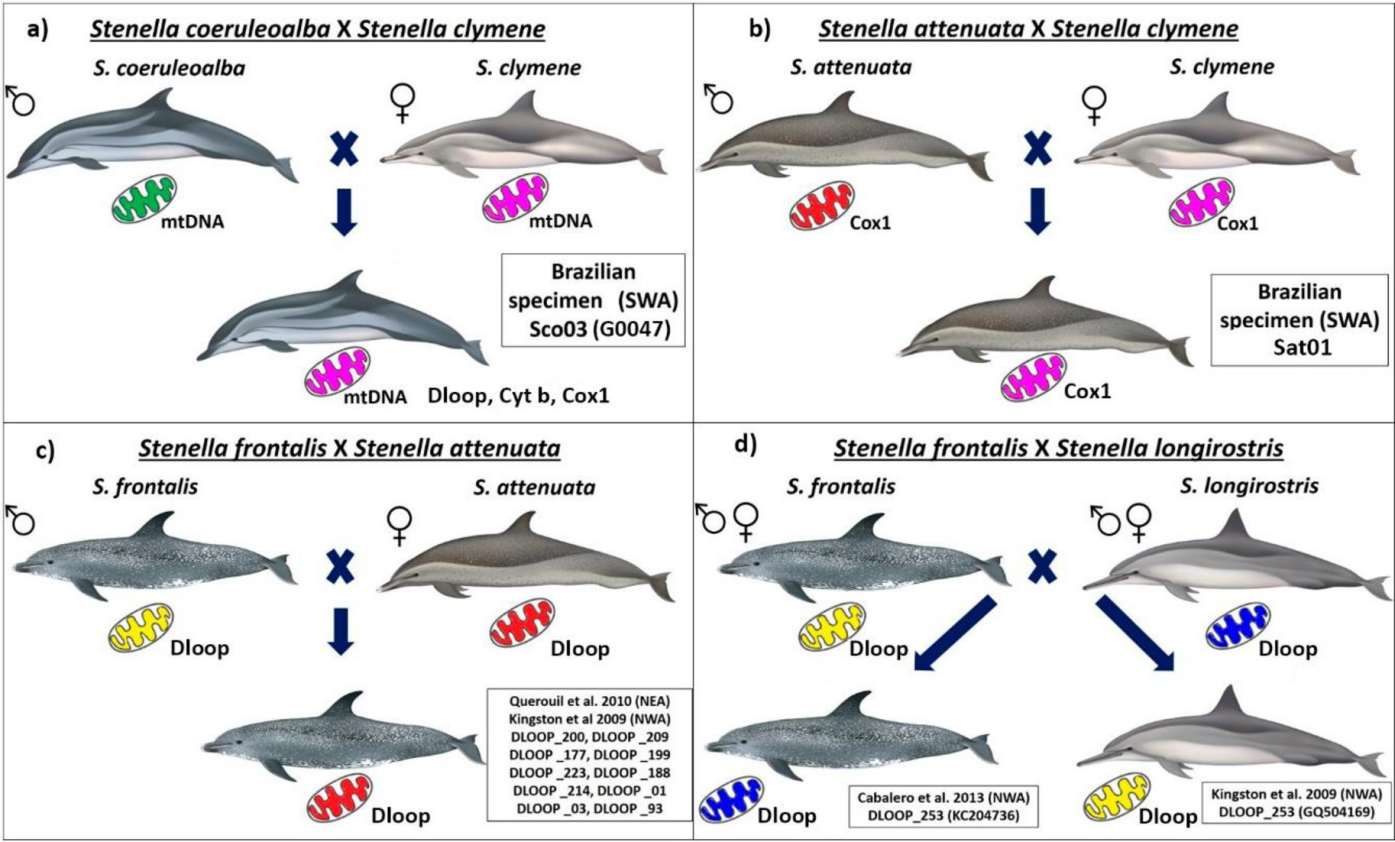
Possible heredograms of admixed individuals among *Stenella* species: a) between *S*. *coeruleoalba* and *S*. *clymene;* b) between *S*. *attenuata* and *S*. *clymene*; c) between *S*. *frontalis* and *S*. *attenuata*; d) between *S*. *frontalis* and *S*. *longirostris*. The images of the dolphins represent the morphological identity of the specimens, the colour of the mitochondrion represents the mitochondrial identity. In each panel the names of the possible hybrid specimens or the names of the haplotypes representing the possible hybrid specimens are listed. Dolphin images have been extracted from the website http://cis.whoi.edu/science/B/whalesounds/index.cf.

The clades representing *S*. *clymene* and *S*. *coeruleoalba* species showed some individuals positioned in species clades different than their morphological identification (Fig 5). DLOOP _137(Sco03) is from a specimen identified in the field as *S*. *coeruleoalba* that nested within a well-supported clade that contained the majority of *S*. *clymene* sequences within this clade. Three haplotypes of *S*. *clymene* (DLOOP_114, DLOOP_134, DLOOP_129) and two of *S*. *coeruleoalba* (DLOOP_155, DLOOP_147) were grouped in a clade with moderate support (posterior probability = 0.62). Of those DLOOP_155 and DLOOP_147 displayed Dloop identity as *S*. *clymene* on DNA Surveillance (Figs 5 and 8 and S5 Table).

*Stenella attenuata* sequences resolved into two well-supported clades, within which several haplotypes represented by specimens of *S*. *frontalis* were identified (Figs 4, 6 and 7). The majority of these *S*. *frontalis* haplotypes were from specimens sampled in the Northeast Atlantic Ocean (DLOOP_200, DLOOP_209, DLOOP_177, DLOOP_223, DLOOP_188, DLOOP_214), one was sampled in the Northeast Pacific Ocean (DLOOP_199) (Figs 6 and 7). All these sequences displayed Dloop identity as *S*. *attenuata* (Fig 8 and S5 Table). Three haplotypes, represented by specimens of two different species, *S*. *attenuata* and *S*. *frontalis*, were identified: DLOOP_01, DLOOP_03 and DLOOP_93 (Figs 7 and 8). The haplotype 01 (DLOOP_01) was shared by seven sequences, of those, five displayed Dloop identity as *S*. *attenuata* (morphologically identified as *S*. *frontalis*, GenBank records) and the other two as *S*. *frontalis* (morphologically identified as *S*. *frontalis*, GenBank records) (Figs 7 and 8 and S5 Table). The haplotype 03 (DLOOP_03) was shared by ten sequences, of those seven displayed Dloop identities as *S*. *attenuata* (morphologically identified as *S*. *frontalis*, GenBank records) and three as *S*. *frontalis* (morphologically identified as *S*. *attenuata*, GenBank records) (Figs 7 and 8 and S5 Table). The haplotype 93 (DLOOP_93) was represented by two sequences, one displayed Dloop identity as *S*. *attenuata* (morphologically identified as *S*. *attenuata*, GenBank records) and the other as *S*. *frontalis* (morphologically identified as *S*. *frontalis*, GenBank records) (Figs 7 and 8 and S5 Table).

In the *S*. *longirostris* clade it was possible to identify that DLOOP_ 253 was represented by sequences from the Northwest Atlantic Ocean of two different species: KC204736 whose morphological identity as *S*. *frontalis* (GenBank records) did not match with the Dloop identity of *S*. *longirostris;* and GQ504169 whose morphological identity as *S*. *longirostris* (GenBank records) did not match with the Dloop identity of *S*. *frontalis* (Figs 4 and 8 and S5 Table).

CoxI and Cytb cladograms also identified well-supported clades (posterior probabilities greater than 0.9) for *S*. *attenuata*, *S*. *frontalis* and *S*. *longirostris* but not for *S*. *clymene* and *S*. *coeruleoalba* (S4 and S5 Figs). The best evolutionary model indicated by J-Modeltest for CoxI was GTR+I+G and for Cytb was GTR+G. No phylogeographic signal was detected among individuals of all species and from different ocean basin (Atlantic, Indian and Pacific) (S4 and S5 Figs). One haplotype from each gene (CYTB_10 and COXI_15) nested within the *S*. *clymene* clade and were represented by specimens morphologically identified as both *S*. *coeruleoalba* (Sco03) and *S*. *clymene* (Scl31, Scl32, Scl33, Scl34) (S4 and S5 Figs and S3 Table). Although Cytb and CoxI analyses included fewer sequences they showed similar patterns of haplotypes positioned in clades of different species that did not correspond to their morphological identification according to GenBank records (see Supporting information figures and tables).

## Discussion

The results presented here provide new data on the genetic diversity of species of the genus *Stenella*. Despite the low sample size, with exception of *S*. *longirostris* (N = 40), the haplotype and nuclear diversity of *Stenella* species in the Southwest Atlantic Ocean were moderate to high, compared to those found in previous Dephinidae studies [1,3,60–63]. Though previous studies detecting genetic structuring for some *Stenella* species, especially for island-associated populations of *S*. *longirostris* [1,64,65] and for *S*. *clymene* in the Atlantic Ocean [2], no strong phylogeographic signal was detected at the ocean basin level for the *Stenella* species analysed here.

*S*. *clymene* and *S*. *coeruleoalba* demonstrated the highest haplotype diversity among the five species and showed the greatest evidence of lineage mixing, as did *S*. *clymene* and *S*. *attenuata* in the Southwest Atlantic Ocean (SWA). Moreover, potentially admixed individuals were identified between *S*. *frontalis* and *S*. *attenuata*, and between *S*. *frontalis* and *S*. *longirostris* in the Northwest Atlantic Ocean.

Generally, introgressive hybridization is only recognized in the wild when individuals exhibit morphological characteristics that are intermediate of the two parental species [64,66]. An example of this within the genus *Stenella*, was documented in the Fernando de Noronha archipelago where one individual presented morphological features of *S*. *longirostris* and *S*. *attenuata*; and another presented morphological features of *S*. *longirostris* and *S*. *clymene* [29]. Although intermediate morphology is strong evidence of hybridization, it should not be considered as a diagnostic. There is a possibility that hybrids (fertile cases) will backcross with one of the parental species and exhibit the dominant morphology of this species and, therefore, be "camouflaged" within these populations [67].

In this study the delimitation of species levels was based on the separation of lineages (i.e. monophyletic species concept). According to this concept, species are lineages that evolve separately from another lineage [68]. The evidence used for this purpose is a genealogical reciprocal monophyly and divergence among haplogroups. Three of the five species of *Stenella* (*S*. *attenuata*, *S*. *frontalis* and *S*. *longirostris*) exhibited clades well supported in all analyses, for Cytb and CoxI genes, supporting their recognition as distinct species by the above definition. *Stenella coeruleoalba* and *S*. *clymene*, on the other hand, showed high levels of genetic diversity and also mixed clades in all three regions of the mitochondrial DNA evaluated, as previously described in other phylogenetic studies using different molecular markers [4,6,27].

Within the Brazilian waters we found one individual (Sco03, GEMARS 0047) which displayed morphological identity consistent with *S*. *coeruleoalba*, but a genetic (mDNA) identity consistent with *S*. *clymene* for all three genes evaluated (Dloop, Cytb and CoxI), this was shown by both the phylogenetic trees. It is worth mentioning that this stranded specimen (GEMARS 0047; Sco03 in this study) was originally identified by cetacean specialists and the identification was based on many features (coloration, external morphology and osteological characters) that correspond to *S*. *coeruleoalba* [69]. This original morphological identification was further confirmed in this study by the reexamination of the voucher material (GEMARS 0047, skull and photographs) of the specimen (a male 227.5 cm in length), which had the typical diagnostic lateral stripes (eye-to-flipper and eye-to-anus) of *S*. *coeruleoalba* [70] (S6 Table and S6 Fig). In cases such as this, where the genetic identity is different to the original morphological description, it is important to have the genetic samples linked to voucher material in scientific collections to enable reexamination and to certify morphological identification.

Although *S*. *clymene* was considered to have arisen through natural hybridization between *S*. *longirostris* and *S*. *coeruleoalba*, backcrosses may still occur [26]. However, in this study we only find a sign of mixture between *S*. *clymene* and *S*. *coeruleoalba*. Two specimens morphologically identified as *S*. *clymene* (Scl08 and Scl10, S7 Fig) were consistently positioned outside the *S*. *clymene* clades, but did not show any signs of mixture with other *Stenella* species.

We also found possible evidence of introgressive hybridization between *S*. *attenuata* and *S*. *clymene* (Sat01 specimen) in Brazilian waters revealed exclusively by the results of CoxI. The CoxI gene has been widely used in the molecular identification of species through the *Barcode* DNA methodology [71] and has been efficient, in most cases studied, at correctly identifying organisms [11]. For cetaceans, CoxI has also proved to be sufficient to distinguish most species. However, CoxI phylogenetic trees are often not able to separate species of closely related taxa or of taxonomic groups that are not well resolved, such as the case of delphinids [53,39,71]. Therefore, considering the close relationship among the species of the genus *Stenella* and the potential limitations of the CoxI for discrimination of closely related taxa, this possible case of mixture between *S*. *attenuata* and *S*. *clymene* should be viewed with caution.

*Stenella attenuata* and *S*. *clymene* are known to demonstrate similar environmental constraints and are thought to have a wide degree of niche overlap in the SWA and the Gulf of Mexico [72–74]. Furthermore, although underwater photographs taken from free-swimming dolphins in the Fernando de Noronha Archipelago suggest the occurrence of two possible hybrids, one between *S*. *longirostris* and *S*. *attenuata* and another between *S*. *longirostris* and *S*. *clymene*, [29], we did not find any signs of hybridization among these species in our genetic analyses.

In addition to the possible admixed specimens in Brazilian waters, the inclusion of the published Dloop sequences provided evidence of admixed individuals in the Northeast Atlantic Ocean between *S*. *frontalis* and *S*. *attenuata* and between *S*. *frontalis* and *S*. *longirostris*. All sequences used were from specimens morphologically identified and certified by the authors of the papers published in peer-reviewed scientific journals. All these sequences that were positioned in different species clades presented Dloop identity inconsistent with the morphological identity reported in the original papers [6].

All the admixed specimens were identified in areas where both parental species occur with at least some contact between them. In addition, mixed groups have been occasionally observed for some *Stenella* species, for example between pantropical spotted (*S*. *attenuata*) and spinner dolphin (*S*. *longirostris*) in the eastern tropical Pacific [75–78] in Hawaiian waters [79], and along the Brazilian continental shelf and offshore waters [15,74].

We suggest the existence of introgression among some maternal lineages of *Stenella* species as a result of hybridization in the past among different species of this genus. Our results support previous evidence that this phenomenon is a more common evolutionary process in *Stenella* than previously thought. Hybridization has been in fact indicated as one of the possible explanations for the complex taxonomical history and long debate about the phylogenetic relations in delphinids [5–10,14,80]. A large number of mtDNA sequences of *Stenella*, including specimens from all oceans were used to demonstrate that introgressive hybridization is occurring among *Stenella* dolphins. To improve our results and better assess the level of introgression existing between these species it is important to enhance the number of sequences of all *Stenella* species from the Southwest Atlantic Ocean, with the exception of *S*. *longirostris*, and analyse nuclear markers or the complete genome of these specimens in addition of the three mitochondrial genes used in the present study. Moreover, our study also highlights the importance of having a genetic sample accompanied by a voucher material in scientific collections.

## Conclusion

Our study brings new data on genetic diversity and phylogeny of *Stenella* genus and possible past hybridization as explanation for genetic mixture within this species in the South Atlantic Ocean. A large number of mtDNA sequences of *Stenella* including specimens from all oceans were used to support previous evidence that introgressive hybridization is occurring among *Stenella* dolphins, based on morphology and on mtDNA data. Moreover, our study also reinforces the importance of having a genetic sample accompanied by a voucher material in scientific collections.

Finally, the genetic information gathered here is also important in a conservation perspective. Three of the five species (*S*. *attenuata*, *S*. *coeruleoalba* and *S*. *longirostris*) are considered to be of “Least Concerned” by the World Conservation Union [81], and two of them (*S*. *clymene* and *S*. *frontalis*) are considered “Data Deficient”. For all these species, information on their biology, ecology, genetic diversity and evolutionary history are necessary for the implementation of adequate conservation and management strategies [82].

## Supporting information

S1 Fig Bayesian cladogram generated by Beast for Dloop marker in Southwest Atlantic Ocean for *Stenella* specimens. Posterior probability values greater than 0.5 are presented above nodes. Black boxes indicate specimen’s haplotypes positioned in clades of species different than their morphological identification. Dolphin images have been extracted from the website http://cis.whoi.edu/science/B/whalesounds/index.cf.
(TIF)

S2 Fig Bayesian cladogram generated by Beast for the Cytb gene in Southwest Atlantic Ocean for *Stenella* specimens. Posterior probability values greater than 0,5 are presented above nodes. Black boxes indicate specimen’s haplotypes positioned in clades of species different than their morphological identification. Dolphin images have been extracted from the website http://cis.whoi.edu/science/B/whalesounds/index.cf.
(TIF)

S3 Fig Bayesian cladogram generated by Beast for the Cox 1 gene in Southwest Atlantic Ocean for *Stenella* specimens. Posterior probability values greater than 0,5 are presented above nodes. Black boxes indicate specimen’s haplotypes positioned in clades of species different than their morphological identification. Dolphin images have been extracted from the website http://cis.whoi.edu/science/B/whalesounds/index.cf.
(TIF)

S4 Fig Bayesian cladogram generated by Beast of 83 haplotypes (170 sequences) from Cytb gene for *Stenella* for ocean basins comparisons, 331bp. AIC evolutionary model: GTR+G. Posterior probability values greater than 0.5 are presented above nodes. Black boxes indicate haplotypes positioned in clades of species different than their morphological identification. Asterisk indicate haplotypes present in different species. Table at right display the ocean basin location of each haplotype marked by “X”: NEA (Northeast Atlantic Ocean, NWA (Northwest Atlantic Ocean), SWA (Southwest Atlantic Ocean), NEP (Northeast Pacific Ocean), NWP (Northwest Pacific Ocean), IN (Indian Ocean), SEP (Southeast Pacific Ocean), SWP (Southwest Pacific Ocean). The numbers in the SWA column represent the number of specimens for each haplotype. Dolphin images have been extracted from the website http://cis.whoi.edu/science/B/whalesounds/index.cf.
(TIF)

S5 Fig Bayesian cladogram generated by Beast of 57 haplotypes (111 sequence) from Cox1 gene for *Stenella* for ocean basins comparisons, 613 bp. AIC evolutionary model: GTR+I+G. Posterior probability values greater than 0.5 are presented above nodes. Black boxes indicate haplotypes positioned in clades of species different than their morphological identification. Asterisk indicate haplotypes present in different species Table at right display the ocean basin location of each haplotype marked by “X”: NEA (Northeast Atlantic Ocean, NWA (Northwest Atlantic Ocean), SWA (Southwest Atlantic Ocean), NEP (Northeast Pacific Ocean), NWP (Northwest Pacific Ocean). The numbers in the SWA column represent the number of specimens for each haplotype. Dolphin images have been extracted from the website http://cis.whoi.edu/science/B/whalesounds/index.cf.
(TIF)

S6 Fig Picture of the specimen Sco03 (GEMARS 0047) morphologically identified as *Stenella coeruleoalba* (Ott and Danilewicz, 1996), but as a putative hybrid between *S*. *coeruleoalba* and *S*. *clymene* by molecular markers. (Photo: Rodrigo Baleia/GEMARS).
(TIF)

S7 Fig Pictures of the specimen Scl10 (AQUASIS 02C1151/476) morphologically identified as *Stenella clymene*, positioned outside the clades of *S*. *clymene* in all analyses. (Photos: AQUASIS).
(TIF)

S1 Table Number of specimens of each species of *Stenella* analysed in this study, including geographic location and sampling method. The species were originally identified based on morphological traits.
(DOCX)

S2 Table Genbank sequences used in this study. Name of the species, GenBank accession number, name of the haplotype used in this study, geographic location of the haplotypes and source. NEA (Northeast Atlantic Ocean), NWA (Northwest Atlantic Ocean), SWA (Southwest Atlantic Ocean), NEP (Northeast Pacific Ocean), NWP (Northwest Pacific Ocean), SWP (Southwest Pacific Ocean), SEP (Southeast Pacific Ocean), EP (East Pacific Ocean), EA (East Atlantic Ocean), IN (Indian Ocean), SWI (Southwest Indian Ocean), NEI (Northeast Indian Ocean), IP (Indo Pacific Ocean).
(DOCX)

S3 Table Genetic diversity values of *Stenella* species from Brazilian waters for mtDNA control region (Dloop), cytochrome b (Cyt b), and cytochrome oxidase subunit I (Cox I). Sample size (N), number of haplotypes (Nh), polymorphic sites (Ps), haplotype diversity (h), and nucleotide diversity (π). Bp means base pair length.
(DOCX)

S4 Table Genetic distances between *Stenella* species from Brazilian waters for mtDNA control region (Dloop), cytochrome b (Cyt b), and cytochrome oxidase subunit I (Cox I). Genetic distance values (%) are bellow diagonal, and, standard errors (SEs) are upper diagonal.
(DOCX)

S5 Table Blasts of GenBank and DNA Surveillance for haplotypes positioned in clades of species different than their morphological identification and haplotypes represented by sequences of different species. (DOCX)

S6 Table Skull measurements and meristics (following Perrin, 1975) of the specimen morphologically identified as *Stenella coeruleoalba* (GEMARS 0047, Sco03 in this study). (DOCX)

S7 Table External measures (according to Perrin, 1975) of the specimen AQUASIS 02C1151/476 (Scl 10 in this study), morphologically identified as *Stenella clymene*. (DOCX)
