# Supplementary figures and images for: Mitochondrial diversity and inter-specific phylogeny among dolphins of the genus Stenella in the Southwest Atlantic Ocean
Source: PLoS One. 2022 Jul 14;17(7):e0270690. doi: 10.1371/journal.pone.0270690 (PMC9282552; doi:10.1371/journal.pone.0270690)

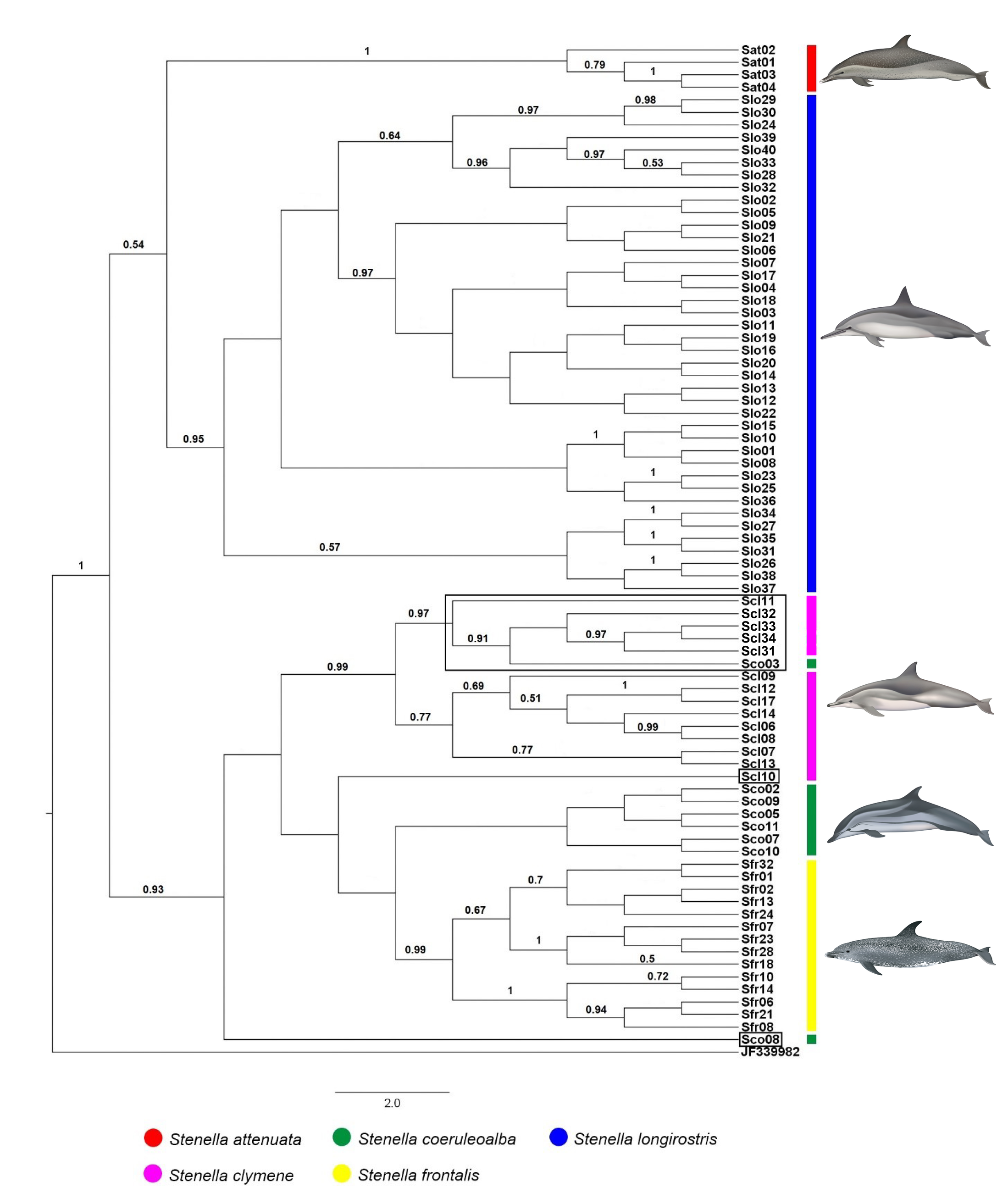

Supplement: S1 Fig — Posterior probability values greater than 0.5 are presented above nodes. Black boxes indicate specimen’s haplotypes positioned in clades of species different than their morphological identification. Dolphin images have been extracted from the website http://cis.whoi.edu/science/B/whalesounds/index.cf. (TIF) [file pone.0270690.s001.tif]

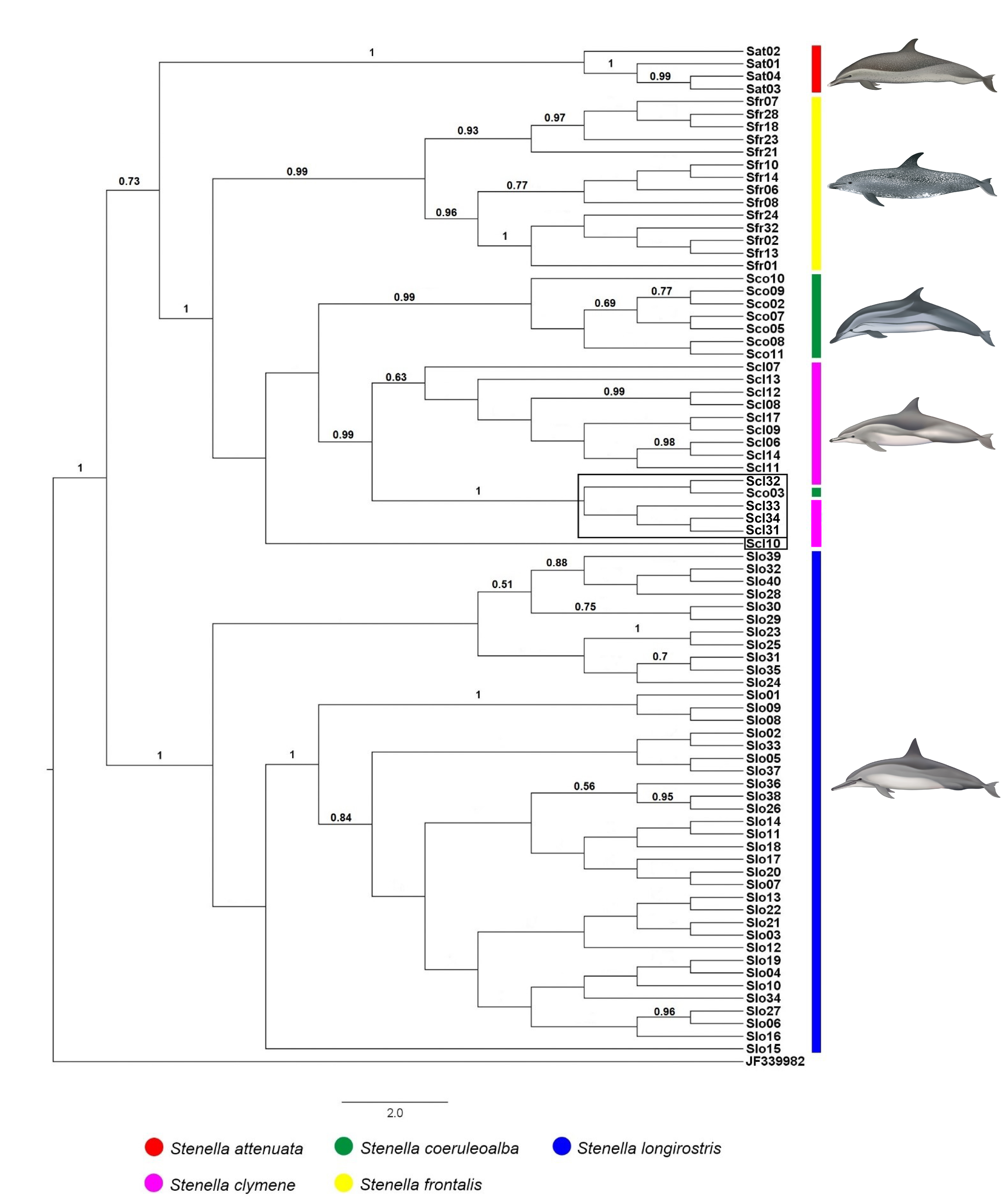

Supplement: S2 Fig — Posterior probability values greater than 0,5 are presented above nodes. Black boxes indicate specimen’s haplotypes positioned in clades of species different than their morphological identification. Dolphin images have been extracted from the website http://cis.whoi.edu/science/B/whalesounds/index.cf. (TIF) [file pone.0270690.s002.tif]

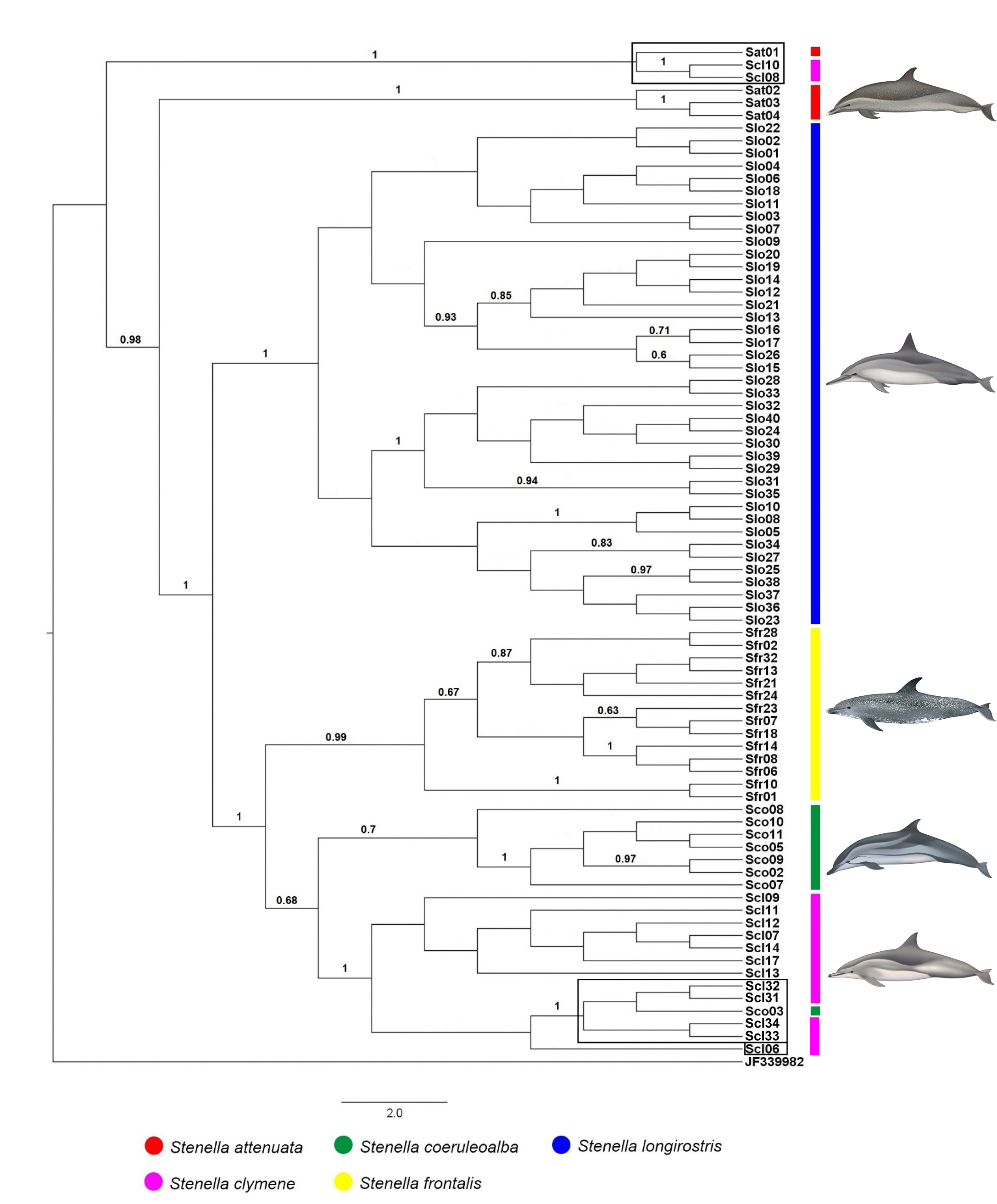

Supplement: S3 Fig — Posterior probability values greater than 0,5 are presented above nodes. Black boxes indicate specimen’s haplotypes positioned in clades of species different than their morphological identification. Dolphin images have been extracted from the website http://cis.whoi.edu/science/B/whalesounds/index.cf. (TIF) [file pone.0270690.s003.tif]

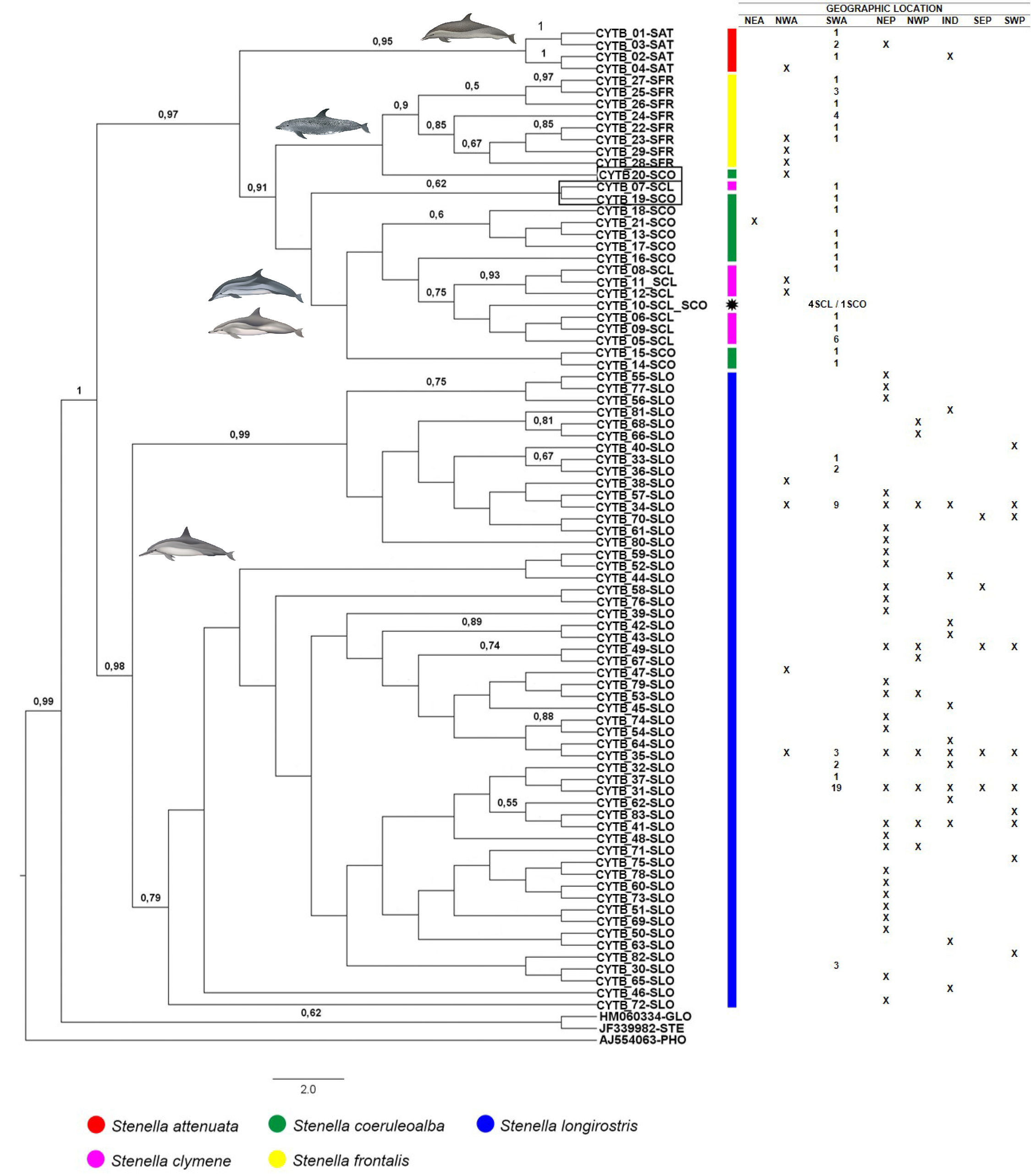

Supplement: S4 Fig — AIC evolutionary model: GTR+G. Posterior probability values greater than 0.5 are presented above nodes. Black boxes indicate haplotypes positioned in clades of species different than their morphological identification. Asterisk indicate haplotypes present in different species. Table at right display the ocean basin location of each haplotype marked by “X”: NEA (Northeast Atlantic Ocean, NWA (Northwest Atlantic Ocean), SWA (Southwest Atlantic Ocean), NEP (Northeast Pacific Ocean), NWP (Northwest Pacific Ocean), IN (Indian Ocean), SEP (Southeast Pacific Ocean), SWP (Southwest Pacific Ocean). The numbers in the SWA column represent the number of specimens for each haplotype. Dolphin images have been extracted from the website http://cis.whoi.edu/science/B/whalesounds/index.cf. (TIF) [file pone.0270690.s004.tif]

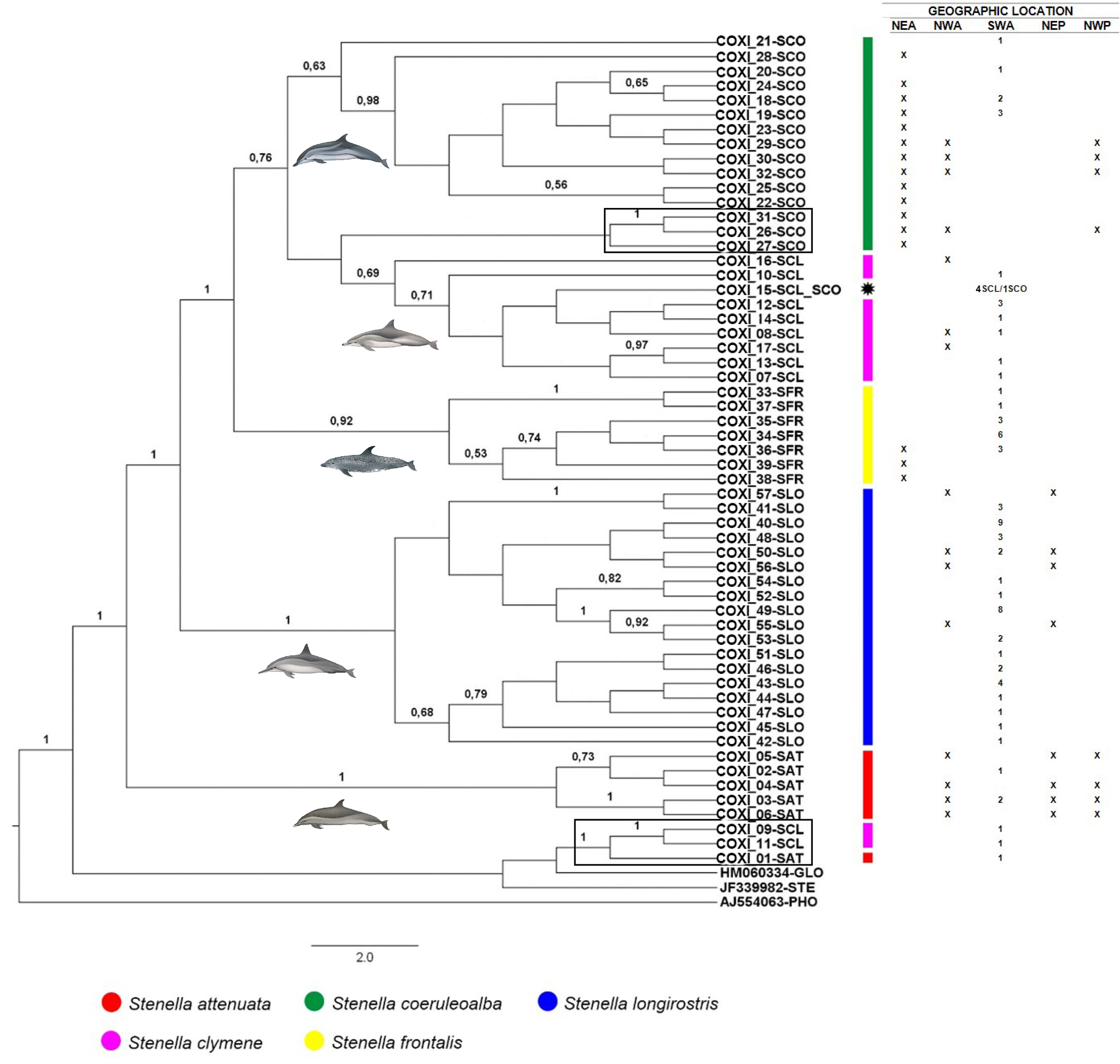

Supplement: S5 Fig — AIC evolutionary model: GTR+I+G. Posterior probability values greater than 0.5 are presented above nodes. Black boxes indicate haplotypes positioned in clades of species different than their morphological identification. Asterisk indicate haplotypes present in different species Table at right display the ocean basin location of each haplotype marked by “X”: NEA (Northeast Atlantic Ocean, NWA (Northwest Atlantic Ocean), SWA (Southwest Atlantic Ocean), NEP (Northeast Pacific Ocean), NWP (Northwest Pacific Ocean). The numbers in the SWA column represent the number of specimens for each haplotype. Dolphin images have been extracted from the website http://cis.whoi.edu/science/B/whalesounds/index.cf. (TIF) [file pone.0270690.s005.tif]

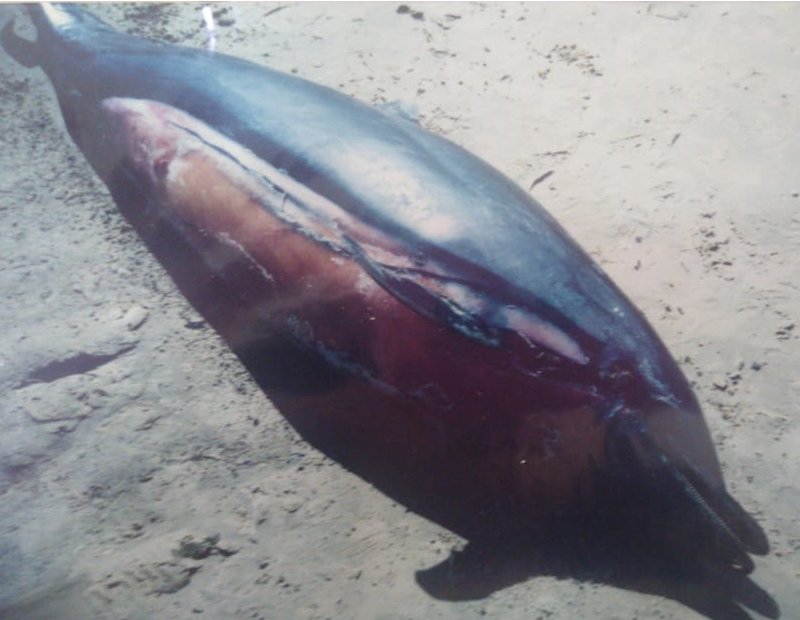

Supplement: S6 Fig — (Photo: Rodrigo Baleia/GEMARS). (TIF) [file pone.0270690.s006.tif]

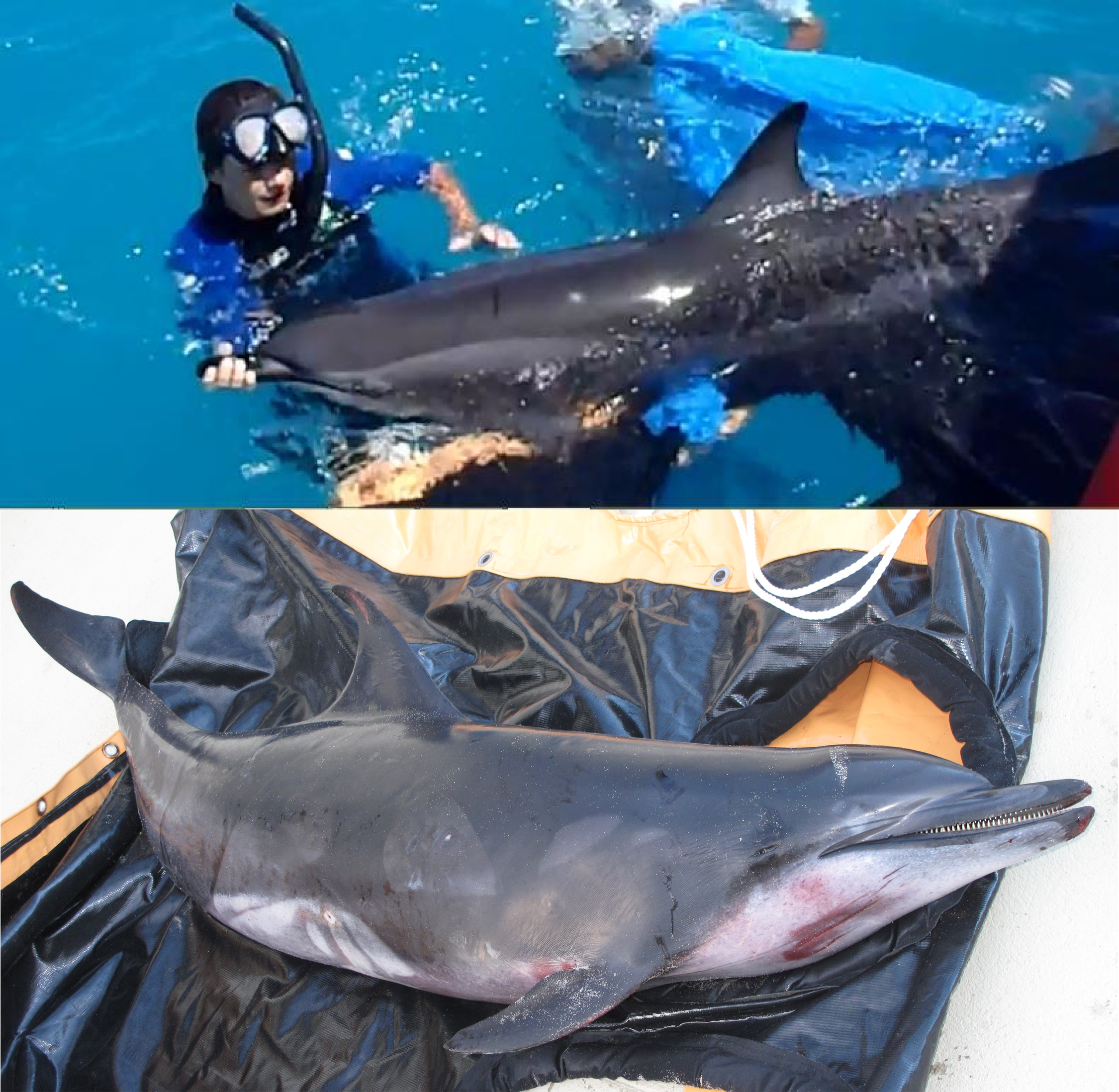

Supplement: S7 Fig — (Photos: AQUASIS). (TIF) [file pone.0270690.s007.tif]
