# Supplementary material for: Mitochondrial diversity and inter-specific phylogeny among dolphins of the genus Stenella in the Southwest Atlantic Ocean
Source: PLoS One. 2022 Jul 14;17(7):e0270690. doi: 10.1371/journal.pone.0270690 (PMC9282552; doi:10.1371/journal.pone.0270690)
Supplement: S1 Table — The species were originally identified based on morphological traits. (DOCX) [file pone.0270690.s008.docx]

**S1** **Table Number of specimens of each species of *Stenella* analysed in this study, including geographic location and sampling method. The species were originally identified based on morphological traits.**

|  | **Sample** | **Species** | **Localization** | **Sampling Method** |
| --- | --- | --- | --- | --- |
| 1 | Sat 01 | *Stenella attenuata* | Fernando de Noronha/PE | Stranding |
| 2 | Sat 02 | *Stenella attenuata* | Fernando de Noronha/PE | Stranding |
| 3 | Sat 03 | *Stenella attenuata* | Rio de Janeiro (RJ) | Biopsy |
| 4 | Sat 04 | *Stenella attenauta* | Ceará (CE) | Stranding |
| 5 | Scl 06 | *Stenella clymene* | Bahia (BA) | Stranding |
| 6 | Scl 07 | *Stenella clymene* | Bahia (BA) | Stranding |
| 7 | Scl 08 | *Stenella clymene* | Bahia (BA) | Stranding |
| 8 | Scl 09 | *Stenella clymene* | Ceará (CE) | Stranding |
| 9 | Scl 10 | *Stenella clymene* | Ceará (CE) | Stranding |
| 10 | Scl 11 | *Stenella clymene* | Ceará (CE) | Stranding |
| 11 | Scl 12 | *Stenella clymene* | Ceará (CE) | Stranding |
| 12 | Scl 13 | *Stenella clymene* | Ceará (CE) | Stranding |
| 13 | Scl 14 | *Stenella clymene* | Ceará (CE) | Stranding |
| 14 | Scl 16 | *Stenella clymene* | Fernando de Noronha/PE | Stranding |
| 15 | Scl 31 | *Stenella clymene* | Fernando de Noronha/PE | Stranding |
| 16 | Scl 32 | *Stenella clymene* | Fernando de Noronha/PE | Stranding |
| 17 | Scl 33 | *Stenella clymene* | Fernando de Noronha/PE | Stranding |
| 18 | Scl 34 | *Stenella clymene* | Fernando de Noronha/PE | Stranding |
| 19 | Sco 02 | *Stenella coeruleoalba* | Ceará (CE) | Stranding |
| 20 | Sco 03 (G0047) | *Stenella coeruleoalba* | Rio Grande do Sul (RS) | Stranding |
| 21 | Sco 05 | *Stenella coeruleoalba* | Rio Grande do Sul (RS) | Stranding |
| 22 | Sco 07 | *Stenella coeruleoalba* | Rio Grande do Sul (RS) | Stranding |
| 23 | Sco 08 | *Stenella coeruleoalba* | Rio Grande do Sul (RS) | Stranding |
| 24 | Sco 09 | *Stenella coeruleoalba* | Rio Grande do Sul (RS) | Stranding |
| 25 | Sco 10 | *Stenella coeruleoalba* | Rio Grande do Sul (RS) | Stranding |
| 26 | Sco 11 | *Stenella coeruleoalba* | Rio Grande do Sul (RS) | Stranding |
| 27 | Sfr 01 | *Stenella frontalis* | Fernando de Noronha (FN) | Stranding |
| 28 | Sfr 02 | *Stenella frontalis* | Fernando de Noronha (FN) | Stranding |
| 29 | Sfr 06 | *Stenella frontalis* | São Paulo (SP) | Biopsy |
| 30 | Sfr 07 | *Stenella frontalis* | São Paulo (SP) | Biopsy |
| 31 | Sfr 08 | *Stenella frontalis* | São Paulo (SP) | Biopsy |
| 32 | Sfr 10 | *Stenella frontalis* | São Paulo (SP) | Biopsy |
| 33 | Sfr 13 | *Stenella frontalis* | São Paulo (SP) | Biopsy |
| 34 | Sfr 14 | *Stenella frontalis* | São Paulo (SP) | Biopsy |
| 35 | Sfr 18 | *Stenella frontalis* | São Paulo (SP) | Biopsy |
| 36 | Sfr 21 | *Stenella frontalis* | São Paulo (SP) | Biopsy |
| 37 | Sfr 23 | *Stenella frontalis* | São Paulo (SP) | Biopsy |
| 38 | Sfr 24 | *Stenella frontalis* | São Paulo (SP) | Biopsy |
| 39 | Sfr 28 | *Stenella frontalis* | Rio Grande do Sul (RS) | Stranding |
|  | **Sample** | **Species** | **Localization** | **Sampling Method** |
| 40 | Sfr 32 | *Stenella frontalis* | Rio Grande do Sul (RS) | Stranding |
| 41 | Slo 01 | *Stenella longirostris* | Fernando de Noronha (FN) | Skin swabbing |
| 42 | Slo 02 | *Stenella longirostris* | Fernando de Noronha (FN) | Skin swabbing |
| 43 | Slo 03 | *Stenella longirostris* | Fernando de Noronha (FN) | Skin swabbing |
| 44 | Slo 04 | *Stenella longirostris* | Fernando de Noronha (FN) | Skin swabbing |
| 45 | Slo 05 | *Stenella longirostris* | Fernando de Noronha (FN) | Skin swabbing |
| 46 | Slo 06 | *Stenella longirostris* | Fernando de Noronha (FN) | Skin swabbing |
| 47 | Slo 07 | *Stenella longirostris* | Fernando de Noronha (FN) | Skin swabbing |
| 48 | Slo 08 | *Stenella longirostris* | Fernando de Noronha (FN) | Skin swabbing |
| 49 | Slo 09 | *Stenella longirostris* | Fernando de Noronha (FN) | Skin swabbing |
| 50 | Slo 10 | *Stenella longirostris* | Fernando de Noronha (FN) | Skin swabbing |
| 51 | Slo 11 | *Stenella longirostris* | Fernando de Noronha (FN) | Skin swabbing |
| 52 | Slo 12 | *Stenella longirostris* | Fernando de Noronha (FN) | Skin swabbing |
| 53 | Slo 13 | *Stenella longirostris* | Fernando de Noronha (FN) | Skin swabbing |
| 54 | Slo 14 | *Stenella longirostris* | Fernando de Noronha (FN) | Skin swabbing |
| 55 | Slo 15 | *Stenella longirostris* | Fernando de Noronha (FN) | Skin swabbing |
| 56 | Slo 16 | *Stenella longirostris* | Fernando de Noronha (FN) | Skin swabbing |
| 57 | Slo 17 | *Stenella longirostris* | Fernando de Noronha (FN) | Skin swabbing |
| 58 | Slo 18 | *Stenella longirostris* | Fernando de Noronha (FN) | Skin swabbing |
| 59 | Slo 19 | *Stenella longirostris* | Fernando de Noronha (FN) | Skin swabbing |
| 60 | Slo 20 | *Stenella longirostris* | Fernando de Noronha (FN) | Skin swabbing |
| 61 | Slo 21 | *Stenella longirostris* | Fernando de Noronha (FN) | Skin swabbing |
| 62 | Slo 22 | *Stenella longirostris* | Rio Grande do Norte (RN) | Stranding |
| 63 | Slo 23 | *Stenella longirostris* | Ceará (CE) | Stranding |
| 64 | Slo 24 | *Stenella longirostris* | Ceará (CE) | Stranding |
| 65 | Slo 25 | *Stenella longirostris* | Pernambuco (PE) | Stranding |
| 66 | Slo 26 | *Stenella longirostris* | Pernambuco (PE) | Stranding |
| 67 | Slo 27 | *Stenella longirostris* | Espírito Santo (ES) | Stranding |
| 68 | Slo 28 | *Stenella longirostris* | Rio de Janeiro (RJ) | Biopsy |
| 69 | Slo 29 | *Stenella longirostris* | Rio de Janeiro (RJ) | Biopsy |
| 70 | Slo 30 | *Stenella longirostris* | Rio de Janeiro (RJ) | Biopsy |
| 71 | Slo 31 | *Stenella longirostris* | Rio de Janeiro (RJ) | Biopsy |
| 72 | Slo 32 | *Stenella longirostris* | Rio de Janeiro (RJ) | Biopsy |
| 73 | Slo 33 | *Stenella longirostris* | Rio de Janeiro (RJ) | Biopsy |
| 74 | Slo 34 | *Stenella longirostris* | São Paulo (SP) | Biopsy |
| 75 | Slo 35 | *Stenella longirostris* | Paraná (PR) | Biopsy |
| 76 | Slo 36 | *Stenella longirostris* | Paraná (PR) | Biopsy |
| 77 | Slo 37 | *Stenella longirostris* | Paraná (PR) | Biopsy |
| 78 | Slo 38 | *Stenella longirostris* | Santa Catarina (SC) | Biopsy |
| 79 | Slo 39 | *Stenella longirostris* | Santa Catarina (SC) | Biopsy |
| 80 | Slo 40 | *Stenella longirostris* | Santa Catarina (SC) | Biopsy |
