# Supplementary material for: Mitochondrial diversity and inter-specific phylogeny among dolphins of the genus Stenella in the Southwest Atlantic Ocean
Source: PLoS One. 2022 Jul 14;17(7):e0270690. doi: 10.1371/journal.pone.0270690 (PMC9282552; doi:10.1371/journal.pone.0270690)
Supplement: S2 Table — Name of the species, GenBank accession number, name of the haplotype used in this study, geographic location of the haplotypes and source. NEA (Northeast Atlantic Ocean), NWA (Northwest Atlantic Ocean), SWA (Southwest Atlantic Ocean), NEP (Northeast Pacific Ocean), NWP (Northwest Pacific Ocean), SWP (Southwest Pacific Ocean), SEP (Southeast Pacific Ocean), EP (East Pacific Ocean), EA (East Atlantic Ocean), IN (Indian Ocean), SWI (Southwest Indian Ocean), NEI (Northeast Indian Ocean), IP (Indo Pacific Ocean). (DOCX) [file pone.0270690.s009.docx]

**S2 Table Genbank sequences used in this study.** Name of the species, GenBank accession number, name of the haplotype used in this study, geographic location of the haplotypes and source. NEA (Northeast Atlantic Ocean), NWA (Northwest Atlantic Ocean), SWA (Southwest Atlantic Ocean), NEP (Northeast Pacific Ocean), NWP (Northwest Pacific Ocean), SWP (Southwest Pacific Ocean), SEP (Southeast Pacific Ocean), EP (East Pacific Ocean), EA (East Atlantic Ocean), IN (Indian Ocean), SWI (Southwest Indian Ocean), NEI (Northeast Indian Ocean), IP (Indo Pacific Ocean).

| **Species** | **GenBank** | **Haplotype** | **Localization** | **Source** |
| --- | --- | --- | --- | --- |
| ***S. attenuata*** | MT906109 | DLOOP1 | SWA(Brazil) | This study |
| *S. attenuata* | MT906110 | DLOOP2 | SWA(Brazil) | This study |
| *S. attenuata* | MT906111 | DLOOP3 | SWA(Brazil) | This study |
| *S. attenuata* | DQ150134 | DLOOP4 | EP | Escorza- Treviño *et al.,*2005 |
| *S. attenuata* | DQ150135 | DLOOP5 | EP | Escorza- Treviño *et al.,*2005 |
| *S. attenuata* | DQ150136 | DLOOP6 | EP | Escorza- Treviño *et al.,*2005 |
| *S. attenuata* | DQ150137 | DLOOP7 | EP | Escorza- Treviño *et al.,*2005 |
| *S. attenuata* | DQ150138 | DLOOP7 | EP | Escorza- Treviño *et al.,*2005 |
| *S. attenuata* | DQ150139 | DLOOP8 | EP | Escorza- Treviño *et al.,*2005 |
| *S. attenuata* | DQ150140 | DLOOP9 | EP | Escorza- Treviño *et al.,*2005 |
| *S. attenuata* | DQ150141 | DLOOP10 | EP | Escorza- Treviño *et al.,*2005 |
| *S. attenuata* | DQ150142 | DLOOP11 | EP | Escorza- Treviño *et al.,*2005 |
| *S. attenuata* | DQ150143 | DLOOP10 | EP | Escorza- Treviño *et al.,*2005 |
| *S. attenuata* | DQ150144 | DLOOP12 | EP | Escorza- Treviño *et al.,*2005 |
| *S. attenuata* | DQ150145 | DLOOP13 | EP | Escorza- Treviño *et al.,*2005 |
| *S. attenuata* | DQ150146 | DLOOP14 | EP | Escorza- Treviño *et al.,*2005 |
| *S. attenuata* | DQ150147 | DLOOP15 | EP | Escorza- Treviño *et al.,*2005 |
| *S. attenuata* | DQ150148 | DLOOP8 | EP | Escorza- Treviño *et al.,*2005 |
| *S. attenuata* | DQ150149 | DLOOP16 | EP | Escorza- Treviño *et al.,*2005 |
| *S. attenuata* | DQ150150 | DLOOP2 | EP | Escorza- Treviño *et al.,*2005 |
| *S. attenuata* | DQ150151 | DLOOP17 | EP | Escorza- Treviño *et al.,*2005 |
| *S. attenuata* | DQ150152 | DLOOP18 | EP | Escorza- Treviño *et al.,*2005 |
| *S. attenuata* | DQ150153 | DLOOP19 | EP | Escorza- Treviño *et al.,*2005 |
| *S. attenuata* | DQ150154 | DLOOP8 | EP | Escorza- Treviño *et al.,*2005 |
| *S. attenuata* | DQ150155 | DLOOP20 | EP | Escorza- Treviño *et al.,*2005 |
| *S. attenuata* | DQ150156 | DLOOP21 | EP | Escorza- Treviño et al.,2005 |
| *S. attenuata* | DQ150157 | DLOOP22 | EP | Escorza- Treviño et al.,2005 |
| *S. attenuata* | DQ150158 | DLOOP23 | EP | Escorza- Treviño et al.,2005 |
| *S. attenuata* | DQ150159 | DLOOP24 | EP | Escorza- Treviño et al.,2005 |
| *S. attenuata* | DQ150160 | DLOOP25 | EP | Escorza- Treviño et al.,2005 |
| *S. attenuata* | DQ150161 | DLOOP26 | EP | Escorza- Treviño et al.,2005 |
| *S. attenuata* | DQ150162 | DLOOP27 | EP | Escorza- Treviño et al.,2005 |
| *S. attenuata* | DQ150163 | DLOOP28 | EP | Escorza- Treviño et al.,2005 |
| *S. attenuata* | DQ150164 | DLOOP29 | EP | Escorza- Treviño et al.,2005 |
| *S. attenuata* | DQ150165 | DLOOP30 | EP | Escorza- Treviño et al.,2005 |
| *S. attenuata* | DQ150166 | DLOOP31 | EP | Escorza- Treviño et al.,2005 |
| *S. attenuata* | DQ150167 | DLOOP5 | EP | Escorza- Treviño et al.,2005 |
| *S. attenuata* | DQ150168 | DLOOP32 | EP | Escorza- Treviño et al.,2005 |
| *S. attenuata* | DQ150169 | DLOOP33 | EP | Escorza- Treviño et al.,2005 |
| *S. attenuata* | DQ150170 | DLOOP34 | EP | Escorza- Treviño et al.,2005 |
| *S. attenuata* | DQ150171 | DLOOP35 | EP | Escorza- Treviño et al.,2005 |
| *S. attenuata* | DQ150172 | DLOOP29 | EP | Escorza- Treviño et al.,2005 |
| *S. attenuata* | DQ150173 | DLOOP36 | EP | Escorza- Treviño et al.,2005 |
| *S. attenuata* | DQ150174 | DLOOP8 | EP | Escorza- Treviño et al.,2005 |
| *S. attenuata* | DQ150175 | DLOOP37 | EP | Escorza- Treviño et al.,2005 |
| *S. attenuata* | DQ150176 | DLOOP38 | EP | Escorza- Treviño et al.,2005 |
| *S. attenuata* | DQ150177 | DLOOP39 | EP | Escorza- Treviño et al.,2005 |
| *S. attenuata* | DQ150178 | DLOOP40 | EP | Escorza- Treviño et al.,2005 |
| *S. attenuata* | DQ150179 | DLOOP41 | EP | Escorza- Treviño et al.,2005 |
| *S. attenuata* | DQ150180 | DLOOP13 | EP | Escorza- Treviño et al.,2005 |
| *S. attenuata* | DQ150181 | DLOOP29 | EP | Escorza- Treviño et al.,2005 |
| *S. attenuata* | DQ150182 | DLOOP42 | EP | Escorza- Treviño et al.,2005 |
| *S. attenuata* | DQ150183 | DLOOP43 | EP | Escorza- Treviño et al.,2005 |
| *S. attenuata* | DQ150184 | DLOOP44 | EP | Escorza- Treviño et al.,2005 |
| *S. attenuata* | DQ150185 | DLOOP45 | EP | Escorza- Treviño et al.,2005 |
| *S. attenuata* | DQ150186 | DLOOP46 | EP | Escorza- Treviño et al.,2005 |
| *S. attenuata* | DQ150187 | DLOOP47 | EP | Escorza- Treviño et al.,2005 |
| *S. attenuata* | DQ150188 | DLOOP48 | EP | Escorza- Treviño et al.,2005 |
| *S. attenuata* | DQ150189 | DLOOP49 | EP | Escorza- Treviño et al.,2005 |
| *S. attenuata* | DQ150190 | DLOOP18 | EP | Escorza- Treviño et al.,2005 |
| *S. attenuata* | DQ150191 | DLOOP8 | EP | Escorza- Treviño et al.,2005 |
| *S. attenuata* | DQ150192 | DLOOP50 | EP | Escorza- Treviño et al.,2005 |
| *S. attenuata* | DQ150193 | DLOOP51 | EP | Escorza- Treviño et al.,2005 |
| *S. attenuata* | DQ150194 | DLOOP52 | EP | Escorza- Treviño et al.,2005 |
| *S. attenuata* | DQ150195 | DLOOP13 | EP | Escorza- Treviño et al.,2005 |
| *S. attenuata* | DQ150196 | DLOOP53 | EP | Escorza- Treviño et al.,2005 |
| *S. attenuata* | DQ150197 | DLOOP23 | EP | Escorza- Treviño et al.,2005 |
| *S. attenuata* | DQ150198 | DLOOP54 | EP | Escorza- Treviño et al.,2005 |
| *S. attenuata* | DQ150199 | DLOOP55 | EP | Escorza- Treviño et al.,2005 |
| *S. attenuata* | DQ150200 | DLOOP56 | EP | Escorza- Treviño et al.,2005 |
| *S. attenuata* | DQ150201 | DLOOP57 | EP | Escorza- Treviño et al.,2005 |
| *S. attenuata* | DQ150202 | DLOOP58 | EP | Escorza- Treviño et al.,2005 |
| *S. attenuata* | DQ150203 | DLOOP59 | EP | Escorza- Treviño et al.,2005 |
| *S. attenuata* | DQ150204 | DLOOP60 | EP | Escorza- Treviño et al.,2005 |
| *S. attenuata* | DQ150205 | DLOOP61 | EP | Escorza- Treviño et al.,2005 |
| *S. attenuata* | DQ150206 | DLOOP62 | EP | Escorza- Treviño et al.,2005 |
| *S. attenuata* | DQ150207 | DLOOP63 | EP | Escorza- Treviño et al.,2005 |
| *S. attenuata* | DQ150208 | DLOOP64 | EP | Escorza- Treviño et al.,2005 |
| *S. attenuata* | DQ150209 | DLOOP65 | EP | Escorza- Treviño et al.,2005 |
| *S. attenuata* | DQ150210 | DLOOP66 | EP | Escorza- Treviño et al.,2005 |
| *S. attenuata* | DQ150211 | DLOOP67 | EP | Escorza- Treviño et al.,2005 |
| *S. attenuata* | DQ150212 | DLOOP68 | EP | Escorza- Treviño et al.,2005 |
| *S. attenuata* | DQ150213 | DLOOP69 | EP | Escorza- Treviño et al.,2005 |
| *S. attenuata* | DQ150214 | DLOOP70 | EP | Escorza- Treviño et al.,2005 |
| *S. attenuata* | DQ150215 | DLOOP7 | EP | Escorza- Treviño et al.,2005 |
| *S. attenuata* | DQ150216 | DLOOP71 | EP | Escorza- Treviño et al.,2005 |
| *S. attenuata* | DQ150217 | DLOOP72 | EP | Escorza- Treviño et al.,2005 |
| *S. attenuata* | DQ150218 | DLOOP73 | EP | Escorza- Treviño et al.,2005 |
| *S. attenuata* | DQ150219 | DLOOP74 | EP | Escorza- Treviño et al.,2005 |
| *S. attenuata* | DQ150220 | DLOOP75 | EP | Escorza- Treviño et al.,2005 |
| *S. attenuata* | DQ150221 | DLOOP18 | EP | Escorza- Treviño et al.,2005 |
| *S. attenuata* | DQ150222 | DLOOP76 | EP | Escorza- Treviño et al.,2005 |
| *S. attenuata* | DQ150223 | DLOOP29 | EP | Escorza- Treviño et al.,2005 |
| *S. attenuata* | DQ150224 | DLOOP8 | EP | Escorza- Treviño et al.,2005 |
| *S. attenuata* | DQ150225 | DLOOP77 | EP | Escorza- Treviño et al.,2005 |
| *S. attenuata* | DQ150226 | DLOOP38 | EP | Escorza- Treviño et al.,2005 |
| *S. attenuata* | DQ150227 | DLOOP8 | EP | Escorza- Treviño et al.,2005 |
| *S. attenuata* | DQ150228 | DLOOP5 | EP | Escorza- Treviño et al.,2005 |
| *S. attenuata* | DQ150229 | DLOOP78 | EP | Escorza- Treviño et al.,2005 |
| *S. attenuata* | DQ150230 | DLOOP79 | EP | Escorza- Treviño et al.,2005 |
| *S. attenuata* | DQ150231 | DLOOP80 | EP | Escorza- Treviño et al.,2005 |
| *S. attenuata* | DQ150232 | DLOOP57 | EP | Escorza- Treviño et al.,2005 |
| *S. attenuata* | DQ150233 | DLOOP81 | EP | Escorza- Treviño et al.,2005 |
| *S. attenuata* | DQ150234 | DLOOP82 | EP | Escorza- Treviño et al.,2005 |
| *S. attenuata* | DQ150235 | DLOOP83 | EP | Escorza- Treviño et al.,2005 |
| *S. attenuata* | DQ150236 | DLOOP84 | EP | Escorza- Treviño et al.,2005 |
| *S. attenuata* | DQ150237 | DLOOP8 | EP | Escorza- Treviño et al.,2005 |
| *S. attenuata* | DQ150238 | DLOOP85 | EP | Escorza- Treviño et al.,2005 |
| *S. attenuata* | DQ150239 | DLOOP8 | EP | Escorza- Treviño et al.,2005 |
| *S. attenuata* | DQ150240 | DLOOP66 | EP | Escorza- Treviño et al.,2005 |
| *S. attenuata* | DQ150241 | DLOOP86 | EP | Escorza- Treviño et al.,2005 |
| *S. attenuata* | DQ150242 | DLOOP87 | EP | Escorza- Treviño et al.,2005 |
| *S. attenuata* | DQ150243 | DLOOP88 | EP | Escorza- Treviño et al.,2005 |
| *S. attenuata* | DQ150244 | DLOOP89 | EP | Escorza- Treviño et al.,2005 |
| *S. attenuata* | DQ150245 | DLOOP90 | EP | Escorza- Treviño et al.,2005 |
| *S. attenuata* | DQ845442 | DLOOP91 | NWA | Kingston et al.,2009 |
| *S. attenuata* | DQ845443 | DLOOP92 | NWA | Kingston et al.,2009 |
| *S. attenuata* | GQ504120 | DLOOP1 | NWA | Kingston et al.,2009 |
| *S. attenuata* | GQ504121 | DLOOP93 | NWA | Kingston et al.,2009 |
| *S. attenuata* | GQ504122 | DLOOP3 | NWA | Kingston et al.,2009 |
| *S. attenuata* | GQ504123 | DLOOP94 | NWA | Kingston et al.,2009 |
| *S. attenuata* | GQ504124 | DLOOP95 | NWA | Kingston et al.,2009 |
| *S. attenuata* | GQ504126 | DLOOP53 | NEA | Kingston et al.,2009 |
| *S. attenuata* | GQ504127 | DLOOP6 | NEA | Kingston et al.,2009 |
| *S. attenuata* | GQ504128 | DLOOP96 | NEA | Kingston et al.,2009 |
| *S. attenuata* | GQ504129 | DLOOP8 | NEA | Kingston et al.,2009 |
| *S. attenuata* | GQ504130 | DLOOP70 | NEA | Kingston et al.,2009 |
| *S. attenuata* | GQ504131 | DLOOP8 | NEA | Kingston et al.,2009 |
| *S. attenuata* | GQ852567 | DLOOP97 | NEA | Courbis et al.,2014 |
| *S. attenuata* | GQ852568 | DLOOP98 | NEA | Courbis et al.,2014 |
| *S. attenuata* | GQ852569 | DLOOP8 | NEA | Courbis et al.,2014 |
| *S. attenuata* | GQ852570 | DLOOP99 | NEA | Courbis et al.,2014 |
| *S. attenuata* | GQ852571 | DLOOP17 | NEA | Courbis et al.,2014 |
| *S. attenuata* | GQ852572 | DLOOP70 | NEA | Courbis et al.,2014 |
| *S. attenuata* | GQ852573 | DLOOP100 | NEA | Courbis et al.,2014 |
| *S. attenuata* | GQ852574 | DLOOP101 | NEA | Courbis et al.,2014 |
| *S. attenuata* | GQ852575 | DLOOP65 | NEA | Courbis et al.,2014 |
| *S. attenuata* | GQ852576 | DLOOP102 | NEA | Courbis et al.,2014 |
| *S. attenuata* | GQ852577 | DLOOP31 | NEA | Courbis et al.,2014 |
| *S. attenuata* | GQ852578 | DLOOP103 | NEA | Courbis et al.,2014 |
| *S. attenuata* | GQ852579 | DLOOP8 | NEA | Courbis et al.,2014 |
| *S. attenuata* | GU136595 | DLOOP104 | NEA | Courbis et al.,2014 |
| *S. attenuata* | GU256406 | DLOOP95 | NEA | Courbis et al.,2014 |
| *S. attenuata* | KP756626 | DLOOP105 | SWP | Oremus et al.,2015 |
| *S. attenuata* | KP756627 | DLOOP65 | SWP | Oremus et al.,2015 |
| *S. attenuata* | KP756628 | DLOOP106 | SWP | Oremus et al.,2015 |
| *S. attenuata* | KP756629 | DLOOP8 | SWP | Oremus et al.,2015 |
| *S. attenuata* | KP756630 | DLOOP107 | SWP | Oremus et al.,2015 |
| *S. attenuata* | KP756631 | DLOOP8 | SWP | Oremus et al.,2015 |
| *S. attenuata* | KP756632 | DLOOP8 | SWP | Oremus et al.,2015 |
| *S. attenuata* | KP756633 | DLOOP8 | SWP | Oremus et al.,2015 |
| *S. attenuata* | KP756634 | DLOOP47 | SWP | Oremus et al.,2015 |
| *S. attenuata* | KP756635 | DLOOP25 | SWP | Oremus et al.,2015 |
| *S. attenuata* | KP756636 | DLOOP108 | SWP | Oremus et al.,2015 |
| *S. attenuata* | KP756637 | DLOOP23 | SWP | Oremus et al.,2015 |
| *S. attenuata* | KP756638 | DLOOP23 | SWP | Oremus et al.,2015 |
| *S. attenuata* | KP756639 | DLOOP109 | SWP | Oremus et al.,2015 |
| *S. attenuata* | KP756640 | DLOOP109 | SWP | Oremus et al.,2015 |
| *S. attenuata* | KP756646 | DLOOP110 | SWP | Oremus et al.,2015 |
| ***S. clymene*** | MT906112 | DLOOP111 | SWA(Brazil) | This study |
| *S. clymene* | MT906113 | DLOOP112 | SWA(Brazil) | This study |
| *S. clymene* | MT906114 | DLOOP113 | SWA(Brazil) | This study |
| *S. clymene* | MT906115 | DLOOP114 | SWA(Brazil) | This study |
| *S. clymene* | MT906116 | DLOOP115 | SWA(Brazil) | This study |
| *S. clymene* | MT906117 | DLOOP116 | SWA(Brazil) | This study |
| *S. clymene* | MT906118 | DLOOP117 | SWA(Brazil) | This study |
| *S. clymene* | MT906119 | DLOOP118 | SWA(Brazil) | This study |
| *S. clymene* | MT906120 | DLOOP119 | SWA(Brazil) | This study |
| *S. clymene* | MT906121 | DLOOP120 | SWA(Brazil) | This study |
| *S. clymene* | MT906122 | DLOOP121 | SWA(Brazil) | This study |
| *S. clymene* | MT906123 | DLOOP122 | SWA(Brazil) | This study |
| *S. clymene* | GQ504137 | DLOOP123 | NWA | Kingston et al.,2009 |
| *S. clymene* | GQ504138 | DLOOP124 | NWA | Kingston et al.,2009 |
| *S. clymene* | GQ504139 | DLOOP125 | NWA | Kingston et al.,2009 |
| *S. clymene* | GQ504140 | DLOOP126 | NWA | Kingston et al.,2009 |
| *S. clymene* | GQ504141 | DLOOP127 | NWA | Kingston et al.,2009 |
| *S. clymene* | GQ504142 | DLOOP128 | NWA | Kingston et al.,2009 |
| *S. clymene* | GQ504143 | DLOOP129 | NWA | Kingston et al.,2009 |
| *S. clymene* | GQ504144 | DLOOP130 | NWA | Kingston et al.,2009 |
| *S. clymene* | GQ504145 | DLOOP131 | NWA | Kingston et al.,2009 |
| *S. clymene* | GQ504146 | DLOOP132 | NWA | Kingston et al.,2009 |
| *S. clymene* | GQ504147 | DLOOP120 | NWA | Kingston et al.,2009 |
| *S. clymene* | GQ504148 | DLOOP133 | NWA | Kingston et al.,2009 |
| *S. clymene* | DQ845446 | DLOOP134 | NWA | Kingston et al.,2009 |
| *S. clymene* | DQ845447 | DLOOP135 | NWA | Kingston et al.,2009 |
| ***S. coeruleoalba*** | MT906124 | DLOOP136 | SWA (Brazil) | This study |
| *S. coeruleoalba* | MT906125 | DLOOP137 | SWA (Brazil) | This study |
| *S. coeruleoalba* | MT906126 | DLOOP138 | SWA (Brazil) | This study |
| *S. coeruleoalba* | MT906127 | DLOOP139 | SWA (Brazil) | This study |
| *S. coeruleoalba* | MT906128 | DLOOP140 | SWA (Brazil) | This study |
| *S. coeruleoalba* | MT906129 | DLOOP141 | SWA (Brazil) | This study |
| *S. coeruleoalba* | MT906130 | DLOOP142 | SWA (Brazil) | This study |
| *S. coeruleoalba* | MT906124 | DLOOP143 | SWA (Brazil) | This study |
| *S. coeruleoalba* | GQ504149 | DLOOP144 | NWA | Kingston et al.,2009 |
| *S. coeruleoalba* | GQ504150 | DLOOP145 | NWA | Kingston et al.,2009 |
| *S. coeruleoalba* | GQ504152 | DLOOP146 | NWA | Kingston et al.,2009 |
| *S. coeruleoalba* | GQ504153 | DLOOP147 | NWA | Kingston et al.,2009 |
| *S. coeruleoalba* | GQ504154 | DLOOP148 | NWA | Kingston et al.,2009 |
| *S. coeruleoalba* | GQ504155 | DLOOP149 | NWA | Kingston et al.,2009 |
| *S. coeruleoalba* | GQ504156 | DLOOP150 | NWA | Kingston et al.,2009 |
| *S. coeruleoalba* | GQ504157 | DLOOP151 | NWA | Kingston et al.,2009 |
| *S. coeruleoalba* | GQ504158 | DLOOP152 | NWA | Kingston et al.,2009 |
| *S. coeruleoalba* | GQ504159 | DLOOP153 | NWA | Kingston et al.,2009 |
| *S. coeruleoalba* | GQ504160 | DLOOP154 | NWA | Kingston et al.,2009 |
| *S. coeruleoalba* | GQ504161 | DLOOP155 | NWA | Kingston et al.,2009 |
| *S. coeruleoalba* | GQ504162 | DLOOP156 | NWA | Kingston et al.,2009 |
| *S. coeruleoalba* | GQ504163 | DLOOP157 | NWA | Kingston et al.,2009 |
| *S. coeruleoalba* | GQ504164 | DLOOP158 | NWA | Kingston et al.,2009 |
| *S. coeruleoalba* | DQ845440 | DLOOP159 | NWA | Kingston et al.,2009 |
| *S. coeruleoalba* | DQ845441 | DLOOP160 | NWA | Kingston et al.,2009 |
| ***S. frontalis*** | MT906132 | DLOOP161 | SWA (Brazil) | This study |
| *S. frontalis* | MT906133 | DLOOP162 | SWA (Brazil) | This study |
| *S. frontalis* | MT906134 | DLOOP163 | SWA (Brazil) | This study |
| *S. frontalis* | MT906135 | DLOOP164 | SWA (Brazil) | This study |
| *S. frontalis* | DQ060054 | DLOOP165 | NWA | Adams et al.,2006 |
| *S. frontalis* | DQ060055 | DLOOP166 | NWA | Adams et al.,2006 |
| *S. frontalis* | DQ060056 | DLOOP161 | NWA | Adams et al.,2006 |
| *S. frontalis* | DQ060057 | DLOOP167 | NWA | Adams et al.,2006 |
| *S. frontalis* | DQ060058 | DLOOP168 | NWA | Adams et al.,2006 |
| *S. frontalis* | DQ060059 | DLOOP169 | NWA | Adams et al.,2006 |
| *S. frontalis* | DQ060060 | DLOOP170 | NWA | Adams et al.,2006 |
| *S. frontalis* | DQ060061 | DLOOP171 | NWA | Adams et al.,2006 |
| *S. frontalis* | DQ060062 | DLOOP172 | NWA | Adams et al.,2006 |
| *S. frontalis* | DQ060063 | DLOOP173 | NWA | Adams et al.,2006 |
| *S. frontalis* | DQ060064 | DLOOP174 | NWA | Adams et al.,2006 |
| *S. frontalis* | EF546440 | DLOOP167 | NWA | Green et al.,2007 |
| *S. frontalis* | EF682650 | DLOOP165 | NEA | Querouil et al.,2010 |
| *S. frontalis* | EF682651 | DLOOP175 | NEA | Querouil et al.,2010 |
| *S. frontalis* | EF682652 | DLOOP176 | NEA | Querouil et al.,2010 |
| *S. frontalis* | EF682653 | DLOOP175 | NEA | Querouil et al.,2010 |
| *S. frontalis* | EF682654 | DLOOP175 | NEA | Querouil et al.,2010 |
| *S. frontalis* | EF682655 | DLOOP174 | NEA | Querouil et al.,2010 |
| *S. frontalis* | EF682656 | DLOOP177 | NEA | Querouil et al.,2010 |
| *S. frontalis* | EF682657 | DLOOP178 | NEA | Querouil et al.,2010 |
| *S. frontalis* | EF682658 | DLOOP1 | NEA | Querouil et al.,2010 |
| *S. frontalis* | EF682659 | DLOOP3 | NEA | Querouil et al.,2010 |
| *S. frontalis* | EF682660 | DLOOP179 | NEA | Querouil et al.,2010 |
| *S. frontalis* | EF682661 | DLOOP161 | NEA | Querouil et al.,2010 |
| *S. frontalis* | EF682662 | DLOOP180 | NEA | Querouil et al.,2010 |
| *S. frontalis* | EF682663 | DLOOP181 | NEA | Querouil et al.,2010 |
| *S. frontalis* | EF682664 | DLOOP182 | NEA | Querouil et al.,2010 |
| *S. frontalis* | EF682665 | DLOOP175 | NEA | Querouil et al.,2010 |
| *S. frontalis* | EF682666 | DLOOP183 | NEA | Querouil et al.,2010 |
| *S. frontalis* | EF682667 | DLOOP93 | NEA | Querouil et al.,2010 |
| *S. frontalis* | EF682668 | DLOOP161 | NEA | Querouil et al.,2010 |
| *S. frontalis* | EF682669 | DLOOP165 | NEA | Querouil et al.,2010 |
| *S. frontalis* | EF682670 | DLOOP184 | NEA | Querouil et al.,2010 |
| *S. frontalis* | EF682671 | DLOOP163 | NEA | Querouil et al.,2010 |
| *S. frontalis* | EF682672 | DLOOP165 | NEA | Querouil et al.,2010 |
| *S. frontalis* | EF682673 | DLOOP185 | NEA | Querouil et al.,2010 |
| *S. frontalis* | EF682674 | DLOOP175 | NEA | Querouil et al.,2010 |
| *S. frontalis* | EF682675 | DLOOP186 | NEA | Querouil et al.,2010 |
| *S. frontalis* | EF682676 | DLOOP187 | NEA | Querouil et al.,2010 |
| *S. frontalis* | EF682677 | DLOOP165 | NEA | Querouil et al.,2010 |
| *S. frontalis* | EF682678 | DLOOP165 | NEA | Querouil et al.,2010 |
| *S. frontalis* | EF682679 | DLOOP174 | NEA | Querouil et al.,2010 |
| *S. frontalis* | EF682680 | DLOOP180 | NEA | Querouil et al.,2010 |
| *S. frontalis* | EF682681 | DLOOP188 | NEA | Querouil et al.,2010 |
| *S. frontalis* | EF682682 | DLOOP189 | NEA | Querouil et al.,2010 |
| *S. frontalis* | EF682683 | DLOOP161 | NEA | Querouil et al.,2010 |
| *S. frontalis* | EF682684 | DLOOP165 | NEA | Querouil et al.,2010 |
| *S. frontalis* | EF682685 | DLOOP167 | NEA | Querouil et al.,2010 |
| *S. frontalis* | EF682686 | DLOOP190 | NEA | Querouil et al.,2010 |
| *S. frontalis* | EF682687 | DLOOP191 | NEA | Querouil et al.,2010 |
| *S. frontalis* | EF682688 | DLOOP192 | NEA | Querouil et al.,2010 |
| *S. frontalis* | EF682689 | DLOOP175 | NEA | Querouil et al.,2010 |
| *S. frontalis* | EF682690 | DLOOP181 | NEA | Querouil et al.,2010 |
| *S. frontalis* | EF682691 | DLOOP161 | NEA | Querouil et al.,2010 |
| *S. frontalis* | EF682692 | DLOOP181 | NEA | Querouil et al.,2010 |
| *S. frontalis* | EF682693 | DLOOP193 | NEA | Querouil et al.,2010 |
| *S. frontalis* | EF682694 | DLOOP194 | NEA | Querouil et al.,2010 |
| *S. frontalis* | EF682695 | DLOOP175 | NEA | Querouil et al.,2010 |
| *S. frontalis* | EF682696 | DLOOP167 | NEA | Querouil et al.,2010 |
| *S. frontalis* | EF682697 | DLOOP195 | NEA | Querouil et al.,2010 |
| *S. frontalis* | EF682698 | DLOOP3 | NEA | Querouil et al.,2010 |
| *S. frontalis* | EF682699 | DLOOP161 | NEA | Querouil et al.,2010 |
| *S. frontalis* | EF682700 | DLOOP161 | NEA | Querouil et al.,2010 |
| *S. frontalis* | EF682701 | DLOOP196 | NEA | Querouil et al.,2010 |
| *S. frontalis* | EF682702 | DLOOP197 | NEA | Querouil et al.,2010 |
| *S. frontalis* | EF682703 | DLOOP1 | NEA | Querouil et al.,2010 |
| *S. frontalis* | EF682704 | DLOOP161 | NEA | Querouil et al.,2010 |
| *S. frontalis* | EF682705 | DLOOP3 | NEA | Querouil et al.,2010 |
| *S. frontalis* | EF682706 | DLOOP175 | NEA | Querouil et al.,2010 |
| *S. frontalis* | EF682707 | DLOOP175 | NEA | Querouil et al.,2010 |
| *S. frontalis* | EF682708 | DLOOP193 | NEA | Querouil et al.,2010 |
| *S. frontalis* | EF682709 | DLOOP181 | NEA | Querouil et al.,2010 |
| *S. frontalis* | EF682710 | DLOOP181 | NEA | Querouil et al.,2010 |
| *S. frontalis* | EF682711 | DLOOP175 | NEA | Querouil et al.,2010 |
| *S. frontalis* | EF682712 | DLOOP189 | NEA | Querouil et al.,2010 |
| *S. frontalis* | EF682713 | DLOOP198 | NEA | Querouil et al.,2010 |
| *S. frontalis* | EF682714 | DLOOP189 | NEA | Querouil et al.,2010 |
| *S. frontalis* | EF682715 | DLOOP199 | NEA | Querouil et al.,2010 |
| *S. frontalis* | EF682716 | DLOOP200 | NEA | Querouil et al.,2010 |
| *S. frontalis* | EF682717 | DLOOP195 | NEA | Querouil et al.,2010 |
| *S. frontalis* | EF682718 | DLOOP189 | NEA | Querouil et al.,2010 |
| *S. frontalis* | EF682719 | DLOOP175 | NEA | Querouil et al.,2010 |
| *S. frontalis* | EF682720 | DLOOP171 | NEA | Querouil et al.,2010 |
| *S. frontalis* | EF682721 | DLOOP3 | NEA | Querouil et al.,2010 |
| *S. frontalis* | EF682722 | DLOOP165 | NEA | Querouil et al.,2010 |
| *S. frontalis* | EF682723 | DLOOP171 | NEA | Querouil et al.,2010 |
| *S. frontalis* | EF682724 | DLOOP161 | NEA | Querouil et al.,2010 |
| *S. frontalis* | EF682725 | DLOOP201 | NEA | Querouil et al.,2010 |
| *S. frontalis* | EF682726 | DLOOP166 | NEA | Querouil et al.,2010 |
| *S. frontalis* | EF682727 | DLOOP161 | NEA | Querouil et al.,2010 |
| *S. frontalis* | EF682728 | DLOOP184 | NEA | Querouil et al.,2010 |
| *S. frontalis* | EF682729 | DLOOP202 | NEA | Querouil et al.,2010 |
| *S. frontalis* | EF682730 | DLOOP203 | NEA | Querouil et al.,2010 |
| *S. frontalis* | EF682731 | DLOOP161 | NEA | Querouil et al.,2010 |
| *S. frontalis* | EF682732 | DLOOP165 | NEA | Querouil et al.,2010 |
| *S. frontalis* | EF682733 | DLOOP166 | NEA | Querouil et al.,2010 |
| *S. frontalis* | EF682734 | DLOOP177 | NEA | Querouil et al.,2010 |
| *S. frontalis* | EF682735 | DLOOP161 | NEA | Querouil et al.,2010 |
| *S. frontalis* | EF682736 | DLOOP204 | NEA | Querouil et al.,2010 |
| *S. frontalis* | EF682737 | DLOOP161 | NEA | Querouil et al.,2010 |
| *S. frontalis* | EF682738 | DLOOP205 | NEA | Querouil et al.,2010 |
| *S. frontalis* | EF682739 | DLOOP175 | NEA | Querouil et al.,2010 |
| *S. frontalis* | EF682740 | DLOOP175 | NEA | Querouil et al.,2010 |
| *S. frontalis* | EF682741 | DLOOP161 | NEA | Querouil et al.,2010 |
| *S. frontalis* | EF682742 | DLOOP161 | NEA | Querouil et al.,2010 |
| *S. frontalis* | EF682743 | DLOOP206 | NEA | Querouil et al.,2010 |
| *S. frontalis* | EF682744 | DLOOP165 | NEA | Querouil et al.,2010 |
| *S. frontalis* | EF682745 | DLOOP207 | NEA | Querouil et al.,2010 |
| *S. frontalis* | EF682746 | DLOOP167 | NEA | Querouil et al.,2010 |
| *S. frontalis* | EF682747 | DLOOP208 | NEA | Querouil et al.,2010 |
| *S. frontalis* | EF682748 | DLOOP1 | NEA | Querouil et al.,2010 |
| *S. frontalis* | EF682749 | DLOOP161 | NEA | Querouil et al.,2010 |
| *S. frontalis* | EF682750 | DLOOP165 | NEA | Querouil et al.,2010 |
| *S. frontalis* | EF682751 | DLOOP161 | NEA | Querouil et al.,2010 |
| *S. frontalis* | EF682752 | DLOOP165 | NEA | Querouil et al.,2010 |
| *S. frontalis* | EF682753 | DLOOP209 | NEA | Querouil et al.,2010 |
| *S. frontalis* | EF682754 | DLOOP210 | NEA | Querouil et al.,2010 |
| *S. frontalis* | EF682755 | DLOOP165 | NEA | Querouil et al.,2010 |
| *S. frontalis* | EF682756 | DLOOP193 | NEA | Querouil et al.,2010 |
| *S. frontalis* | EF682757 | DLOOP179 | NEA | Querouil et al.,2010 |
| *S. frontalis* | EF682758 | DLOOP211 | NEA | Querouil et al.,2010 |
| *S. frontalis* | EF682759 | DLOOP212 | NEA | Querouil et al.,2010 |
| *S. frontalis* | EF682760 | DLOOP181 | NEA | Querouil et al.,2010 |
| *S. frontalis* | EF682761 | DLOOP189 | NEA | Querouil et al.,2010 |
| *S. frontalis* | EF682762 | DLOOP213 | NEA | Querouil et al.,2010 |
| *S. frontalis* | EF682763 | DLOOP181 | NEA | Querouil et al.,2010 |
| *S. frontalis* | EF682764 | DLOOP214 | NEA | Querouil et al.,2010 |
| *S. frontalis* | EF682765 | DLOOP202 | NEA | Querouil et al.,2010 |
| *S. frontalis* | EF682766 | DLOOP180 | NEA | Querouil et al.,2010 |
| *S. frontalis* | EF682767 | DLOOP215 | NEA | Querouil et al.,2010 |
| *S. frontalis* | EF682768 | DLOOP207 | NEA | Querouil et al.,2010 |
| *S. frontalis* | EF682769 | DLOOP165 | NEA | Querouil et al.,2010 |
| *S. frontalis* | EF682770 | DLOOP165 | NEA | Querouil et al.,2010 |
| *S. frontalis* | EF682771 | DLOOP180 | NEA | Querouil et al.,2010 |
| *S. frontalis* | EF682772 | DLOOP216 | NEA | Querouil et al.,2010 |
| *S. frontalis* | EF682773 | DLOOP210 | NEA | Querouil et al.,2010 |
| *S. frontalis* | EF682774 | DLOOP190 | NEA | Querouil et al.,2010 |
| *S. frontalis* | EF682775 | DLOOP217 | NEA | Querouil et al.,2010 |
| *S. frontalis* | EF682776 | DLOOP181 | NEA | Querouil et al.,2010 |
| *S. frontalis* | EF682777 | DLOOP1 | NEA | Querouil et al.,2010 |
| *S. frontalis* | EF682778 | DLOOP167 | NEA | Querouil et al.,2010 |
| *S. frontalis* | EF682779 | DLOOP165 | NEA | Querouil et al.,2010 |
| *S. frontalis* | EF682780 | DLOOP219 | NEA | Querouil et al.,2010 |
| *S. frontalis* | EF682781 | DLOOP174 | NEA | Querouil et al.,2010 |
| *S. frontalis* | EF682782 | DLOOP165 | NEA | Querouil et al.,2010 |
| *S. frontalis* | EF682783 | DLOOP179 | NEA | Querouil et al.,2010 |
| *S. frontalis* | EF682784 | DLOOP175 | NEA | Querouil et al.,2010 |
| *S. frontalis* | EF682785 | DLOOP219 | NEA | Querouil et al.,2010 |
| *S. frontalis* | EF682786 | DLOOP165 | NEA | Querouil et al.,2010 |
| *S. frontalis* | EF682787 | DLOOP161 | NEA | Querouil et al.,2010 |
| *S. frontalis* | EF682788 | DLOOP189 | NEA | Querouil et al.,2010 |
| *S. frontalis* | EF682789 | DLOOP3 | NEA | Querouil et al.,2010 |
| *S. frontalis* | EF682790 | DLOOP189 | NEA | Querouil et al.,2010 |
| *S. frontalis* | EF682791 | DLOOP165 | NEA | Querouil et al.,2010 |
| *S. frontalis* | EF682792 | DLOOP161 | NEA | Querouil et al.,2010 |
| *S. frontalis* | EF682793 | DLOOP161 | NEA | Querouil et al.,2010 |
| *S. frontalis* | EF682794 | DLOOP161 | NEA | Querouil et al.,2010 |
| *S. frontalis* | EF682795 | DLOOP180 | NEA | Querouil et al.,2010 |
| *S. frontalis* | EF682796 | DLOOP187 | NEA | Querouil et al.,2010 |
| *S. frontalis* | EF682797 | DLOOP220 | NEA | Querouil et al.,2010 |
| *S. frontalis* | EF682798 | DLOOP163 | NEA | Querouil et al.,2010 |
| *S. frontalis* | EF682799 | DLOOP221 | NEA | Querouil et al.,2010 |
| *S. frontalis* | EF682800 | DLOOP189 | NEA | Querouil et al.,2010 |
| *S. frontalis* | EF682801 | DLOOP174 | NEA | Querouil et al.,2010 |
| *S. frontalis* | EF682802 | DLOOP222 | NEA | Querouil et al.,2010 |
| *S. frontalis* | EF682803 | DLOOP161 | NEA | Querouil et al.,2010 |
| *S. frontalis* | EF682804 | DLOOP223 | NEA | Querouil et al.,2010 |
| *S. frontalis* | EF682805 | DLOOP165 | NEA | Querouil et al.,2010 |
| *S. frontalis* | EF682806 | DLOOP161 | NEA | Querouil et al.,2010 |
| *S. frontalis* | EF682807 | DLOOP224 | NEA | Querouil et al.,2010 |
| *S. frontalis* | EF682808 | DLOOP225 | NEA | Querouil et al.,2010 |
| *S. frontalis* | EF682809 | DLOOP202 | NEA | Querouil et al.,2010 |
| *S. frontalis* | EF682810 | DLOOP226 | NEA | Querouil et al.,2010 |
| *S. frontalis* | EF682811 | DLOOP226 | NEA | Querouil et al.,2010 |
| *S. frontalis* | EF682812 | DLOOP3 | NEA | Querouil et al.,2010 |
| *S. frontalis* | EF682813 | DLOOP161 | NEA | Querouil et al.,2010 |
| *S. frontalis* | EF682814 | DLOOP184 | NEA | Querouil et al.,2010 |
| *S. frontalis* | EF682815 | DLOOP165 | NEA | Querouil et al.,2010 |
| *S. frontalis* | EF682816 | DLOOP190 | NEA | Querouil et al.,2010 |
| *S. frontalis* | EF682817 | DLOOP181 | NEA | Querouil et al.,2010 |
| *S. frontalis* | EF682818 | DLOOP211 | NEA | Querouil et al.,2010 |
| *S. frontalis* | EF682819 | DLOOP161 | NEA | Querouil et al.,2010 |
| *S. frontalis* | EF682820 | DLOOP227 | NEA | Querouil et al.,2010 |
| *S. frontalis* | EF682821 | DLOOP175 | NEA | Querouil et al.,2010 |
| *S. frontalis* | EF682822 | DLOOP180 | NEA | Querouil et al.,2010 |
| *S. frontalis* | EF682823 | DLOOP175 | NEA | Querouil et al.,2010 |
| *S. frontalis* | EF682824 | DLOOP181 | NEA | Querouil et al.,2010 |
| *S. frontalis* | EF682825 | DLOOP3 | NEA | Querouil et al.,2010 |
| *S. frontalis* | EF682826 | DLOOP202 | NEA | Querouil et al.,2010 |
| *S. frontalis* | EF682827 | DLOOP228 | NEA | Querouil et al.,2010 |
| *S. frontalis* | EF682828 | DLOOP167 | NEA | Querouil et al.,2010 |
| *S. frontalis* | EF682829 | DLOOP177 | NEA | Querouil et al.,2010 |
| *S. frontalis* | EF682830 | DLOOP229 | NEA | Querouil et al.,2010 |
| *S. frontalis* | EF682831 | DLOOP230 | NEA | Querouil et al.,2010 |
| *S. frontalis* | EF682832 | DLOOP231 | NEA | Querouil et al.,2010 |
| *S. frontalis* | EF682833 | DLOOP232 | NEA | Querouil et al.,2010 |
| *S. frontalis* | EF682834 | DLOOP229 | NEA | Querouil et al.,2010 |
| *S. frontalis* | EF682835 | DLOOP233 | NEA | Querouil et al.,2010 |
| *S. frontalis* | EF682836 | DLOOP195 | NEA | Querouil et al.,2010 |
| *S. frontalis* | EF682837 | DLOOP234 | NEA | Querouil et al.,2010 |
| *S. frontalis* | EF682838 | DLOOP221 | NEA | Querouil et al.,2010 |
| *S. frontalis* | EF682839 | DLOOP175 | NEA | Querouil et al.,2010 |
| *S. frontalis* | EF682840 | DLOOP191 | NEA | Querouil et al.,2010 |
| *S. frontalis* | GQ504170 | DLOOP235 | NWA | Kingston et al.,2009 |
| *S. frontalis* | GQ504171 | DLOOP236 | NWA | Kingston et al.,2009 |
| *S. frontalis* | GQ504172 | DLOOP237 | NWA | Kingston et al.,2009 |
| *S. frontalis* | GQ504173 | DLOOP166 | NWA | Kingston et al.,2009 |
| *S. frontalis* | GQ504174 | DLOOP191 | NWA | Kingston et al.,2009 |
| *S. frontalis* | GQ504175 | DLOOP238 | NWA | Kingston et al.,2009 |
| *S. frontalis* | GQ504176 | DLOOP239 | NWA | Kingston et al.,2009 |
| *S. frontalis* | GQ504177 | DLOOP184 | NWA | Kingston et al.,2009 |
| *S. frontalis* | GQ504178 | DLOOP189 | NWA | Kingston et al.,2009 |
| *S. frontalis* | GQ504179 | DLOOP175 | NWA | Kingston et al.,2009 |
| *S. frontalis* | GQ504180 | DLOOP180 | NWA | Kingston et al.,2009 |
| *S. frontalis* | GQ504181 | DLOOP240 | NWA | Kingston et al.,2009 |
| *S. frontalis* | GQ504182 | DLOOP241 | NWA | Kingston et al.,2009 |
| *S. frontalis* | GQ504183 | DLOOP221 | NWA | Kingston et al.,2009 |
| *S. frontalis* | GQ504184 | DLOOP242 | NWA | Kingston et al.,2009 |
| *S. frontalis* | GQ504185 | DLOOP243 | NWA | Kingston et al.,2009 |
| *S. frontalis* | GQ504186 | DLOOP244 | NWA | Kingston et al.,2009 |
| *S. frontalis* | GQ504187 | DLOOP245 | NWA | Kingston et al.,2009 |
| *S. frontalis* | GQ504188 | DLOOP246 | NWA | Kingston et al.,2009 |
| *S. frontalis* | GQ504189 | DLOOP247 | NWA | Kingston et al.,2009 |
| *S. frontalis* | GQ504190 | DLOOP248 | NWA | Kingston et al.,2009 |
| *S. frontalis* | GQ504191 | DLOOP249 | NWA | Kingston et al.,2009 |
| *S. frontalis* | GQ504192 | DLOOP250 | NWA | Kingston et al.,2009 |
| *S. frontalis* | GQ504193 | DLOOP181 | NWA | Kingston et al.,2009 |
| *S. frontalis* | GQ504194 | DLOOP251 | NWA | Kingston et al.,2009 |
| *S. frontalis* | GQ504195 | DLOOP252 | NWA | Kingston et al.,2009 |
| *S. frontalis* | KC204733 | DLOOP163 | SWA | Caballero et al.,2013 |
| *S. frontalis* | KC204734 | DLOOP166 | SWA | Caballero et al.,2013 |
| *S. frontalis* | KC204735 | DLOOP162 | SWA | Caballero et al.,2013 |
| *S. frontalis* | KC204736 | DLOOP253 | NWA | Caballero et al.,2013 |
| *S. frontalis* | KC204737 | DLOOP248 | NWA | Caballero et al.,2013 |
| *S. frontalis* | KC204738 | DLOOP254 | NWA | Caballero et al.,2013 |
| *S. frontalis* | KC204739 | DLOOP1 | NWA | Caballero et al.,2013 |
| *S. frontalis* | KC204740 | DLOOP255 | NWA | Caballero et al.,2013 |
| *S. frontalis* | EU121116 | DLOOP254 | NWA | Caballero et al.,2008 |
| *S. frontalis* | JX414567 | DLOOP256 | NWA | Viricel et al.,2014 |
| *S. frontalis* | JX414568 | DLOOP190 | NWA | Viricel et al.,2014 |
| *S. frontalis* | JX414569 | DLOOP257 | NWA | Viricel et al.,2014 |
| *S. frontalis* | JX414570 | DLOOP258 | NWA | Viricel et al.,2014 |
| *S. frontalis* | JX414571 | DLOOP161 | NWA | Viricel et al.,2014 |
| *S. frontalis* | JX414572 | DLOOP259 | NWA | Viricel et al.,2014 |
| *S. frontalis* | JX414573 | DLOOP260 | NWA | Viricel et al.,2014 |
| *S. frontalis* | JX414574 | DLOOP210 | NWA | Viricel et al.,2014 |
| *S. frontalis* | JX414575 | DLOOP261 | NWA | Viricel et al.,2014 |
| *S. frontalis* | JX414576 | DLOOP262 | NWA | Viricel et al.,2014 |
| *S. frontalis* | JX414577 | DLOOP179 | NWA | Viricel et al.,2014 |
| *S. frontalis* | JX414578 | DLOOP195 | NWA | Viricel et al.,2014 |
| *S. frontalis* | JX414579 | DLOOP165 | NWA | Viricel et al.,2014 |
| *S. frontalis* | JX414580 | DLOOP178 | NWA | Viricel et al.,2014 |
| *S. frontalis* | JX414581 | DLOOP263 | NWA | Viricel et al.,2014 |
| *S. frontalis* | JX414582 | DLOOP264 | NWA | Viricel et al.,2014 |
| *S. frontalis* | JX414583 | DLOOP265 | NWA | Viricel et al.,2014 |
| *S. frontalis* | JX414584 | DLOOP266 | NWA | Viricel et al.,2014 |
| *S. frontalis* | JX414585 | DLOOP194 | NWA | Viricel et al.,2014 |
| *S. frontalis* | JX414586 | DLOOP267 | NWA | Viricel et al.,2014 |
| *S. frontalis* | JX414587 | DLOOP268 | NWA | Viricel et al.,2014 |
| *S. frontalis* | JX414588 | DLOOP269 | NWA | Viricel et al.,2014 |
| *S. frontalis* | JX414589 | DLOOP270 | NWA | Viricel et al.,2014 |
| *S. frontalis* | JX414590 | DLOOP271 | NWA | Viricel et al.,2014 |
| *S. frontalis* | JX414591 | DLOOP272 | NWA | Viricel et al.,2014 |
| *S. frontalis* | JX414592 | DLOOP273 | NWA | Viricel et al.,2014 |
| *S. frontalis* | JX414593 | DLOOP274 | NWA | Viricel et al.,2014 |
| *S. frontalis* | JX414594 | DLOOP275 | NWA | Viricel et al.,2014 |
| *S. frontalis* | JX414595 | DLOOP276 | NWA | Viricel et al.,2014 |
| *S. frontalis* | JX414596 | DLOOP277 | NWA | Viricel et al.,2014 |
| *S. longirostris* | MK184993 | DLOOP278 | SWA (Brazil) | Faria et al., 2020 |
| *S. longirostris* | MK184992 | DLOOP279 | SWA (Brazil) | Faria et al., 2020 |
| *S. longirostris* | MT906136 | DLOOP280 | SWA (Brazil) | This study |
| *S. longirostris* | MT906137 | DLOOP281 | SWA (Brazil) | This study |
| *S. longirostris* | MT906138 | DLOOP282 | SWA (Brazil) | This study |
| *S. longirostris* | MT906139 | DLOOP283 | SWA (Brazil) | This study |
| *S. longirostris* | MT906140 | DLOOP284 | SWA (Brazil) | This study |
| *S. longirostris* | MT906141 | DLOOP285 | SWA (Brazil) | This study |
| *S. longirostris* | MT906142 | DLOOP286 | SWA (Brazil) | This study |
| *S. longirostris* | MT906143 | DLOOP287 | SWA (Brazil) | This study |
| *S. longirostris* | MT906144 | DLOOP288 | SWA (Brazil) | This study |
| *S. longirostris* | MT906145 | DLOOP289 | SWA (Brazil) | This study |
| *S. longirostris* | MT906146 | DLOOP290 | SWA (Brazil) | This study |
| *S. longirostris* | MT906147 | DLOOP291 | SWA (Brazil) | This study |
| *S. longirostris* | DQ845444 | DLOOP292 | NWA | Kingston et al.,2009 |
| *S. longirostris* | DQ845445 | DLOOP282 | NWA | Kingston et al.,2009 |
| *S. longirostris* | EU121117 | DLOOP293 | SEPO | Caballero et al.,2008 |
| *S. longirostris* | GQ504125 | DLOOP294 | EA | Kingston et al.,2009 |
| *S. longirostris* | GQ504165 | DLOOP295 | NWA | Kingston et al.,2009 |
| *S. longirostris* | GQ504166 | DLOOP296 | NWA | Kingston et al.,2009 |
| *S. longirostris* | GQ504167 | DLOOP297 | NWA | Kingston et al.,2009 |
| *S. longirostris* | GQ504168 | DLOOP298 | NWA | Kingston et al.,2009 |
| *S. longirostris* | GQ504169 | DLOOP253 | NWA | Kingston et al.,2009 |
| *S. longirostris* | GU253256 | DLOOP299 | NEP | Andrews et al.,2010 |
| *S. longirostris* | GU253257 | DLOOP300 | NEP | Andrews et al.,2010 |
| *S. longirostris* | GU253258 | DLOOP301 | NEP | Andrews et al.,2010 |
| *S. longirostris* | GU253259 | DLOOP302 | NEP | Andrews et al.,2010 |
| *S. longirostris* | GU253260 | DLOOP303 | NEP, SWP | Andrews et al.,2010 |
| *S. longirostris* | GU253261 | DLOOP304 | NEP | Andrews et al.,2010 |
| *S. longirostris* | GU253262 | DLOOP305 | NEP, SWP | Andrews et al.,2010 |
| *S. longirostris* | GU253263 | DLOOP306 | NEP, SWP | Andrews et al.,2010 |
| *S. longirostris* | GU253264 | DLOOP307 | NEP | Andrews et al.,2010 |
| *S. longirostris* | GU253265 | DLOOP308 | NEP | Andrews et al.,2010 |
| *S. longirostris* | GU253266 | DLOOP309 | NEP | Andrews et al.,2010 |
| *S. longirostris* | GU253267 | DLOOP310 | NEP | Andrews et al.,2010 |
| *S. longirostris* | GU253268 | DLOOP289 | NEP | Andrews et al.,2010 |
| *S. longirostris* | GU253269 | DLOOP311 | NEP | Andrews et al.,2010 |
| *S. longirostris* | GU253270 | DLOOP312 | NEP | Andrews et al.,2010 |
| *S. longirostris* | GU253271 | DLOOP313 | NEP | Andrews et al.,2010 |
| *S. longirostris* | GU253272 | DLOOP314 | NEP | Andrews et al.,2010 |
| *S. longirostris* | GU253273 | DLOOP315 | NEP | Andrews et al.,2010 |
| *S. longirostris* | GU253274 | DLOOP316 | NEP | Andrews et al.,2010 |
| *S. longirostris* | GU253275 | DLOOP317 | SWP | Andrews et al.,2010 |
| *S. longirostris* | GU253276 | DLOOP318 | SWP | Andrews et al.,2010 |
| *S. longirostris* | GU253277 | DLOOP319 | SWP | Andrews et al.,2010 |
| *S. longirostris* | GU253278 | DLOOP320 | SWP | Andrews et al.,2010 |
| *S. longirostris* | GU253279 | DLOOP321 | SWP | Andrews et al.,2010 |
| *S. longirostris* | GU253280 | DLOOP322 | SWP | Andrews et al.,2010 |
| *S. longirostris* | GU253281 | DLOOP323 | SWP | Andrews et al.,2010 |
| *S. longirostris* | GU253282 | DLOOP324 | SWP | Andrews et al.,2010 |
| *S. longirostris* | GU253283 | DLOOP325 | SWP | Andrews et al.,2010 |
| *S. longirostris* | GU253284 | DLOOP326 | SWP | Andrews et al.,2010 |
| *S. longirostris* | KC160997 | DLOOP312 | NEP | Andrews et al.,2013 |
| *S. longirostris* | KC160998 | DLOOP299 | NEP | Andrews et al.,2013 |
| *S. longirostris* | KC160999 | DLOOP306 | NEP, NWP, SEP | Andrews et al.,2013 |
| *S. longirostris* | KC161000 | DLOOP327 | EP | Andrews et al.,2013 |
| *S. longirostris* | KC161001 | DLOOP328 | EP | Andrews et al.,2013 |
| *S. longirostris* | KC161002 | DLOOP329 | EP | Andrews et al.,2013 |
| *S. longirostris* | KC161003 | DLOOP330 | EP | Andrews et al.,2013 |
| *S. longirostris* | KC161004 | DLOOP331 | EP | Andrews et al.,2013 |
| *S. longirostris* | KC161005 | DLOOP332 | EP | Andrews et al.,2013 |
| *S. longirostris* | KC161006 | DLOOP333 | EP | Andrews et al.,2013 |
| *S. longirostris* | KC161007 | DLOOP334 | EP | Andrews et al.,2013 |
| *S. longirostris* | KC161008 | DLOOP335 | EP | Andrews et al.,2013 |
| *S. longirostris* | KC161009 | DLOOP329 | EP | Andrews et al.,2013 |
| *S. longirostris* | KC161010 | DLOOP336 | EP | Andrews et al.,2013 |
| *S. longirostris* | KC161011 | DLOOP337 | EP | Andrews et al.,2013 |
| *S. longirostris* | KC161012 | DLOOP338 | EP | Andrews et al.,2013 |
| *S. longirostris* | KC161013 | DLOOP339 | EP | Andrews et al.,2013 |
| *S. longirostris* | KC161014 | DLOOP340 | EP | Andrews et al.,2013 |
| *S. longirostris* | KC161015 | DLOOP341 | EP | Andrews et al.,2013 |
| *S. longirostris* | KC161016 | DLOOP342 | IP | Andrews et al.,2013 |
| *S. longirostris* | KC161017 | DLOOP343 | EP | Andrews et al.,2013 |
| *S. longirostris* | KC161018 | DLOOP314 | NEP | Andrews et al.,2013 |
| *S. longirostris* | KC161019 | DLOOP344 | EP | Andrews et al.,2013 |
| *S. longirostris* | KC161020 | DLOOP345 | EP | Andrews et al.,2013 |
| *S. longirostris* | KC161021 | DLOOP346 | SWI | Andrews et al.,2013 |
| *S. longirostris* | KC161022 | DLOOP347 | EP | Andrews et al.,2013 |
| *S. longirostris* | KC161023 | DLOOP348 | EPO | Andrews et al.,2013 |
| *S. longirostris* | KC161024 | DLOOP349 | EP | Andrews et al.,2013 |
| *S. longirostris* | KC161025 | DLOOP350 | EP | Andrews et al.,2013 |
| *S. longirostris* | KC161026 | DLOOP351 | EP | Andrews et al.,2013 |
| *S. longirostris* | KC161027 | DLOOP352 | EP | Andrews et al.,2013 |
| *S. longirostris* | KC161028 | DLOOP353 | EP | Andrews et al.,2013 |
| *S. longirostris* | KC161029 | DLOOP354 | EP | Andrews et al.,2013 |
| *S. longirostris* | KC161030 | DLOOP355 | EP | Andrews et al.,2013 |
| *S. longirostris* | KC161031 | DLOOP356 | EP | Andrews et al.,2013 |
| *S. longirostris* | KC161032 | DLOOP321 | EP | Andrews et al.,2013 |
| *S. longirostris* | KC161033 | DLOOP357 | EP | Andrews et al.,2013 |
| *S. longirostris* | KC161034 | DLOOP358 | EP | Andrews et al.,2013 |
| *S. longirostris* | KC161035 | DLOOP340 | EP | Andrews et al.,2013 |
| *S. longirostris* | KC161036 | DLOOP341 | EP | Andrews et al.,2013 |
| *S. longirostris* | KC161037 | DLOOP283 | NEI, SWI, IP | Andrews et al.,2013 |
| *S. longirostris* | KC161038 | DLOOP303 | NEP, SEP, SWP | Andrews et al.,2013 |
| *S. longirostris* | KC161039 | DLOOP301 | NEP | Andrews et al.,2013 |
| *S. longirostris* | KC161040 | DLOOP300 | NEP | Andrews et al.,2013 |
| *S. longirostris* | KC161041 | DLOOP296 | NWA | Andrews et al.,2013 |
| *S. longirostris* | KC161042 | DLOOP282 | NWA | Andrews et al.,2013 |
| *S. longirostris* | KC161043 | DLOOP359 | NWP | Andrews et al.,2013 |
| *S. longirostris* | KC161044 | DLOOP360 | NWP | Andrews et al.,2013 |
| *S. longirostris* | KC161045 | DLOOP361 | NWP | Andrews et al.,2013 |
| *S. longirostris* | KC161046 | DLOOP362 | NWP | Andrews et al.,2013 |
| *S. longirostris* | KC161047 | DLOOP363 | IP | Andrews et al.,2013 |
| *S. longirostris* | KC161048 | DLOOP364 | IP | Andrews et al.,2013 |
| *S. longirostris* | KC161049 | DLOOP365 | IP | Andrews et al.,2013 |
| *S. longirostris* | KC161050 | DLOOP366 | IP | Andrews et al.,2013 |
| *S. longirostris* | KC161051 | DLOOP367 | NEI | Andrews et al.,2013 |
| *S. longirostris* | KC161052 | DLOOP368 | NEI | Andrews et al.,2013 |
| *S. longirostris* | KC161053 | DLOOP369 | NEI | Andrews et al.,2013 |
| *S. longirostris* | KC161054 | DLOOP304 | NEP | Andrews et al.,2013 |
| *S. longirostris* | KC161055 | DLOOP284 | NWA | Andrews et al.,2013 |
| *S. longirostris* | KC161056 | DLOOP370 | NWA | Andrews et al.,2013 |
| *S. longirostris* | KC161057 | DLOOP307 | NWP, NEP | Andrews et al.,2013 |
| *S. longirostris* | KC161058 | DLOOP308 | NEP, NWP | Andrews et al.,2013 |
| *S. longirostris* | KC161059 | DLOOP371 | NWP | Andrews et al.,2013 |
| *S. longirostris* | KC161060 | DLOOP372 | NWP | Andrews et al.,2013 |
| *S. longirostris* | KC161061 | DLOOP373 | NWP | Andrews et al.,2013 |
| *S. longirostris* | KC161062 | DLOOP374 | NWP | Andrews et al.,2013 |
| *S. longirostris* | KC161063 | DLOOP375 | NWP | Andrews et al.,2013 |
| *S. longirostris* | KC161064 | DLOOP311 | NEP | Andrews et al.,2013 |
| *S. longirostris* | KC161065 | DLOOP376 | NEP | Andrews et al.,2013 |
| *S. longirostris* | KC161066 | DLOOP324 | NEP, SEP, SWP | Andrews et al.,2013 |
| *S. longirostris* | KC161067 | DLOOP323 | NEP, SEP, SWP | Andrews et al.,2013 |
| *S. longirostris* | KC161068 | DLOOP317 | SWP | Andrews et al.,2013 |
| *S. longirostris* | KC161069 | DLOOP318 | SWP | Andrews et al.,2013 |
| *S. longirostris* | KC161070 | DLOOP319 | SWP | Andrews et al.,2013 |
| *S. longirostris* | KC161071 | DLOOP305 | SWP | Andrews et al.,2013 |
| *S. longirostris* | KC161072 | DLOOP320 | SWP, SEP | Andrews et al.,2013 |
| *S. longirostris* | KC161073 | DLOOP321 | SWP, SEP | Andrews et al.,2013 |
| *S. longirostris* | KC161074 | DLOOP322 | SWP | Andrews et al.,2013 |
| *S. longirostris* | KC161075 | DLOOP325 | SWP | Andrews et al.,2013 |
| *S. longirostris* | KC161076 | DLOOP326 | SWP | Andrews et al.,2013 |
| *S. longirostris* | KC161077 | DLOOP313 | NWP | Andrews et al.,2013 |
| *S. longirostris* | KC161078 | DLOOP377 | EP | Andrews et al.,2013 |
| *S. longirostris* | KC161079 | DLOOP378 | EP | Andrews et al.,2013 |
| *S. longirostris* | KC161080 | DLOOP379 | EP | Andrews et al.,2013 |
| *S. longirostris* | KC161081 | DLOOP380 | EP | Andrews et al.,2013 |
| *S. longirostris* | KC161082 | DLOOP381 | EP | Andrews et al.,2013 |
| *S. longirostris* | KC161083 | DLOOP382 | EP | Andrews et al.,2013 |
| *S. longirostris* | KC161084 | DLOOP383 | EP | Andrews et al.,2013 |
| *S. longirostris* | KC161085 | DLOOP346 | EP | Andrews et al.,2013 |
| *S. longirostris* | KC161086 | DLOOP384 | EP | Andrews et al.,2013 |
| *S. longirostris* | KC161087 | DLOOP346 | EP | Andrews et al.,2013 |
| *S. longirostris* | KC161088 | DLOOP385 | EP | Andrews et al.,2013 |
| *S. longirostris* | KC161089 | DLOOP386 | EP | Andrews et al.,2013 |
| *S. longirostris* | KC161090 | DLOOP387 | EP | Andrews et al.,2013 |
| *S. longirostris* | KC161091 | DLOOP388 | EP | Andrews et al.,2013 |
| *S. longirostris* | KC161092 | DLOOP389 | EP | Andrews et al.,2013 |
| *S. longirostris* | KC161093 | DLOOP390 | IP | Andrews et al.,2013 |
| *S. longirostris* | KC161094 | DLOOP391 | IP | Andrews et al.,2013 |
| *S. longirostris* | KC161095 | DLOOP392 | IP | Andrews et al.,2013 |
| *S. longirostris* | KC161096 | DLOOP393 | NWP | Andrews et al.,2013 |
| *S. longirostris* | KC161097 | DLOOP394 | NWP | Andrews et al.,2013 |
| *S. longirostris* | KC161098 | DLOOP355 | NWP | Andrews et al.,2013 |
| *S. longirostris* | KC161099 | DLOOP395 | EP | Andrews et al.,2013 |
| *S. longirostris* | KC161100 | DLOOP396 | EP | Andrews et al.,2013 |
| *S. longirostris* | KC161101 | DLOOP397 | EP | Andrews et al.,2013 |
| *S. longirostris* | KC161102 | DLOOP398 | EP | Andrews et al.,2013 |
| *S. longirostris* | KC161103 | DLOOP399 | EP | Andrews et al.,2013 |
| *S. longirostris* | KC161104 | DLOOP400 | EP | Andrews et al.,2013 |
| *S. longirostris* | KC161105 | DLOOP401 | EP | Andrews et al.,2013 |
| *S. longirostris* | KC161106 | DLOOP357 | EP | Andrews et al.,2013 |
| *S. longirostris* | KC161107 | DLOOP313 | EP | Andrews et al.,2013 |
| *S. longirostris* | KC161108 | DLOOP402 | EP | Andrews et al.,2013 |
| *S. longirostris* | KC161109 | DLOOP314 | EP | Andrews et al.,2013 |
| *S. longirostris* | KC161110 | DLOOP403 | EP | Andrews et al.,2013 |
| *S. longirostris* | KC161111 | DLOOP404 | EP | Andrews et al.,2013 |
| *S. longirostris* | KC161112 | DLOOP405 | EP | Andrews et al.,2013 |
| *S. longirostris* | KC161113 | DLOOP406 | SWI | Andrews et al.,2013 |
| *S. longirostris* | KC161114 | DLOOP280 | SWI | Andrews et al.,2013 |
| *S. longirostris* | KC161115 | DLOOP407 | SWI | Andrews et al.,2013 |
| *S. longirostris* | KC161116 | DLOOP408 | SEP | Andrews et al.,2013 |
| *S. longirostris* | KC161117 | DLOOP310 | SEP | Andrews et al.,2013 |
| *S. longirostris* | KC161118 | DLOOP293 | SEP | Andrews et al.,2013 |
| *S. longirostris* | KC161119 | DLOOP409 | SEP | Andrews et al.,2013 |
| *S. longirostris* | KC161120 | DLOOP410 | SEP | Andrews et al.,2013 |
| *S. longirostris* | KC161121 | DLOOP323 | SEP | Andrews et al.,2013 |
| *S. longirostris* | KC161122 | DLOOP411 | SEP | Andrews et al.,2013 |
| *S. longirostris* | KC161123 | DLOOP412 | SEP | Andrews et al.,2013 |
| *S. longirostris* | KC161124 | DLOOP393 | SEP | Andrews et al.,2013 |
| *S. longirostris* | KC161125 | DLOOP413 | SEP | Andrews et al.,2013 |
| *S. longirostris* | KX905105 | DLOOP406 | SWI | Viricel et al.,2016 |
| *S. longirostris* | KX905106 | DLOOP414 | SWI | Viricel et al.,2016 |
| *S. longirostris* | KX905107 | DLOOP415 | SWI | Viricel et al.,2016 |
| *S. longirostris* | KX905108 | DLOOP314 | SWI | Viricel et al.,2016 |
| *S. longirostris* | KX905109 | DLOOP283 | SWI | Viricel et al.,2016 |
| *S. longirostris* | KX905110 | DLOOP416 | SWI | Viricel et al.,2016 |
| *S. longirostris* | KX905111 | DLOOP417 | SWI | Viricel et al.,2016 |
| *S. longirostris* | KX905112 | DLOOP418 | SWI | Viricel et al.,2016 |
| *S. longirostris* | KX905113 | DLOOP395 | SWI | Viricel et al.,2016 |
| *S. longirostris* | KX905114 | DLOOP419 | SWI | Viricel et al.,2016 |
| *S. longirostris* | KX905115 | DLOOP420 | SWI | Viricel et al.,2016 |
| *S. longirostris* | KX905116 | DLOOP421 | SWI | Viricel et al.,2016 |
| *S. longirostris* | KX905117 | DLOOP346 | SWI | Viricel et al.,2016 |
| *S. longirostris* | KX905118 | DLOOP422 | SWI | Viricel et al.,2016 |
| *S. longirostris* | KX905119 | DLOOP423 | SWI | Viricel et al.,2016 |
| *S. longirostris* | KX905120 | DLOOP406 | SWI | Viricel et al.,2016 |
| *S. longirostris* | KX905121 | DLOOP280 | SWI | Viricel et al.,2016 |
| *S. longirostris* | KX905122 | DLOOP424 | SWI | Viricel et al.,2016 |
| *S. longirostris* | KX905123 | DLOOP395 | SWI | Viricel et al.,2016 |
| *S. longirostris* | KX905124 | DLOOP314 | SWI | Viricel et al.,2016 |
| *S. longirostris* | KX905125 | DLOOP345 | SWI | Viricel et al.,2016 |
| *S. longirostris* | KX905126 | DLOOP425 | SWI | Viricel et al.,2016 |
| *S. longirostris* | KX905127 | DLOOP426 | SWI | Viricel et al.,2016 |
| *S. longirostris* | KX905128 | DLOOP427 | SWI | Viricel et al.,2016 |
| *S. longirostris* | KX905129 | DLOOP428 | SWI | Viricel et al.,2016 |
| *S. longirostris* | KX905130 | DLOOP429 | SWI | Viricel et al.,2016 |
| *S. longirostris* | KX905131 | DLOOP314 | SWI | Viricel et al.,2016 |
| *S. longirostris* | KX905132 | DLOOP279 | SWI | Viricel et al.,2016 |
| *S. longirostris* | KY457781 | DLOOP359 | NWP | Martien et al.,2014 |
| *S. longirostris* | KY457782 | DLOOP430 | NWP | Martien et al.,2014 |
| *S. longirostris* | KY457783 | DLOOP360 | NWP | Martien et al.,2014 |
| *S. longirostris* | KY457784 | DLOOP306 | NWP | Martien et al.,2014 |
| *S. longirostris* | KY457785 | DLOOP362 | NWP | Martien et al.,2014 |
| *S. longirostris* | KY457786 | DLOOP307 | NWP | Martien et al.,2014 |
| *S. longirostris* | KY457787 | DLOOP431 | NWP | Martien et al.,2014 |
| *S. longirostris* | KY457788 | DLOOP304 | NWP | Martien et al.,2014 |
| *S. longirostris* | KY457789 | DLOOP432 | NWP | Martien et al.,2014 |
| *S. longirostris* | KY457790 | DLOOP371 | NWP | Martien et al.,2014 |
| *S. longirostris* | KY457791 | DLOOP301 | NWP | Martien et al.,2014 |
| *S. longirostris* | KY457792 | DLOOP310 | NWP | Martien et al.,2014 |
| *S. longirostris* | KY457793 | DLOOP313 | NWP | Martien et al.,2014 |
| *S. longirostris* | KY457794 | DLOOP433 | NWP | Martien et al.,2014 |
| *S. longirostris* | KY457795 | DLOOP361 | NWP | Martien et al.,2014 |
| *S. longirostris* | KY457796 | DLOOP434 | NWP | Martien et al.,2014 |
| *S. longirostris* | KY457797 | DLOOP323 | NWP | Martien et al.,2014 |
| *S. longirostris* | KY457798 | DLOOP414 | NWP | Martien et al.,2014 |
| *S. longirostris* | KY457799 | DLOOP435 | NWP | Martien et al.,2014 |
| *S. longirostris* | KY457800 | DLOOP436 | NWP | Martien et al.,2014 |
| *S. longirostris* | KY457801 | DLOOP437 | NWP | Martien et al.,2014 |
| *S. longirostris* | KY457802 | DLOOP311 | NWP | Martien et al.,2014 |
| *S. longirostris* | KY457803 | DLOOP314 | NWP | Martien et al.,2014 |
| *S. longirostris* | KY457804 | DLOOP309 | NWP | Martien et al.,2014 |
| *S. longirostris* | EF558737 | DLOOP410 | SEP | Oremus et al.,2007 |
| *S. longirostris* | EF558738 | DLOOP408 | SEP | Oremus et al.,2007 |
| *S. longirostris* | EF558739 | DLOOP310 | SEP | Oremus et al.,2007 |
| *S. longirostris* | EF558740 | DLOOP293 | SEP | Oremus et al.,2007 |
| *S. longirostris* | EF558741 | DLOOP409 | SEP | Oremus et al.,2007 |
| *S. longirostris* | EF558742 | DLOOP323 | SEP | Oremus et al.,2007 |
| *S. longirostris* | EF558743 | DLOOP321 | SEP | Oremus et al.,2007 |
| *S. longirostris* | EF558744 | DLOOP364 | SEP | Oremus et al.,2007 |
| *S. longirostris* | EF558745 | DLOOP320 | SEP | Oremus et al.,2007 |
| *S. longirostris* | EF558746 | DLOOP306 | SEP | Oremus et al.,2007 |
| *S. longirostris* | EF558747 | DLOOP324 | SEP | Oremus et al.,2007 |
| *S. longirostris* | EF558748 | DLOOP323 | SEP | Oremus et al.,2007 |
| *S. longirostris* | EF558749 | DLOOP412 | SEP | Oremus et al.,2007 |
| *S. longirostris* | EF558750 | DLOOP411 | SEP | Oremus et al.,2007 |
| *S. longirostris* | EF558751 | DLOOP308 | SEP | Oremus et al.,2007 |
| *S. longirostris* | EF558752 | DLOOP303 | SEP | Oremus et al.,2007 |
| *S. longirostris* | EF558753 | DLOOP393 | SEP | Oremus et al.,2007 |
| *S. longirostris* | EF558754 | DLOOP323 | SEP | Oremus et al.,2007 |
| *S. longirostris* | EF558755 | DLOOP322 | SEP | Oremus et al.,2007 |
| *S. longirostris* | EF558756 | DLOOP438 | SEP | Oremus et al.,2007 |
| *S. longirostris* | EF558757 | DLOOP304 | SEP | Oremus et al.,2007 |
| *S. longirostris* | EF558758 | DLOOP393 | SEP | Oremus et al.,2007 |
| *S. longirostris* | EF558759 | DLOOP439 | SEP | Oremus et al.,2007 |
| *S. longirostris* | EF558760 | DLOOP350 | SEP | Oremus et al.,2007 |
| *S. longirostris* | EF558761 | DLOOP440 | SEP | Oremus et al.,2007 |
| *S. longirostris* | EF558762 | DLOOP441 | SEP | Oremus et al.,2007 |
| *S. longirostris* | EF558763 | DLOOP434 | SEP | Oremus et al.,2007 |
| *S. longirostris* | EF558764 | DLOOP442 | SEP | Oremus et al.,2007 |
| *S. longirostris* | EF558765 | DLOOP314 | SEP | Oremus et al.,2007 |
| *S. longirostris* | EF558766 | DLOOP443 | SEP | Oremus et al.,2007 |
| *S. longirostris* | EF558767 | DLOOP323 | SEP | Oremus et al.,2007 |
| *S. longirostris* | KP756642 | DLOOP444 | SWP | Oremus et al.,2015 |
| *S. longirostris* | KP756643 | DLOOP308 | SWP | Oremus et al.,2015 |
| *S. longirostris* | KP756644 | DLOOP408 | SWP | Oremus et al.,2015 |
| **Species** | **GenBank** | **Haplotype** | **Localization** | **Source** |
| ***S. attenuata*** | MT906078 | CYTB1 | SWA (Brazil) | This study |
| *S. attenuata* | MT906079 | CYTB2 | SWA (Brazil) | This study |
| *S. attenuata* | MT906080 | CYTB3 | SWA (Brazil) | This study |
| *S. attenuata* | EF438304 | CYTB1 | IN | Jayasankar et al.,2009 |
| *S. attenuata* | AF084096 | CYTB3 | EP | Leduc et al.,1999 |
| *S. attenuata* | AF084097 | CYTB4 | NWA | Leduc et al.,1999 |
| ***S. clymene*** | MT906081 | CYTB5 | SWA (Brazil) | This study |
| *S. clymene* | MT906108 | CYTB6 | SWA (Brazil) | This study |
| *S. clymene* | MT906082 | CYTB7 | SWA (Brazil) | This study |
| *S. clymene* | MT906083 | CYTB8 | SWA (Brazil) | This study |
| *S. clymene* | MT906084 | CYTB9 | SWA (Brazil) | This study |
| *S. clymene* | MT906085 | CYTB10 | SWA (Brazil) | This study |
| *S. clymene* | EU517711 | CYTB11 | NWA | Viricel et al.,2012 |
| *S. clymene* | EU517712 | CYTB12 | NWA | Viricel et al.,2012 |
| ***S. coeruleoalba*** | MT906086 | CYTB10 | SWA (Brazil) | This study |
| *S. coeruleoalba* | MT906087 | CYTB13 | SWA (Brazil) | This study |
| *S. coeruleoalba* | MT906088 | CYTB14 | SWA (Brazil) | This study |
| *S. coeruleoalba* | MT906089 | CYTB15 | SWA (Brazil) | This study |
| *S. coeruleoalba* | MT906090 | CYTB16 | SWA (Brazil) | This study |
| *S. coeruleoalba* | MT906091 | CYTB17 | SWA (Brazil) | This study |
| *S. coeruleoalba* | MT906092 | CYTB18 | SWA (Brazil) | This study |
| *S. coeruleoalba* | MT906093 | CYTB19 | SWA (Brazil) | This study |
| *S. coeruleoalba* | EU580088 | CYTB20 | NWA | Viricel et al.,2012 |
| *S. coeruleoalba* | AF084082 | CYTB21 | NEA | Leduc et al.,1999 |
| ***S. frontalis*** | MT906094 | CYTB22 | SWA (Brazil) | This study |
| *S. frontalis* | MT906095 | CYTB23 | SWA (Brazil) | This study |
| *S. frontalis* | MT906096 | CYTB24 | SWA (Brazil) | This study |
| *S. frontalis* | MT906097 | CYTB25 | SWA (Brazil) | This study |
| *S. frontalis* | MT906098 | CYTB26 | SWA (Brazil) | This study |
| *S. frontalis* | MT906099 | CYTB27 | SWA (Brazil) | This study |
| *S. frontalis* | EU121092 | CYTB28 | NWA | Leduc et al.,1999 |
| *S. frontalis* | EU517713 | CYTB23 | NWA | Leduc et al.,1999 |
| *S. frontalis* | EU517714 | CYTB29 | NWA | Caballero et al.,2008 |
| ***S. longirostris*** | MT906100 | CYTB30 | SWA (Brazil) | This study |
| *S. longirostris* | MT906101 | CYTB31 | SWA (Brazil) | This study |
| *S. longirostris* | MT906102 | CYTB32 | SWA (Brazil) | This study |
| *S. longirostris* | MT906103 | CYTB33 | SWA (Brazil) | This study |
| *S. longirostris* | MT906104 | CYTB34 | SWA (Brazil) | This study |
| *S. longirostris* | MT906105 | CYTB35 | SWA (Brazil) | This study |
| *S. longirostris* | MT906106 | CYTB36 | SWA (Brazil) | This study |
| *S. longirostris* | MT906107 | CYTB37 | SWA (Brazil) | This study |
| *S. longirostris* | AF084100 | CYTB38 | NWA | Leduc et al.,1999 |
| *S. longirostris* | AF084101 | CYTB39 | EP | Leduc et al.,1999 |
| *S. longirostris* | AF084102 | CYTB40 | NWP | Leduc et al.,1999 |
| *S. longirostris* | AF084103 | CYTB41 | IN | Leduc et al.,1999 |
| *S. longirostris* | DQ232770 | CYTB42 | IN | Jayasankar et al.,2008 |
| *S. longirostris* | DQ270182 | CYTB35 | IN | Jayasankar et al.,2008 |
| *S. longirostris* | EF057433 | CYTB35 | IN | Jayasankar et al.,2008 |
| *S. longirostris* | EF057434 | CYTB43 | IN | Jayasankar et al.,2008 |
| *S. longirostris* | EF057436 | CYTB44 | IN | Jayasankar et al.,2008 |
| *S. longirostris* | EF057437 | CYTB45 | IN | Jayasankar et al.,2008 |
| *S. longirostris* | EF057438 | CYTB45 | IN | Jayasankar et al.,2008 |
| *S. longirostris* | EF203445 | CYTB41 | IN | Jayasankar et al.,2008 |
| *S. longirostris* | EF203446 | CYTB31 | IN | Jayasankar et al.,2008 |
| *S. longirostris* | EF203447 | CYTB46 | IN | Jayasankar et al.,2008 |
| *S. longirostris* | EF203448 | CYTB34 | IN | Jayasankar et al.,2008 |
| *S. longirostris* | EF203449 | CYTB31 | IN | Jayasankar et al.,2008 |
| *S. longirostris* | EF203450 | CYTB34 | IN | Jayasankar et al.,2008 |
| *S. longirostris* | EF446613 | CYTB44 | IN | Jayasankar et al.,2008 |
| *S. longirostris* | EF446614 | CYTB31 | IN | Jayasankar et al.,2008 |
| *S. longirostris* | EU204619 | CYTB34 | IN | Jayasankar et al.,2008 |
| *S. longirostris* | EU517703 | CYTB35 | NWA | Viricel et al.,2012 |
| *S. longirostris* | EU517715 | CYTB47 | NWA | Viricel et al.,2012 |
| *S. longirostris* | KC161126 | CYTB48 | NEP | Andrews et al.,2013 |
| *S. longirostris* | KC161127 | CYTB35 | EP, NEP, NWP, SEP, SWP | Andrews et al.,2013 |
| *S. longirostris* | KC161128 | CYTB49 | EP, NEP, NWP, SEP, SWP, | Andrews et al.,2013 |
| *S. longirostris* | KC161129 | CYTB34 | EP, NEP, NWA, NWP, NEI, NWA, SEP, SWP, IP | Andrews et al.,2013 |
| *S. longirostris* | KC161130 | CYTB31 | EP, NWP, NEP, SEP, SWP, NWP, IP | Andrews et al.,2013 |
| *S. longirostris* | KC161131 | CYTB50 | EP | Andrews et al.,2013 |
| *S. longirostris* | KC161132 | CYTB31 | EP | Andrews et al.,2013 |
| *S. longirostris* | KC161133 | CYTB51 | EP | Andrews et al.,2013 |
| *S. longirostris* | KC161134 | CYTB34 | EP | Andrews et al.,2013 |
| *S. longirostris* | KC161135 | CYTB52 | EP | Andrews et al.,2013 |
| *S. longirostris* | KC161136 | CYTB53 | EP | Andrews et al.,2013 |
| *S. longirostris* | KC161137 | CYTB31 | EP, NEP, SEP, SWP, IP | Andrews et al.,2013 |
| *S. longirostris* | KC161138 | CYTB54 | EP | Andrews et al.,2013 |
| *S. longirostris* | KC161139 | CYTB55 | EP | Andrews et al.,2013 |
| *S. longirostris* | KC161140 | CYTB56 | EP | Andrews et al.,2013 |
| *S. longirostris* | KC161141 | CYTB57 | EP | Andrews et al.,2013 |
| *S. longirostris* | KC161142 | CYTB31 | EP | Andrews et al.,2013 |
| *S. longirostris* | KC161143 | CYTB41 | EP, NWP, SWP, IP | Andrews et al.,2013 |
| *S. longirostris* | KC161144 | CYTB58 | EP, SEP | Andrews et al.,2013 |
| *S. longirostris* | KC161145 | CYTB59 | EP | Andrews et al.,2013 |
| *S. longirostris* | KC161146 | CYTB60 | EP, NEP | Andrews et al.,2013 |
| *S. longirostris* | KC161147 | CYTB35 | NWA | Andrews et al.,2013 |
| *S. longirostris* | KC161148 | CYTB47 | NWA | Andrews et al.,2013 |
| *S. longirostris* | KC161149 | CYTB61 | NWA | Andrews et al.,2013 |
| *S. longirostris* | KC161150 | CYTB31 | NWPO | Andrews et al.,2013 |
| *S. longirostris* | KC161151 | CYTB62 | IPO | Andrews et al.,2013 |
| *S. longirostris* | KC161152 | CYTB63 | EP, IP, NWP | Andrews et al.,2013 |
| *S. longirostris* | KC161153 | CYTB64 | IP | Andrews et al.,2013 |
| *S. longirostris* | KC161154 | CYTB32 | NEI | Andrews et al.,2013 |
| *S. longirostris* | KC161155 | CYTB35 | NEI | Andrews et al.,2013 |
| *S. longirostris* | KC161156 | CYTB34 | NEI | Andrews et al.,2013 |
| *S. longirostris* | KC161157 | CYTB65 | NEI | Andrews et al.,2013 |
| *S. longirostris* | KC161158 | CYTB52 | NEP, NWP | Andrews et al.,2013 |
| *S. longirostris* | KC161159 | CYTB65 | NEP | Andrews et al.,2013 |
| *S. longirostris* | KC161160 | CYTB31 | NWP | Andrews et al.,2013 |
| *S. longirostris* | KC161161 | CYTB66 | NWP | Andrews et al.,2013 |
| *S. longirostris* | KC161162 | CYTB67 | NWP | Andrews et al.,2013 |
| *S. longirostris* | KC161163 | CYTB68 | NWP | Andrews et al.,2013 |
| *S. longirostris* | KC161164 | CYTB69 | NEP | Andrews et al.,2013 |
| *S. longirostris* | KC161165 | CYTB70 | SEP, SWP | Andrews et al.,2013 |
| *S. longirostris* | KC161166 | CYTB71 | EP, NWP | Andrews et al.,2013 |
| *S. longirostris* | KC161167 | CYTB72 | EP | Andrews et al.,2013 |
| *S. longirostris* | KC161168 | CYTB73 | EP | Andrews et al.,2013 |
| *S. longirostris* | KC161169 | CYTB49 | EP | Andrews et al.,2013 |
| *S. longirostris* | KC161170 | CYTB74 | EP | Andrews et al.,2013 |
| *S. longirostris* | KC161171 | CYTB75 | EP | Andrews et al.,2013 |
| *S. longirostris* | KC161172 | CYTB76 | EP | Andrews et al.,2013 |
| *S. longirostris* | KC161173 | CYTB77 | EP | Andrews et al.,2013 |
| *S. longirostris* | KC161174 | CYTB78 | EP | Andrews et al.,2013 |
| *S. longirostris* | KC161175 | CYTB79 | EP | Andrews et al.,2013 |
| *S. longirostris* | KC161176 | CYTB80 | EP | Andrews et al.,2013 |
| *S. longirostris* | KC161177 | CYTB81 | SWI | Andrews et al.,2013 |
| *S. longirostris* | KC161178 | CYTB31 | SWI | Andrews et al.,2013 |
| *S. longirostris* | KC161179 | CYTB34 | SWI | Andrews et al.,2013 |
| *S. longirostris* | KC161180 | CYTB45 | SWI | Andrews et al.,2013 |
| *S. longirostris* | KC161181 | CYTB34 | SWI | Andrews et al.,2013 |
| *S. longirostris* | KC161182 | CYTB82 | SEP | Andrews et al.,2013 |
| *S. longirostris* | KC161183 | CYTB83 | SEP | Andrews et al.,2013 |
| **Species** | **GenBank** | **Haplotype** | **Localization** | **Source** |
| ***S. attenuata*** | MT906041 | COXI1 | SWA (Brazil) | This study |
| *S. attenuata* | MT906042 | COXI2 | SWA (Brazil) | This study |
| *S. attenuata* | MT906043 | COXI3 | SWA (Brazil) | This study |
| *S. attenuata* | EU496336 | COXI4 | NWA, EP, NWP | Viricel et al.2012 |
| *S. attenuata* | EU496337 | COXI5 | NWA, EP, NWP | Viricel et al.2012 |
| *S. attenuata* | EU496338 | COXI3 | NWA, EP, NWP | Viricel et al.2012 |
| *S. attenuata* | EU496339 | COXI6 | NWA, EP, NWP | Viricel et al.2012 |
| *S. attenuata* | EU496353 | COXI3 | NWA, EP, NWP | Viricel et al.2012 |
| ***S. clymene*** | MT906044 | COXI7 | SWA (Brazil) | This study |
| *S. clymene* | MT906045 | COXI8 | SWA (Brazil) | This study |
| *S. clymene* | MT906046 | COXI9 | SWA (Brazil) | This study |
| *S. clymene* | MT906047 | COXI10 | SWA (Brazil) | This study |
| *S. clymene* | MT906048 | COXI11 | SWA (Brazil) | This study |
| *S. clymene* | MT906049 | COXI12 | SWA (Brazil) | This study |
| *S. clymene* | MT906050 | COXI13 | SWA (Brazil) | This study |
| *S. clymene* | MT906051 | COXI14 | SWA (Brazil) | This study |
| *S. clymene* | MT906052 | COXI15 | SWA (Brazil) | This study |
| *S. clymene* | EU496346 | COXI16 | NWA | Viricel et al.2012 |
| *S. clymene* | EU496347 | COXI17 | NWA | Viricel et al.2012 |
| *S. clymene* | EU496348 | COXI8 | NWA | Viricel et al.2012 |
| ***S. coeruleoalba*** | MT906053 | COXI15 | SWA (Brazil) | This study |
| *S. coeruleoalba* | MT906054 | COXI18 | SWA (Brazil) | This study |
| *S. coeruleoalba* | MT906055 | COXI19 | SWA (Brazil) | This study |
| *S. coeruleoalba* | MT906056 | COXI20 | SWA (Brazil) | This study |
| *S. coeruleoalba* | MT906057 | COXI21 | SWA (Brazil) | This study |
| *S. coeruleoalba* | DQ466000 | COXI18 | NEA | Amaral et al.,2007 |
| *S. coeruleoalba* | DQ466001 | COXI22 | NEA | Amaral et al.,2007 |
| *S. coeruleoalba* | DQ466002 | COXI23 | NEA | Amaral et al.,2007 |
| *S. coeruleoalba* | DQ466003 | COXI24 | NEA | Amaral et al.,2007 |
| *S. coeruleoalba* | DQ466004 | COXI25 | NEA | Amaral et al.,2007 |
| *S. coeruleoalba* | DQ466005 | COXI19 | NEA | Amaral et al.,2007 |
| *S. coeruleoalba* | DQ466006 | COXI26 | NEA | Amaral et al.,2007 |
| *S. coeruleoalba* | DQ466007 | COXI27 | NEA | Amaral et al.,2007 |
| *S. coeruleoalba* | DQ466008 | COXI28 | NEA | Amaral et al.,2007 |
| *S. coeruleoalba* | DQ466009 | COXI19 | NEA | Amaral et al.,2007 |
| *S. coeruleoalba* | EU496341 | COXI29 | NWA, NEA, NWP | Viricel et al.,2012 |
| *S. coeruleoalba* | EU496342 | COXI26 | NWA, NEA, NWP | Viricel et al.,2012 |
| *S. coeruleoalba* | EU496343 | COXI30 | NWA, NEA, NWP | Viricel et al.,2012 |
| *S. coeruleoalba* | EU496344 | COXI32 | NWA, NEA, NWP | Viricel et al.,2012 |
| *S. coeruleoalba* | KF281695 | COXI31 | NEA | Alfonsi et al.,2013 |
| ***S. frontalis*** | MT906058 | COXI33 | SWA (Brazil) | This study |
| *S. frontalis* | MT906059 | COXI34 | SWA (Brazil) | This study |
| *S. frontalis* | MT906060 | COXI35 | SWA (Brazil) | This study |
| *S. frontalis* | MT906061 | COXI36 | SWA (Brazil) | This study |
| *S. frontalis* | MT906062 | COXI37 | SWA (Brazil) | This study |
| *S. frontalis* | EF090645 | COXI36 | NEA | Amaral et al.,2007 |
| *S. frontalis* | EF090646 | COXI38 | NEA | Amaral et al.,2007 |
| *S. frontalis* | KF281696 | COXI39 | NEA | Alfonsi et al.,2013 |
| ***S. longirostris*** | MT906063 | COXI40 | SWA (Brazil) | This study |
| *S. longirostris* | MT906064 | COXI41 | SWA (Brazil) | This study |
| *S. longirostris* | MT906065 | COXI42 | SWA (Brazil) | This study |
| *S. longirostris* | MT906066 | COXI43 | SWA (Brazil) | This study |
| *S. longirostris* | MT906067 | COXI44 | SWA (Brazil) | This study |
| *S. longirostris* | MT906068 | COXI45 | SWA (Brazil) | This study |
| *S. longirostris* | MT906069 | COXI46 | SWA (Brazil) | This study |
| *S. longirostris* | MT906070 | COXI47 | SWA (Brazil) | This study |
| *S. longirostris* | MT906071 | COXI48 | SWA (Brazil) | This study |
| *S. longirostris* | MT906072 | COXI49 | SWA (Brazil) | This study |
| *S. longirostris* | MT906073 | COXI50 | SWA (Brazil) | This study |
| *S. longirostris* | MT906074 | COXI51 | SWA (Brazil) | This study |
| *S. longirostris* | MT906075 | COXI52 | SWA (Brazil) | This study |
| *S. longirostris* | MT906076 | COXI53 | SWA (Brazil) | This study |
| *S. longirostris* | MT906077 | COXI54 | SWA (Brazil) | This study |
| *S. longirostris* | EU496331 | COXI55 | NWA, ETP | Viricel et al.2012 |
| *S. longirostris* | EU496332 | COXI56 | NWA, ETP | Viricel et al.2012 |
| *S. longirostris* | EU496333 | COXI57 | NWA, ETP | Viricel et al.2012 |
| *S. longirostris* | EU496334 | COXI50 | NWA, ETP | Viricel et al.2012 |
| *S. longirostris* | EU496335 | COXI50 | NWA, ETP | Viricel et al.2012 |
