# Supplementary material for: Mitochondrial diversity and inter-specific phylogeny among dolphins of the genus Stenella in the Southwest Atlantic Ocean
Source: PLoS One. 2022 Jul 14;17(7):e0270690. doi: 10.1371/journal.pone.0270690 (PMC9282552; doi:10.1371/journal.pone.0270690)
Supplement: S3 Table — Sample size (N), number of haplotypes (Nh), polymorphic sites (Ps), haplotype diversity (h), and nucleotide diversity (π). Bp means base pair length. (DOCX) [file pone.0270690.s010.docx]

**S3** **Table Genetic diversity values of *Stenella* species from Brazilian waters for mtDNA control region (Dloop), cytochrome b (Cyt b), and cytochrome oxidase subunit I (Cox I).** Sample size (N), number of haplotypes (Nh), polymorphic sites (Ps), haplotype diversity (h), and nucleotide diversity (π). Bp means base pair length**.**

| **D-loop (310 bp)** | | | | | |
| --- | --- | --- | --- | --- | --- |
|  | N | Nh | Ps | h | *π* |
| *S. attenuata* | 4 | 3 | 11 | 0.833 | 0.020 |
| *S. clymene* | 14 | 12 | 22 | 0.987 | 0.021 |
| *S. coeruleoalba* | 8 | 9 | 25 | 1.00 | 0.027 |
| *S. frontalis* | 14 | 4 | 7 | 0.780 | 0.009 |
| *S. longirostris* | 40 | 14 | 23 | 0.788 | 0.013 |
| **Cyt b (585 bp)** | | | | | |
|  | N | Nh | Ps | h | *π* |
| *S. attenuata* | 4 | 3 | 11 | 0.833 | 0.009 |
| *S. clymene* | 14 | 9 | 18 | 0.935 | 0.007 |
| *S. coeruleoalba* | 8 | 8 | 28 | 0.972 | 0.013 |
| *S. frontalis* | 14 | 6 | 10 | 0.835 | 0.005 |
| *S. longirostris* | 40 | 15 | 17 | 0.769 | 0.006 |
| **Cox I (621 bp)** | | | | | |
|  | N | Nh | Ps | h | *π* |
| *S. attenuata* | 4 | 3 | 22 | 0.8333 | 0.017982 |
| *S. clymene* | 14 | 9 | 43 | 0.9231 | 0.019695 |
| *S. coeruleoalba* | 8 | 5 | 16 | 0.8611 | 0.008678 |
| *S. frontalis* | 14 | 5 | 13 | 0.7692 | 0.006388 |
| *S. longirostris* | 40 | 15 | 14 | 0.8987 | 0.005413 |
