# Supplementary material for: Mitochondrial diversity and inter-specific phylogeny among dolphins of the genus Stenella in the Southwest Atlantic Ocean
Source: PLoS One. 2022 Jul 14;17(7):e0270690. doi: 10.1371/journal.pone.0270690 (PMC9282552; doi:10.1371/journal.pone.0270690)
Supplement: S4 Table — Genetic distance values (%) are bellow diagonal, and, standard errors (SEs) are upper diagonal. (DOCX) [file pone.0270690.s011.docx]

**S4 Table Genetic distances between *Stenella* species from Brazilian waters for mtDNA control region (Dloop), cytochrome b (Cyt b), and cytochrome oxidase subunit I (Cox I).** Genetic distance values (%) are bellow diagonal, and, standard errors (SEs) are upper diagonal.

| **Dloop (310 bp)** | | | | | |
| --- | --- | --- | --- | --- | --- |
|  | *S. attenuata* | *S. clymene* | *S. coeruleoalba* | *S. frontalis* | *S. longirostris* |
| *S. attenuata* |  | 0.015 | 0.013 | 0.014 | 0.012 |
| *S. clymene* | 8.3 |  | 0.007 | 0.009 | 0.010 |
| *S. coeruleoalba* | 6.86 | 3.34 |  | 0.008 | 0.009 |
| *S. frontalis* | 6.69 | 3.89 | 3.38 |  | 0.010 |
| *S. longirostris* | 5.45 | 4.8 | 4.11 | 4.12 |  |
| **Cyt b (585 bp)** | | | | | |
|  | *S. attenuata* | *S. clymene* | *S. coeruleoalba* | *S. frontalis* | *S. longirostris* |
| *S. attenuata* |  | 0.008 | 0.008 | 0.008 | 0.009 |
| *S. clymene* | 3.6 |  | 0.003 | 0.005 | 0.009 |
| *S. coeruleoalba* | 3.84 | 1.4 |  | 0.004 | 0.009 |
| *S. frontalis* | 3.47 | 1.76 | 1.8 |  | 0.008 |
| *S. longirostris* | 4.38 | 4.51 | 4.63 | 3.71 |  |
| **Cox 1 (621bp)** | | | | | |
|  | *S. attenuata* | *S. clymene* | *S. coeruleoalba* | *S. frontalis* | *S. longirostris* |
| *S. attenuata* |  | 0.007 | 0.007 | 0.007 | 0.007 |
| *S. clymene* | 4.01 |  | 0.003 | 0.005 | 0.006 |
| *S. coeruleoalba* | 3.76 | 1.96 |  | 0.004 | 0.006 |
| *S. frontalis* | 3.97 | 2.36 | 1.79 |  | 0.006 |
| *S. longirostris* | 4.13 | 3.41 | 3.01 | 2.9 |  |
