# Supplementary material for: Mitochondrial diversity and inter-specific phylogeny among dolphins of the genus Stenella in the Southwest Atlantic Ocean
Source: PLoS One. 2022 Jul 14;17(7):e0270690. doi: 10.1371/journal.pone.0270690 (PMC9282552; doi:10.1371/journal.pone.0270690)
Supplement: S5 Table — (DOCX) [file pone.0270690.s012.docx]

**S5 Table Blasts of GenBank and DNA Surveillance for haplotypes positioned in clades of species different than their morphological identification and haplotypes represented by sequences of different species.**

| **Dloop** | | | | | | |
| --- | --- | --- | --- | --- | --- | --- |
| Haplotype | Sequence ID | Morphology | DNA Surveillance | GenBank | Location | Author |
| DLOOP_120 | Scl_33 | Scl | SclyZ4185 0.0112 | 100% Scl | SWA | This study |
|  | GQ504147 | Scl | SclyZ4185 0.0117 | 99% Scl | NWA | Kingston et al.,2009 |
| DLOOP_121 | Scl_34 | Scl | SclyZ4185 0.0015 | 99% Scl | SWA | This study |
| DLOOP_137 | Sco_03 | Sco | SclyZ4185 0.0112 | 99% Scl | SWA | This study |
| DLOOP_114 | Scl_10 | Scl | Scly8 0,0037 | 99% Scl | SWA | This study |
| DLOOP_134 | DQ845446 | Scl | Scly8 0.0 | 99% Scl | NWA | Kingston et al 2009 |
| DLOOP_129 | GQ504143 | Scl | Scly8 0.0078 | 99% Scl | NWA | Kingston et al 2009 |
| DLOOP_155 | GQ504161 | Sco | Scly8 0.0394 | 99%Sco | NWA | Kingston et al 2009 |
| DLOOP_147 | GQ504153 | Sco | Scly8 0.0353 | 99%Sco | NWA | Kingston et al 2009 |
| DLOOP_200 | EF682716 | Sfr | SattNP0.PR 0.0336 | 99% Sat | NEA | Querouil et al.,2010 |
| DLOOP_209 | EF682753 | Sfr | SattNP0.PR 0.0222 | 99% Sat | NEA | Querouil et al.,2010 |
| DLOOP_177 | EF682656 | Sfr | Satt5 0.0336 | 98% Sat | NEA | Querouil et al.,2010 |
|  | EF682734 | Sfr | Satt5 0.0336 | 98% Sat | NEA | Querouil et al.,2010 |
|  | EF682829 | Sfr | Satt5 0.0336 | 98% Sat | NEA | Querouil et al.,2010 |
| DLOOP_199 | EF682715 | Sfr | Satt5 0.0185 | 98% Sat | NEA | Querouil et al.,2010 |
| DLOOP_223 | EF682804 | Sfr | Satt1 0.0038 | 97% Sat | NEA | Querouil et al.,2010 |
| DLOOP_188 | EF682681 | Sfr | Satt1 0.0148 | 99% Sat | NEA | Querouil et al.,2010 |
| DLOOP_214 | EF682764 | Sfr | Satt5 0.011 | 99% Sat | NEA | Querouil et al.,2010 |
| DLOOP_01 | Sat_01 | Sat | Satt 5 0,0074 | 100% Sat | SWA | This study |
|  | GQ504120 | Sat | Satt5 0.0078 | 99% Sat | NWA | Kingston et al 2009 |
|  | EF682658 | Sfr | Satt5 0.0078 | 99% Sat | NEA | Querouil et al.,2010 |
|  | EF682703 | Sfr | Satt5 0.0078 | 99% Sat | NEA | Querouil et al.,2010 |
|  | EF682748 | Sfr | Satt5 0.0078 | 99% Sat | NEA | Querouil et al.,2010 |
|  | EF682777 | Sfr | Satt5 0.0078 | 99% Sat | NEA | Querouil et al.,2010 |
|  | KC204739 | Sfr | Satt5 0.0078 | 99% Sat | NWA | Caballero et al.,2013 |
| DLOOP_3 | Sat_03 | Sat | Satt1 0,0037 | 99% Sat | SWA | This study |
|  | Sat_04 | Sat | Satt1 0,0037 | 99% Sat | SWA | This study |
|  | GQ504122 | Sat | Satt1 0.0039 | 99% Sat | NWA | Kingston et al 2009 |
|  | EF682659 | Sfr | Satt1 0.0039 | 99% Sat | NEA | Querouil et al.,2010 |
|  | EF682698 | Sfr | Satt1 0,0037 | 99% Sat | NEA | Querouil et al.,2010 |
|  | EF682705 | Sfr | Satt1 0.0039 | 99% Sat | NEA | Querouil et al.,2010 |
|  | EF682721 | Sfr | Satt1 0.0039 | 99% Sat | NEA | Querouil et al.,2010 |
|  | EF682789 | Sfr | Satt1 0.0039 | 99% Sat | NEA | Querouil et al.,2010 |
|  | EF682812 | Sfr | Satt1 0.0039 | 99% Sat | NEA | Querouil et al.,2010 |
|  | EF682825 | Sfr | Satt1 0.0039 | 99% Sat | NEA | Querouil et al.,2010 |
| DLOOP_93 | GQ504121 | Sat | Satt1 0.0039 | 99% Sat | NWA | Kingston et al 2009 |
|  | EF682667 | Sfr | Satt1 0.0039 | 99% Sat | NEA | Querouil et al.,2010 |
| DLOOP_253 | KC204736 | Sfr | Slon03FP01 0.0155 | 99 % Slo | NWA | Caballero et al.,2013 |
|  | GQ504169 | Slo | SfrontCCIR0103 0,0 | 100% Sfr | NWA | Kingston et al 2009 |
| **Cytb** | | | | | | |
| CYTB_10 | Scl_33 | Scl | Scly3.LD 0.0029 | 99% Scl | SWA | This study |
|  | Scl_34 | Scl | Scly3.LD 0.0029 | 99% Scl | SWA | This study |
|  | Scl_35 | Scl | Scly3.LD 0.0029 | 99% Scl | SWA | This study |
|  | Sco_01 | Sco | Scly3.LD 0,0024 | 99% Scl | SWA | This study |
|  | Sco_03 | Sco | Scly3.LD 0,0024 | 99% Scl | SWA | This study |
| CYTB_20 | EU580088 | Sco | SfroA0.LD 0.0125 | 99% Sco | NWA | Viricel et al.,2012 |
| CYTB_07 | Scl_10 | Scl | Scly3.LD 0,0087 | 99% Scl | SWA | This study |
| CYTB_19 | Sco_11 | Sco | ScoeCO01 0,0098 | 99% Sco | SWA | This study |
| CYTB_14 | Sco_05 | Sco | ScoeCO01 0,0122 | 99% Sco | SWA | This study |
| CYTB_15 | Sco_07 | Sco | ScoeCO01 0,049 | 99% Sco | SWA | This study |
| **CoxI** | | | | | | |
| COX1_15 | Scl_33 | Scl | - | 99% Scl | SWA | This study |
|  | Scl_34 | Scl | - | 99% Scl | SWA | This study |
|  | Scl_35 | Scl | - | 99% Scl | SWA | This study |
|  | Sco_01 | Sco | - | 99% Scl | SWA | This study |
|  | Sco_03 | Sco | - | 99% Scl | SWA | This study |
| COX1_31 | KF281695 | Sco | - | 99% Sco | NEA | Alfonsi et al.,2013 |
| COX1_26 | DQ466006 | Sco | - | 99% Sco | NEA | Amaral et al.,2007 |
|  | EU496342 | Sco | - | 99% Sco | NWA, GOM, NEA, NWP | Viricel et al.,2012 |
| COX1_27 | DQ466007 | Sco | - | 99% Scl | NEA | Amaral et al.,2007 |
| COX1_09 | Scl_08 | Scl | - | 99% Scl | SWA | This study |
| COX1_11 | Scl_10 | Scl | - | 99% Scl | SWA | This study |
| COX1_01 | Sat_01 | Sat | - | 98% Scl | SWA | This study |
