# Supplementary material for: Mitochondrial diversity and inter-specific phylogeny among dolphins of the genus Stenella in the Southwest Atlantic Ocean
Source: PLoS One. 2022 Jul 14;17(7):e0270690. doi: 10.1371/journal.pone.0270690 (PMC9282552; doi:10.1371/journal.pone.0270690)
Supplement: S6 Table — (DOCX) [file pone.0270690.s013.docx]

**S6 Table Skull measurements and meristics (following Perrin, 1975) of the specimen morphologically identified as *Stenella coeruleoalba* (GEMARS 0047, Sco03 in this study).**

| Measurements | GEMARS 0047 (Sco 03) |
| --- | --- |
|  | mm |
| 1. Condylobasal length (CBL) | 425.3 |
| 2. Length of rostrum | 245.6 |
| 3. Width of rostrum at base | 108.1 |
| 4. Width of rostrum 60 mm anterior to base | 71.1 |
| 5. Width of rostrum at midlength | 58.7 |
| 6. Width of rostrum at 3/4 length | 45.2 |
| 7. Width of premaxillaries at midlength | 27.6 |
| 8. Tip of rostrum to external nares | 287.0 |
| 9. Tip of rostrum to internal nares | 304.7 |
| 10. Greatest preorbital width | 183.7 |
| 11. Greatest postorbital width | 224.9 |
| 12. Greatest zygomatic width | 221.9 |
| 13. Greatest width of external nares | 46.0 |
| 14. Greatest width of internal nares | 61.0 |
| 15. Greatest premaxillary width | 81.3 |
| 16. Greatest parietal width | 166.3 |
| 17. Greatest length of left posttemporal fossa | 78.8 |
| 18. Greatest width of left posttemporal fossa | 59.0 |
| 19. Length of mandibular symphysis | 31.8 |
| 20. Greatest length of left ramus | 370.4 |
| 21. Length of lower left tooth row | 216.3 |
| 22. Length of upper left tooth row | 215.8 |
| 23. Number of teeth (upper left) | — |
| 24. Number of teeth (upper right) | — |
| 25. Number of teeth (lower left) | 44 |
| 26. Number of teeth (lower right) | 44 |
| 27. Greatest height of left ramus | 69.6 |
| 28. Length of left mandibular fossa | 121.3 |
