# Supplementary material for: Mitochondrial diversity and inter-specific phylogeny among dolphins of the genus Stenella in the Southwest Atlantic Ocean
Source: PLoS One. 2022 Jul 14;17(7):e0270690. doi: 10.1371/journal.pone.0270690 (PMC9282552; doi:10.1371/journal.pone.0270690)
Supplement: S7 Table — (DOCX) [file pone.0270690.s014.docx]

**S7 Table External measures (according to Perrin, 1975) of the specimen AQUASIS 02C1151/476 (Scl 10 in this study), morphologically identified as *Stenella clymene*.**

| Measurements | | AQUASIS 02C1151/476 (ScL10) |
| --- | --- | --- |
|  |  | mm |
| 1. Total length, from the end of the jaw to the central recess of the tail | | 199 |
| End of the maxilla to the middle of the eye | | 31 |
| 2. Jaw length, from the extreme to the base of the melon | | 9,5 |
| 3. Length of the mouth, from the end of the maxilla to the buccal commissure | | 25 |
| 4. End of maxilla to auditory meatus | | 33 |
| 5. End of the maxilla to the center of the vent | | 28 |
| 6. End of the maxilla to the base of the dorsal fin | | 80 |
| 7. End of the maxilla to the base of the pectoral fin | | 37 |
| 8. End of the maxilla to the center of the anal orifice | |  |
| 9. Maximum tail width | | 43 |
| 10. Pectoral fin length, from anterior insertion to end | | 38 |
| 11. Pectoral fin length, from posterior insertion to end | | 19 |
| 12. Maximum pectoral fin width | | 8 |
| 13. Dorsal fin base | | 28 |
| 14. Dorsal fin height | | 19 |
| 15. Body circumference at armpit level | | 91 |
| 16. Body circumference at the level of the anterior portion of the dorsal fin | | 98 |
| 17. Body circumference at anus level | | 57 |
| 18. Number of teeth of the right / left jaw | | 36/37 |
| 19. Number of teeth of the right / left jaw | | 38/36 |
|  |  | |
